# Author Correction: DCAF1-based PROTACs with activity against clinically validated targets overcoming intrinsic- and acquired-degrader resistance

**DOI:** 10.1038/s41467-024-51317-6

**Published:** 2024-08-19

**Authors:** Martin Schröder, Martin Renatus, Xiaoyou Liang, Fabian Meili, Thomas Zoller, Sandrine Ferrand, Francois Gauter, Xiaoyan Li, Frederic Sigoillot, Scott Gleim, Therese-Marie Stachyra, Jason R. Thomas, Damien Begue, Maryam Khoshouei, Peggy Lefeuvre, Rita Andraos-Rey, BoYee Chung, Renate Ma, Benika Pinch, Andreas Hofmann, Markus Schirle, Niko Schmiedeberg, Patricia Imbach, Delphine Gorses, Keith Calkins, Beatrice Bauer-Probst, Magdalena Maschlej, Matt Niederst, Rob Maher, Martin Henault, John Alford, Erik Ahrne, Luca Tordella, Greg Hollingworth, Nicolas H. Thomä, Anna Vulpetti, Thomas Radimerski, Philipp Holzer, Seth Carbonneau, Claudio R. Thoma

**Affiliations:** 1 Novartis Institutes for BioMedical Research, Basel, Switzerland; 2 Novartis Institutes for BioMedical Research, Cambridge, MA USA; 3 Friedrich Miescher Institute for Biomedical Research, Basel, Switzerland; 4 Present Address: Ridgeline Discovery, Basel, Switzerland; 5 Present Address: Swiss Institute for Experimental Cancer Research (ISREC), École Polytechnique Fédérale de Lausanne, Lausanne, Switzerland

**Keywords:** Chemical tools, Biophysics, Proteasome

Correction to: *Nature Communications* 10.1038/s41467-023-44237-4, published online 04 January 2024

The original version of this Article contained an error in the Methods section, which incorrectly read ‘Following incubation, the lysates were incubated with 35 μL (16)-functionalized resin 4 °C for 4 days.’ The correct version states ‘4 hours’ in place of ‘4 days.’

The original version of this Article contained errors in Figs. 5a and 6a regarding the chemical compounds. In the original version of Fig. 5a, the compound shown for DDa-1 and the compound shown for CDa-1 were incorrect. The correct version of Fig. 5 is:
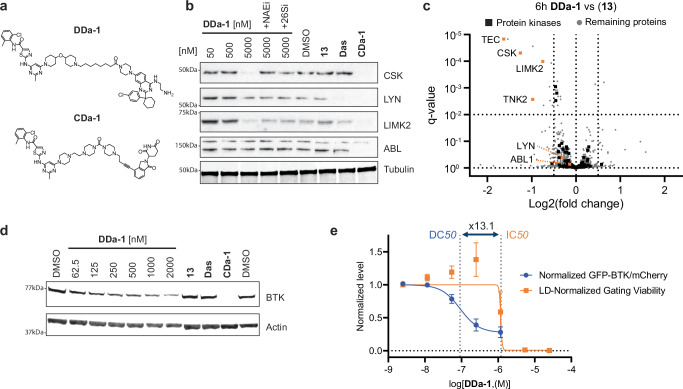


which replaces the previous incorrect version:
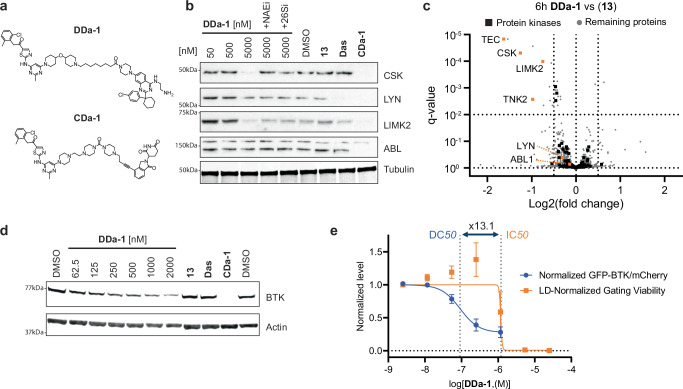


In the original version of Fig. 6a, the compound shown for DBt-5 and the compound shown for DBt-10 were incorrect. The correct version of Fig. 6 is:
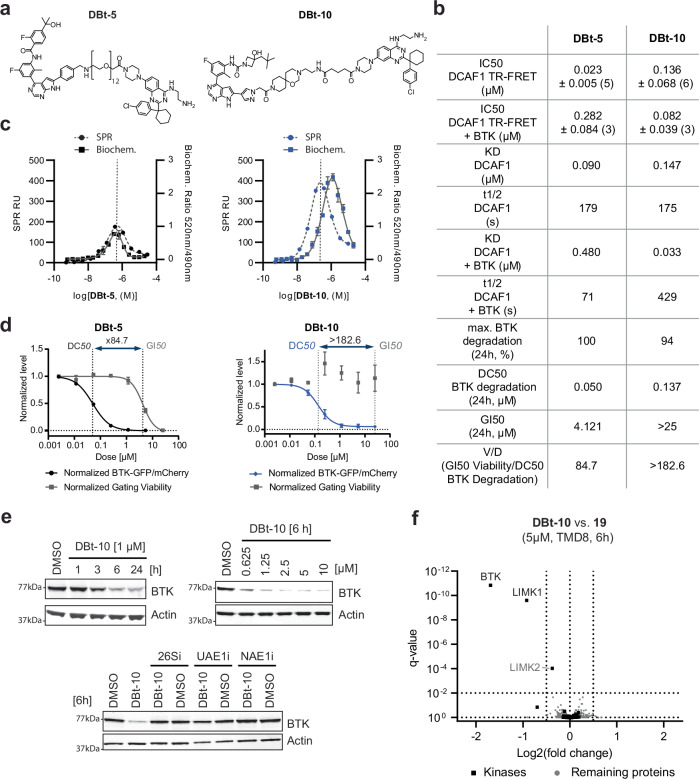


which replaces the previous incorrect version:
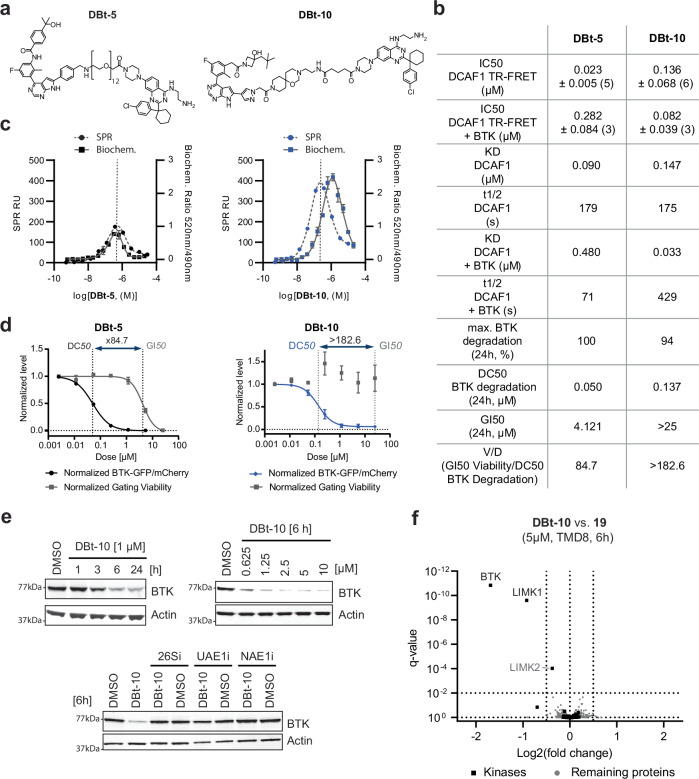


This has been corrected in both the PDF and HTML versions of the Article.

