# Supplementary material for: DCAF1-based PROTACs with activity against clinically validated targets overcoming intrinsic- and acquired-degrader resistance
Source: Nat Commun. 2024 Jan 4;15:275. doi: 10.1038/s41467-023-44237-4 (PMC10766610; doi:10.1038/s41467-023-44237-4)
Supplement: Supplementary file 1 — .Supplementary information [file 41467_2023_44237_MOESM1_ESM.pdf]

Supplementary information

DCAF1-based PROTACs with activity against clinically validated targets overcoming intrinsic- and acquired-degrader resistance

Martin Schröder[1,6,*], Martin Renatus[1,4,6], Xiaoyou Liang[2], Fabian Meili[2], Thomas Zoller[1] Sandrine Ferrand[1], Francois Gauter[1], Xiaoyan Li[2], Frederic Sigoillot[2], Scott Gleim[2], Therese-Marie Stachyra[1], Jason R. Thomas[2], Damien Begue[1], Maryam Khoshouei[1], Peggy Lefeuvre[1], Rita Andraos-Rey[1], BoYee Chung[2], Renate Ma[2], Benika Pinch[2], Andreas Hofmann[1], Markus Schirle[2], Niko Schmiedeberg[1], Patricia Imbach[1], Delphine Gorses[1], Keith Calkins[1], Beatrice Bauer-Probst[1], Magdalena Maschlej[1], Matt Niederst[2], Rob Maher[2], Martin Henault[2], John Alford[2], Erik Ahrne[1], Luca Tordella[1], Greg Hollingworth[1], Nicolas H. Thomä[3,5], Anna Vulpetti[1], Thomas Radimerski[1,4], Philipp Holzer[1], Seth Carbonneau[2], Claudio R. Thoma[2,4,*]

[1]Novartis Institutes for BioMedical Research, Basel, Switzerland.

[2]Novartis Institutes for BioMedical Research, Cambridge, USA.

[3]Friedrich Miescher Institute for Biomedical Research, Basel, Switzerland.

[4]Present address: Ridgeline Discovery, Basel, Switzerland

[5]Present address: Swiss Institute for Experimental Cancer Research (ISREC), École Polytechnique Fédérale de Lausanne, Lausanne, Switzerland

[6]These authors contributed equally

*Correspondance : martin-1.schroeder@novartis.com cthoma@ridgelinediscovery.com

SUPPLEMENTARY FIGURES **S3**

Suppl. Figure 1. Characterization of **13-N**, the exit vector and chemical proteomics of compound 16 **S3**

Suppl. Figure 2. Additional biochemical and cellular characterization of **DBr-1** **S5**

Suppl. Figure 3. Characterization of **DBr-1-N** **S7**

Suppl. Figure 4. Competition experiments with **DBr-1** and **DBr-1-N** **S9**

Suppl. Figure 5. Genetic BRD9 degradation rescue screen **S10**

Suppl. Figure 6. Establishment and validation of a ratio-metric BTK-GFP assay in TMD8 cells **S12**

Suppl. Figure 7. Biochemical/biophysical characterization of DCAF1-based BTK degrader **S14**

Suppl. Figure 8. SPR data of binary and ternary DCAF1-BTK PROTACs **S15**

Suppl. Figure 9. Assessment of BTK inhibition and cellular target engagement of DCAF1-BTK PROTACs **S16**

Suppl. Figure 10. Validation of PROTAC-mediated DCAF1 activity in cells with acquired resistance to CRBN-based PROTACs **S17**

SUPPLEMENTARY TABLES **S19**

SUPPLEMENTARY METHODS: Chemistry **S21**

Synthesis of **13-N** S21

Synthesis of **DBr-1** S22

Synthesis of **DBr-1-N** S26

Synthesis of **DDa-1** S27

Synthesis of **DBt-5** S31

Synthesis of **DBt-10** S35

NMR spectra S45

REFERENCES **S67**

SUPPLEMENTARY FIGURES

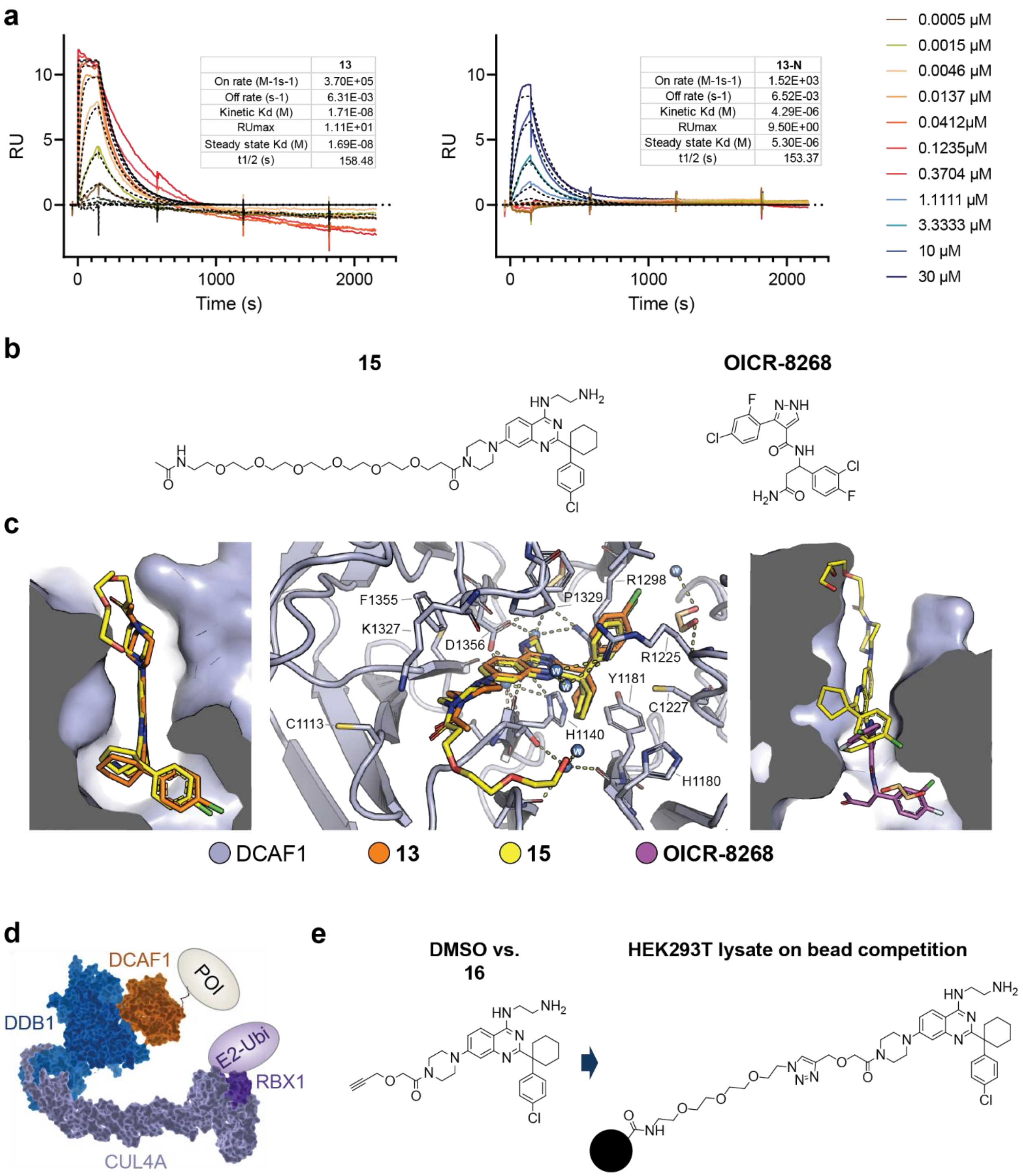

Supplementary Figure 1. Characterization of 13-N, the exit vector and chemical proteomics of compound 16

a) Examples of SPR sensorgrams with surface immobilized DCAF1(WDR) and (**13**) or (**13-N**) as analytes. The fitting curves are presented as black dashed lines. The inlets display the kinetic and steady state fit parameters of the shown presented here.

b) Structure of PEGylated DCAF1 binder (**15**) and the DCAF1 ligand **OICR-8268 [1]** .
c) Left panel: Surface representation of the complex of DCAF1 with (**13**) (orange, pdb ID 8OO5), and (**15**) (yellow, pdb ID: 8OOD). Middle panel: Detailed representation of the binding mode of (**15**) (yellow, pdb ID: 8OO5) overlayed with (**13**) (orange, pdb ID 8OOD). Hydrogen bonds are indicated as dashes. Right panel: Overlay of the binding mode of (**15**) and **OICR-8268[1]** (magenta, pdb ID: 8F8E).
d) Model of the relative position of the exit vector of (**15**) based on an overlay of (**15**)-DCAF1 complex (PDB ID: 8OO5, full linker modeled) with the DCAF1-DDB1-Cul4-RBX1 complex (PDB ID: 7OKQ). Spheres highlight the potential space occupied by the protein-of-interest (POI) and the E2-Ubiquitin conjugate (E2-Ubi)
e) Structure and schematic of on-bead coupling of (**16**) for chemical proteomics.

Source data are provided as a Source Data file

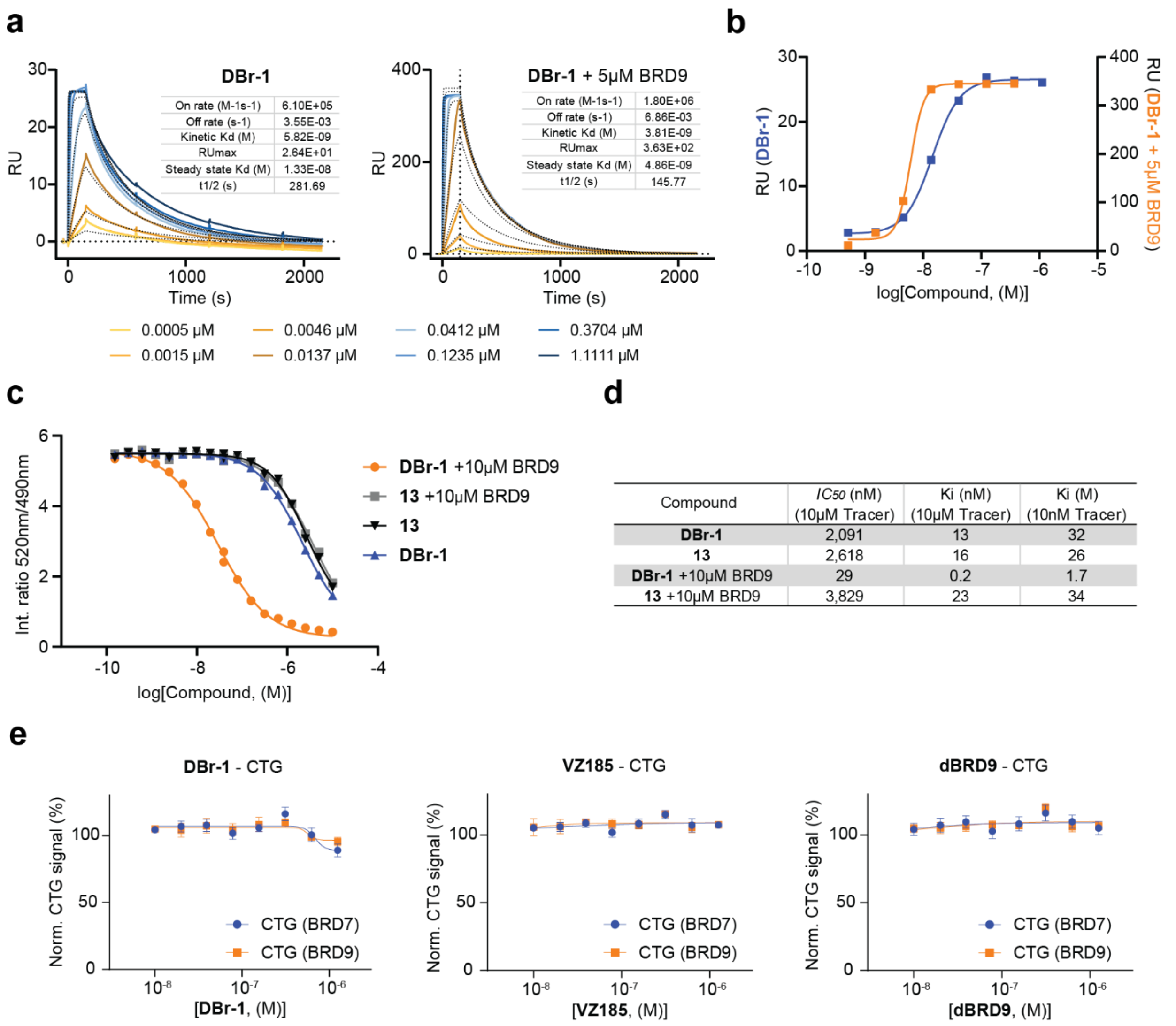

Compound	*IC50* (nM) (10µM Tracer)	Ki (nM) (10µM Tracer)	Ki (M) (10nM Tracer)
**DBr-1**	2,091	13	32
**13**	2,618	16	26
**DBr-1** +10µM BRD9	29	0.2	1.7
**13** +10µM BRD9	3,829	23	34

Supplementary Figure 2. Additional biochemical and cellular characterization of DBr-1

a) SPR sensorgrams with surface immobilized DCAF1(WDR) and **DBr-1** and **DBr-1** with additional 5µM BRD9-BD(130-250) as analytes. The fitting curves are presented as black dashed lines. The inlets display the kinetic and steady state fit parameters of the shown presented here.

b) SPR response of **DBr-1** alone (blue, left y-axis) or **DBr-1** with additional 5µM BRD9-BD(130-250) (orange, right y-axis) blotted against **DBr-1** concentration.

c) TR-FRET assay with higher concentration of tracer (10µM instead of 10nM). Shown are binding data of DCAF1(WDR), (**DBr-1**, blue) and (**DBr-1** with additional 10µM BRD9-BD(130-250), orange), (**13**, black) and (**13** with additional 10µM BRD9-BD(130-250), gray.

d) IC_{50} determined by DCAF1 TR-FRET for various compounds with additional 10µM BRD9-BD(130-250)) at 10µM Tracer concentration. Ki values of the IC_{50} values obtained with both tracer concentrations were calculated using the Cheng-Prusoff equation.

e) Cell titer Glow assay of HEK293 BRD7 or BRD9 HiBiT cells. Cells were treated with **DBr-1**, **VZ185** and **dBRD9** at various concentrations for 2h. Shown is the relative luminescence signal normalized to the DMSO controls. Data represents the average and standard deviation of 3 replicates.

Source data are provided as a Source Data file

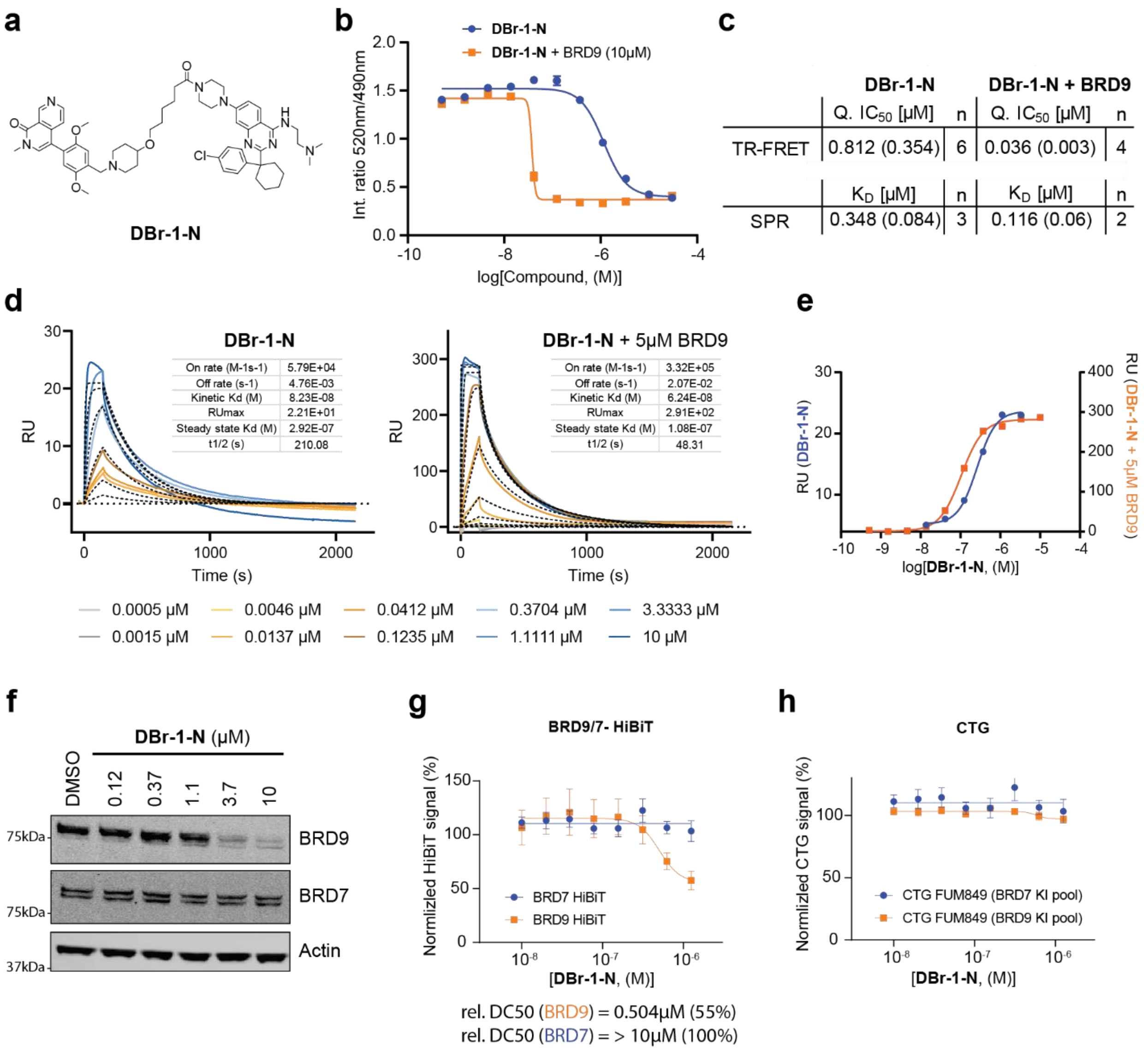

Supplementary Figure 3. Characterization of DBr-1-N

a) Chemical structure of **DBr-1-N**

b) TR-FRET assay binding data of DCAF1(WDR) and (**DBr-1-N**, blue) and (**DBr-1-N** with additional 10µM BRD9-BD(130-250), orange). Data represents mean ± standard deviation from n=4 replicates.

c) Affinity (K_D) determined by SPR and IC_{50} determined by DCAF1 TR-FRET for **DBr-1-N** and **DBr-1-N** with additional 10µM BRD9-BD(130-250).

d) SPR sensorgrams with surface immobilized DCAF1(WDR) and **DBr-1-N** and **DBr-1-N** with additional 5µM BRD9-BD(130-250) as analytes. The fitting curves are presented as black dashed lines. The inlets display the kinetic and steady state fit parameters of the SPR measurements presented here.

e) SPR response of **DBr-1-N** alone (blue, left y-axis) or **DBr-1-N** with additional 5µM BRD9-BD(130-250) (orange, right y-axis) blotted against **DBr-1-N** concentration.

f) Immunoblot analysis of HEK293 BRD9-HiBit/FF/CAS9 cells treated for 2h with **DBr-1-N** at various doses.

g) BRD9-HiBiT and BRD7-HiBiT signal detection of samples treated for 2h with **DBr-1**, **VZ185** and **dBRD9** at various doses. Data represents average ± standard deviation from n=3 replicates. DC50 values and maximal observed degradation are shown below.

h) Cell titer Glow assay of HEK293 BRD7 or BRD9 HiBiT cells. Cells were treated with **DBr-1**, **VZ185** and **dBRD9** at various concentrations for 2h. Shown is the relative luminescence signal normalized to the DMSO controls. Data represents average ± standard deviation from n=3 replicates.

Source data are provided as a Source Data file

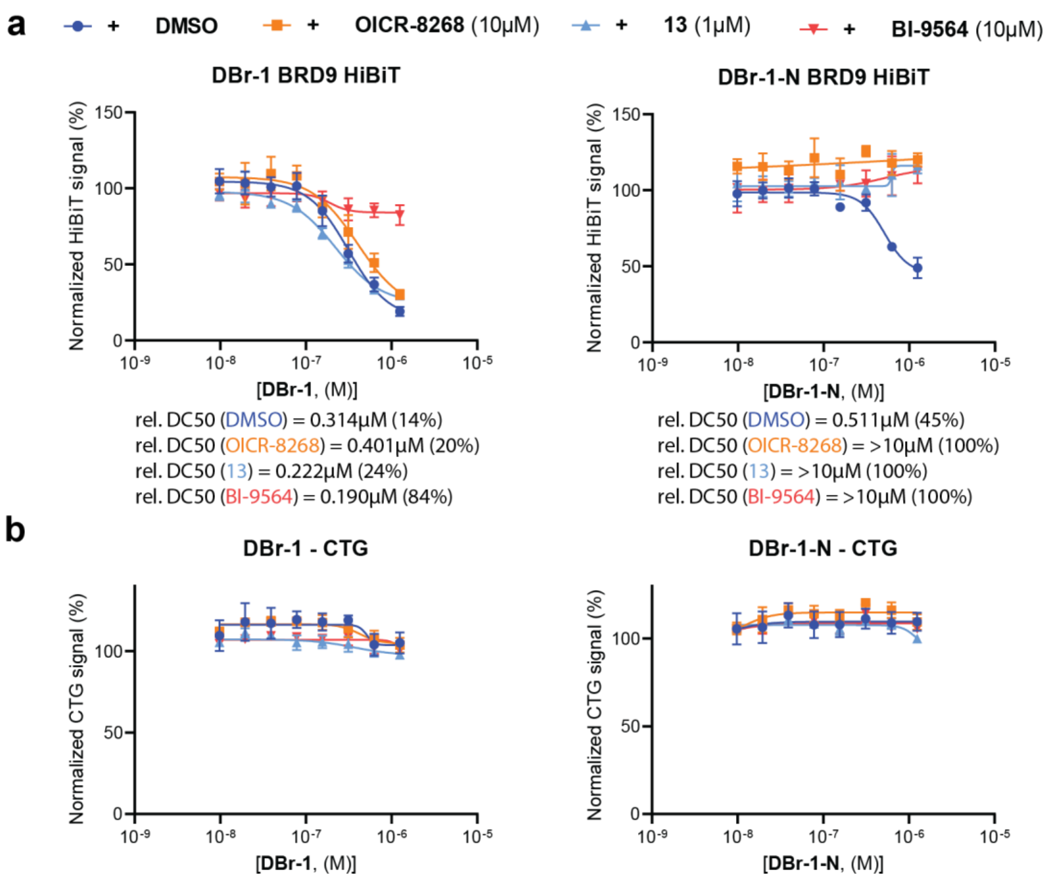

Supplementary figure 4. Competition experiments with DBr-1 and DBr-1-N

a) BRD9-HiBiT signal detection of samples pretreated with either DMSO, 10µM **OICR-8268**, 1µM (**13**) or 10µM **BI-9564** followed by a treatment with **DBr-1**, **VZ185** and **dBRD9** at various doses for 2h. Data represents average ± standard deviation from n=3 replicates. DC50 values and maximal observed degradation are shown below.

b) Cell titer Glow assay of BRD9 HiBiT cells. Cells pretreated with either DMSO, 10µM **OICR-8268**, 1µM (**13**) or 10µM **BI-9564** followed by a treatment with **DBr-1**, **VZ185** and **dBRD9** at various doses for 2h. Data represents average ± standard deviation from n=3 replicates.

Source data are provided as a Source Data file

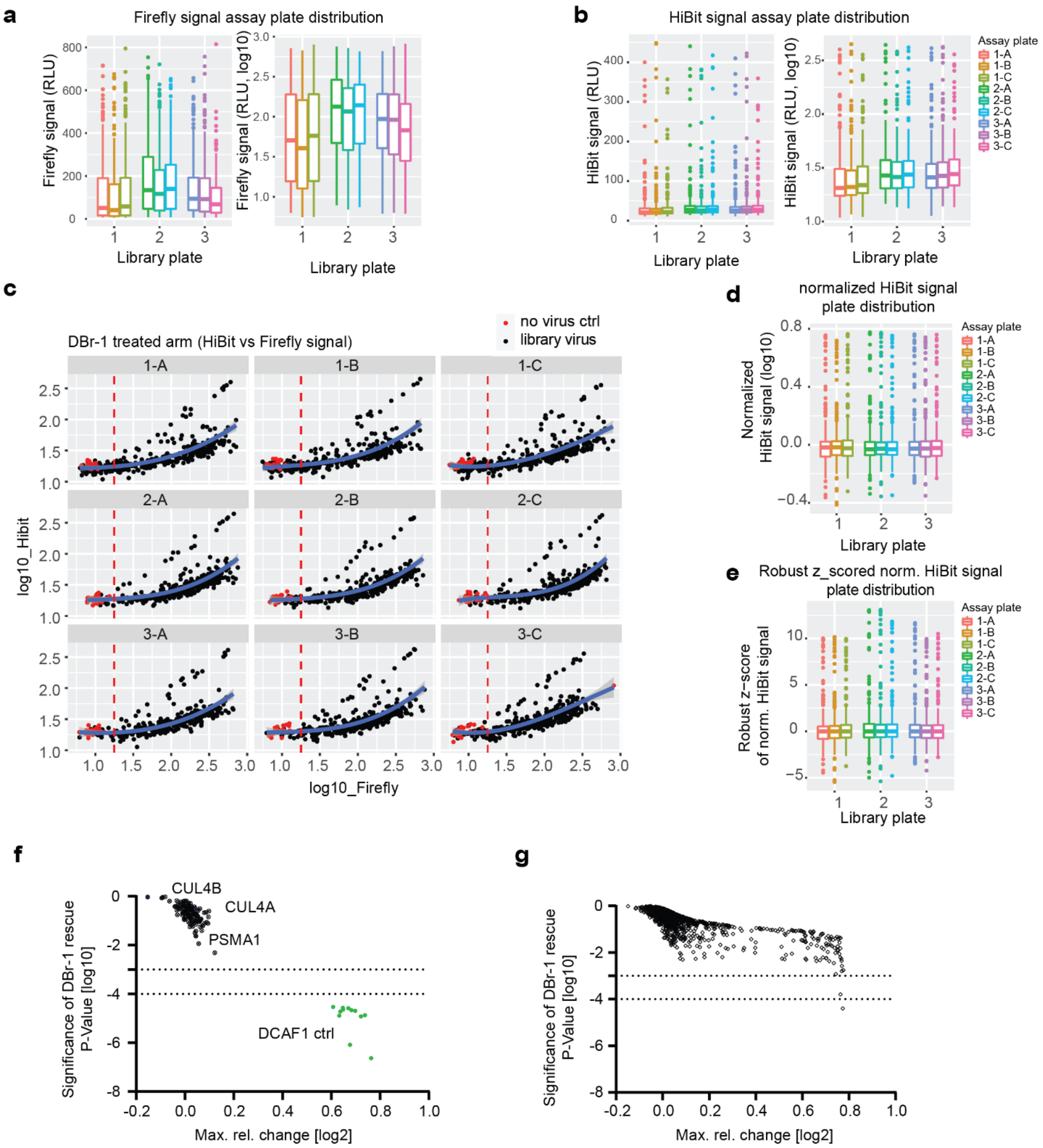

Supplementary Figure 5. Genetic BRD9 degradation rescue screen

a) Relative Firefly luciferase signal per assay plate depicted as box plots. From each sgRNA library plate 3 assay plates were stamped.

b) Relative HiBit luciferase signal per assay plate depicted as box plots. From each sgRNA library plate 3 assay plates were stamped.

c) Dot plot depicting individual Firefly (x-axis) and HiBit (y-axis) signals per assay plate (A-C) stamped in triplicate from library plates (1-3), each dot represent an individual well. Red dots depict no virus ctrl wells and black dots represent a library or ctrl virus treated well of the assay plate. The red line at

Firefly log10 1.25 represents the threshold for hit-calling. The blue line represents the assay plate based normalization curve (non-linear polynomial degree 3).

d) Box plots representing normalized HiBit signal to Firefly signal based on a non-linear polynomial degree 3 relationship (see (D)). From each sgRNA library plate 3 assay plates were stamped.

e) Box plots representing robust z-score from norm. HiBit signal (see (E)). From each sgRNA library plate 3 assay plates were stamped.

f) Ubiquitin sgRNA sublibrary rescue scores from (DBr-1) treatment plotted as significance of rescue P-value (y-axis) vs max. rel. change. Dotted lines at -4 and -3 P-value indicate strong and weaker hits with a false-discovery rate of 7%. Black dots represent sgRNA treatments of wells that did not pass the Firefly signal threshold of > log10 1.25, CUL4A, CUL4B and PSMA1 are indicated. Green dots represent DCAF1 ctrl sgRNAs distributes on each assay plate.

g) Randomized run of Ubiquitin sgRNA sublibrary screen results plotted as significance of rescue P-value (y-axis) vs max. rel. change. Dotted lines at -4 and -3 P-value indicate strong and weaker hits.

Source data are provided as a Source Data file

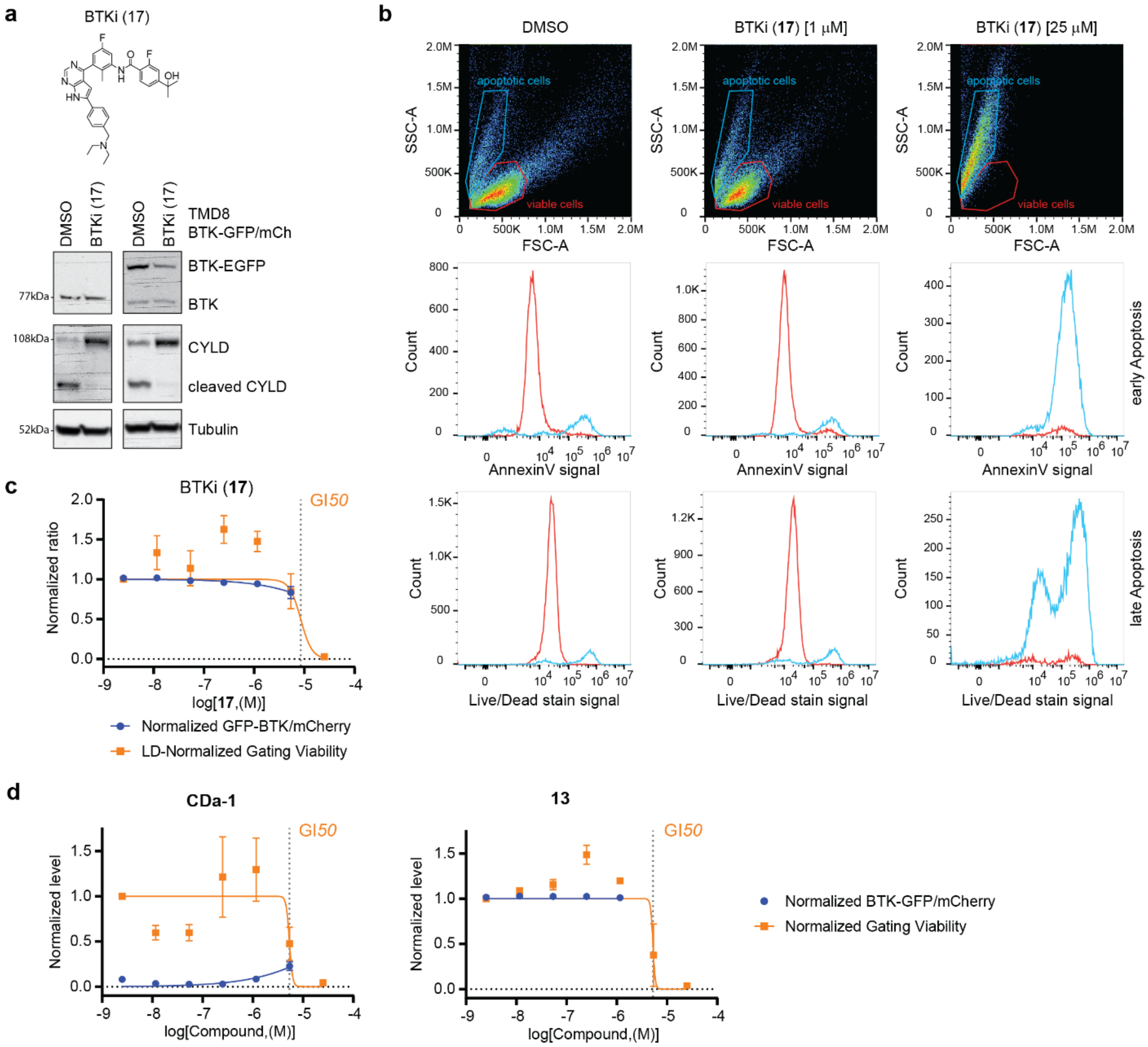

Compound ID	Degradation BTK-GFP/mCherry max. Deg	Abs. DC50 [µM]	DC50 [µM]	Viable Gate Inclusion Abs. GI50 [µM]	GI50 [µM]	Max concentration [µM] Gating Viability >25%	Avg #single cells Max concentraion	V/D (GI50 Viability / DC50 Degradation)
(**17**)	17%		>25	6.817	6.748	5.39	9197	0.3
(**13**)	0%		>25	5.22	5.044	1.16	15731	0.2
(**CDa-1**)	97%	<0.0025	<0.0025	9.882	5.312	5.39	6225	2124.8

Supplementary Figure 6. Establishment and validation of a ratio-metric BTK-GFP assay in TMD8 cells

a) Structure of BTKi (**17**) and characterization of TMD8 and TMD8 BTK-GFP/mCh cell lines after (**17**) treatment for 24 hours.

b) Dot plot representing Forward (FSC-A, x-axis) versus Sideward (SSC-A, y-axis) scattering of cells measured by flow cytometry treated with (**17**) at concentrations of 1 and 25 µM or DMSO as control. Each dot represents an individual cell, color code represents cell density in plot where single dot resolution was not possible (the warmer the color the more cells per plot region). Selection gates for viable (red) and apoptotic (blue) cells are indicated (first row). Histogram plots based on above gating strategy depicting individual cell count versus signal of an early and a late apoptotic marker (Annexin

V, middle row; Live/Dead stain, bottom row, respectively). Viable populations are plotted as red curves and apoptotic populations as blue curves.

c) Degradation of BTK-GFP in TMD8 BTK-GFP/mCh cells after 24h (**17**) treatment displayed as normalized rel. change of the ratio between BTK-GFP and mCherry (mCh) signals (blue curve), Viability after 24h **DDa-1** treatment is displayed as rel. change in cellular distribution between viable and apoptotic FSC/SSC gate (see (B)). DC50, GI50 and V/D is shown in (D). Data represents average ± standard deviation from n=3 replicates.

d) Degradation and viability of BTK-GFP in TMD8 BTK-GFP/mCh cells after 24h **CDa-1** or (**13**) treatment as in (C). Table depicting parameters extracted from flowcytometry measurements of TMD8 BTK-GFP/mCh cells treated with BTKi (**17**), DCAF1 binder (**13**) and CRBN-Das PROTAC **CDa-1**. Data represents average ± standard deviation from n=3 replicates.

Source data are provided as a Source Data file

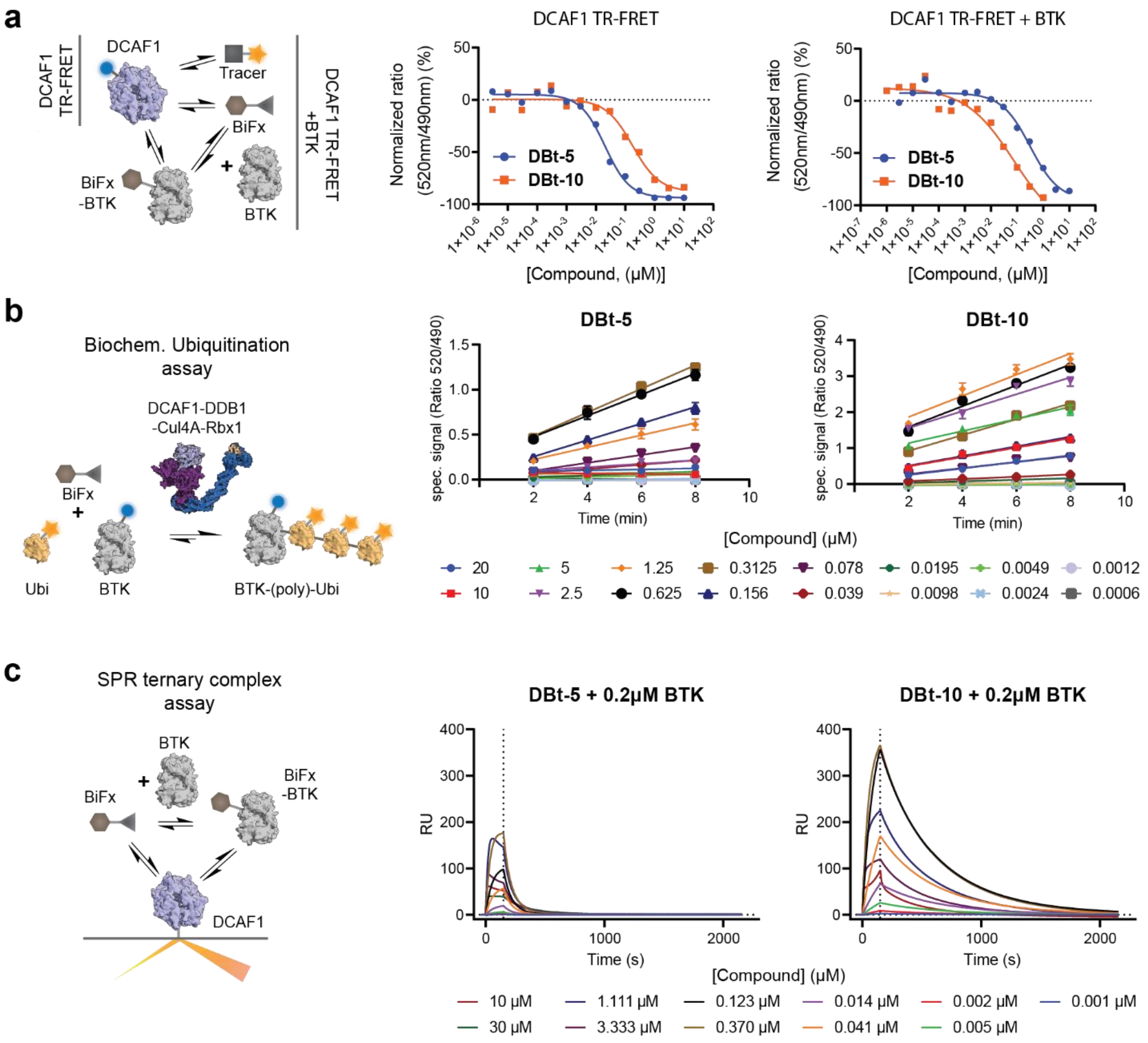

Supplementary figure 7. Biochemical/biophysical characterization of DCAF1-based BTK degrader

a) Schematic depiction of the DCAF1 TR-FRET assay and DCAF1 TR-FRET assay with additional 10μM BTK (left panel). TR-FRET assay binding data of DCAF1(WDR) and **DBt-5** or **DBt-10** and **DBt-5** or **DBt-10** with additional 10μM BTK

b) Schematic illustration of the biochemical ubiquitination assay based on a Ubiquitin-BTK TR-FRET pair (left panel). Initial measured velocities of the ubiquitination reaction of recombinant CUL4-DCAF1 in presence of **DBt-5** and **DBt-10** at various concentrations. Data represents average ± standard deviation from n=3 replicates.

c) Schematic illustration of the SPR-based ternary complex formation assay. SPR sensorgrams with surface immobilized DCAF1(WDR) and **DBt-5** and **DBt-10** with additional 0.2μM BTK as analytes.

Source data are provided as a Source Data file

a

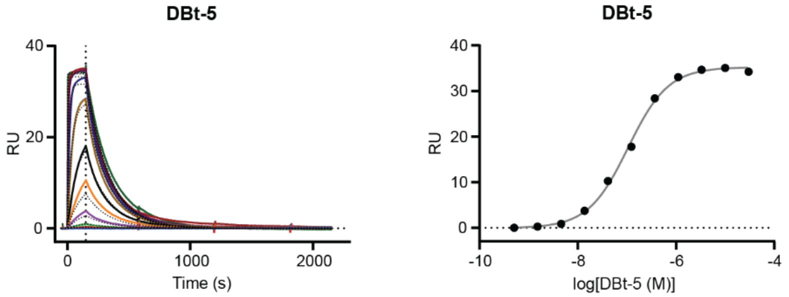

	DBt-5
On rate ($M^{-1}s^{-1}$)	6.22E+04
Off rate (s^{-1})	5.57E-03
Kinetic Kd (M)	8.96E-08
RUmax	34.1
Steady state Kd (M)	1.05E-07
$t^{1/2}$ (s)	179.53

b

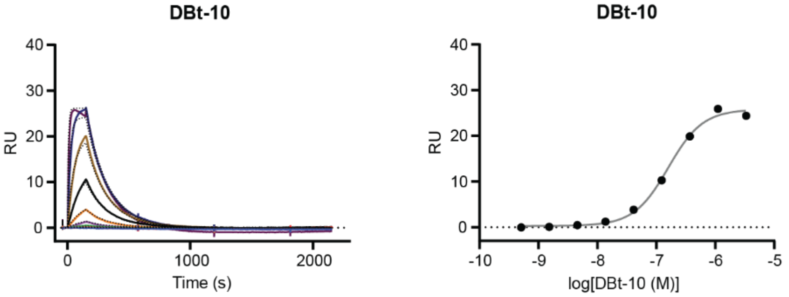

	DBt-10
On rate ($M^{-1}s^{-1}$)	3.88E+04
Off rate (s^{-1})	5.70E-03
Kinetic Kd (M)	1.47E-07
RUmax	27.3
Steady state Kd (M)	1.74E-07
t1/2 (s)	175.44

c

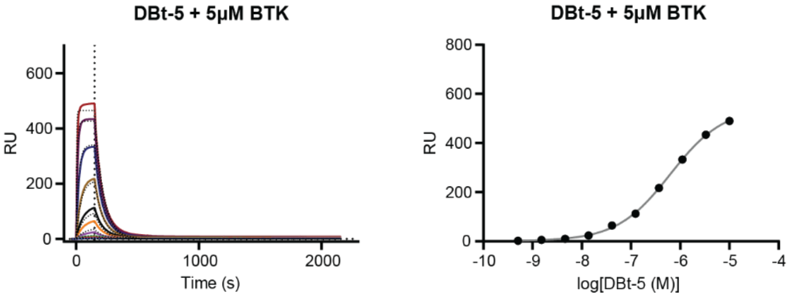

	DBt-5 + 5uM BTK
On rate ($M^{-1}s^{-1}$)	2.93E+04
Off rate (s^{-1})	1.40E-02
Kinetic Kd (M)	4.80E-07
RUmax	486.9
Steady state Kd (M)	5.29E-07
t1/2 (s)	71.43

d

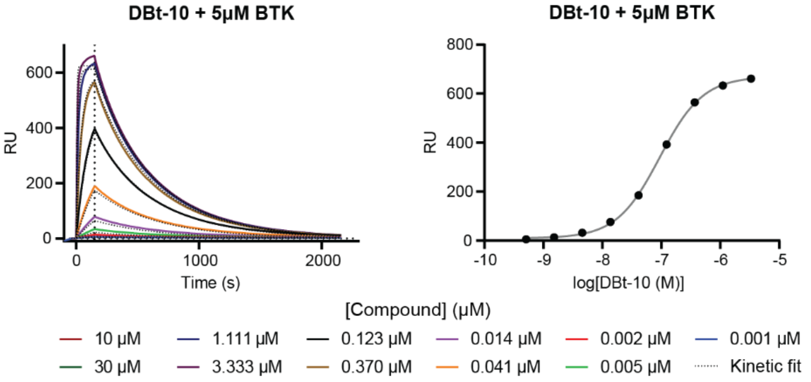

	DBt-10 + 5uM BTK
On rate ($M^{-1}s^{-1}$)	7.16E+04
Off rate (s^{-1})	2.33E-03
Kinetic Kd (M)	3.25E-08
RUmax	630.4
Steady state Kd (M)	9.77E-08
t1/2 (s)	429.18

Supplementary figure 8. SPR data of binary and ternary DCAF1-BTK PROTACs

(a)-(d) SPR sensorgrams with surface immobilized DCAF1(WDR) and **DBt-5** and **DBt-10** with and without additional BTK as analytes. The kinetic fitting curves are presented as black dashed lines. In the middle panel the steady state fit responses are blotted against the PROTAC concentrations The tables on the right display the kinetic and steady state fit parameters of the respective SPR measurements.

Source data are provided as a Source Data file

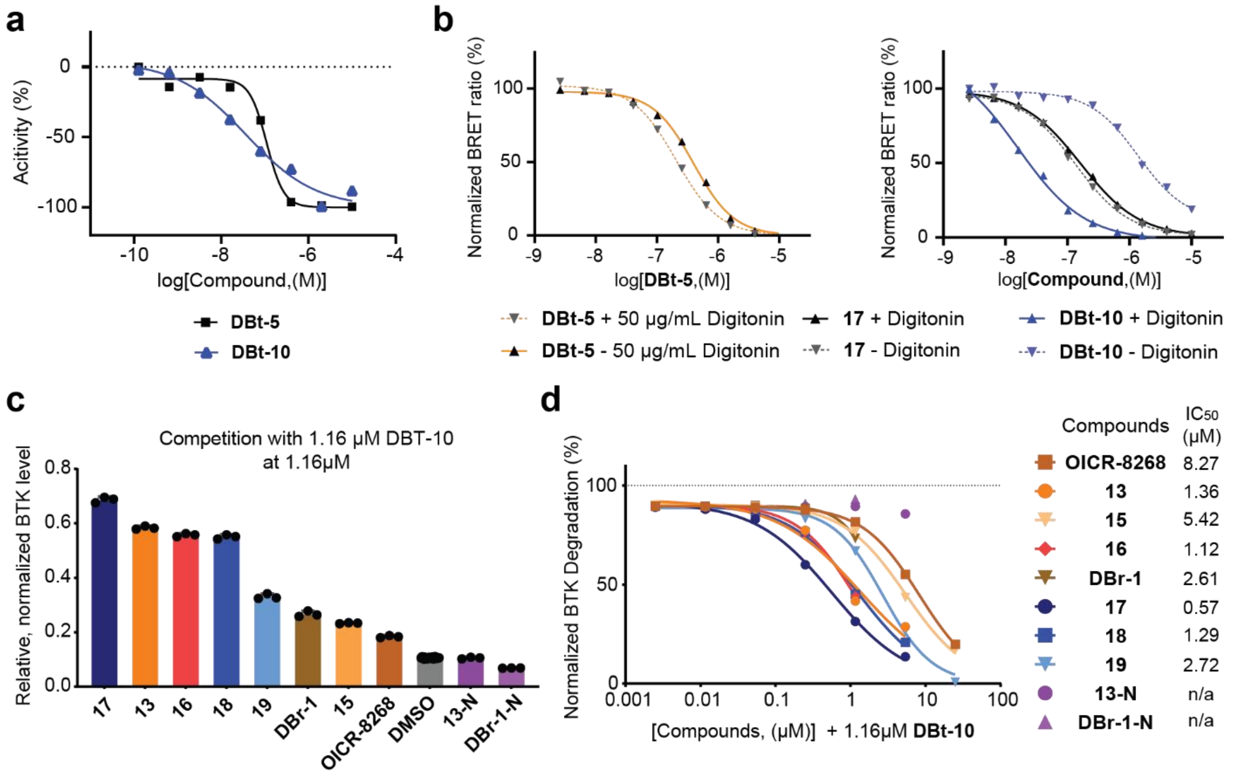

Supplementary figure 9. Assessment of BTK inhibition and cellular target engagement of DCAF1-BTK PROTACs

a) Characterization of PROTACs in a biochemical BTK enzymatic assay (IC50 values are displayed in Supplementary table 7).

b) Cellular BTK target engagement assays in the absence or presence of the cell permeabilizing agent Digitonin (IC50 values are displayed in Supplementary table 7).

c) Rescue of **DBt-10** (at 1.16µM) induced BTK degradation in TMD8 BTK-GFP/mCh cells by co-treatment of additional DCAF1 ligands or PROTACs at 1.16uM after 24h. Shown is the relative BTK-GFP levels normalized to the control lacking the DBt-10 treatment. Values shown are the average and standard deviation of three replicates.

d) Rescue of **DBt-10** (at 1.16µM) induced BTK degradation in TMD8 BTK-GFP/mCh cells by co-treatment of additional DCAF1 ligands or PROTACs at various concentrations after 24h. Shown is the relative BTK-GFP degradation. Values shown are the average and standard deviation of three replicates. IC50 values were calculated based on the inhibition of **DBt-10** mediated degradation of BTK at constant concentration of **DBt-10** at 1.16 µM. At >25% viable cell cut off was used to exclude confounding results due to cellular tox (See Method section).

Source data are provided as a Source Data file

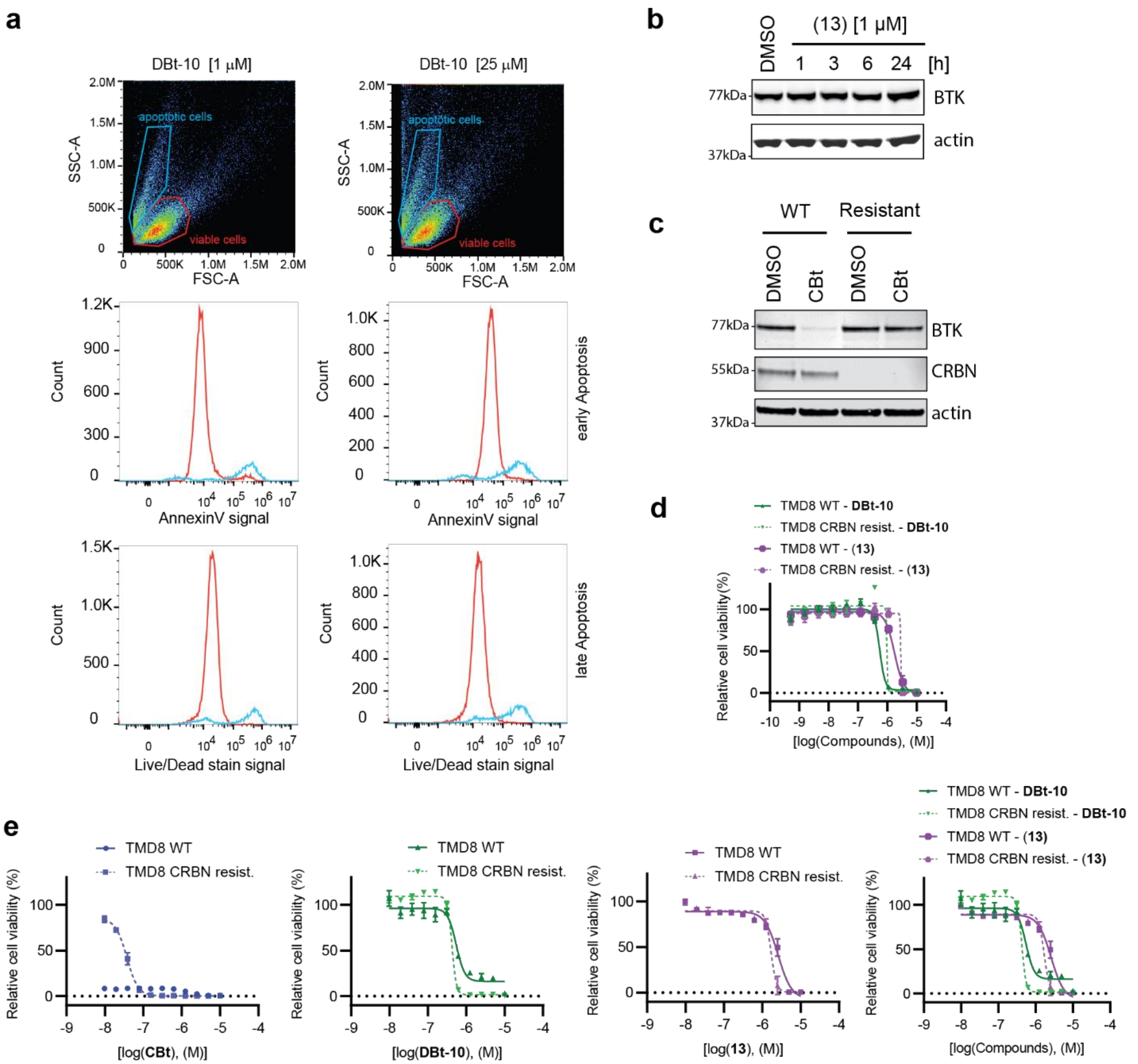

Supplementary figure 10. Validation of PROTAC-mediated DCAF1 activity in cells with acquired resistance to CRBN-based PROTACs

a) Dot plot representing Forward (FSC-A, x-axis) versus Sideward (SSC-A, y-axis) scattering of cells measured by flow cytometry treated with DBt-10 at concentrations of 1 and 25 µM. Each dot represents an individual cell, color code represents cell density in plot where single dot resolution was not possible (the warmer the color the more cells per plot region). Selection gates for viable (red) and apoptotic (blue) cells are indicated (first row). Histogram plots based on above gating strategy depicting individual cell count versus signal of an early and a late apoptotic marker (Annexin V, middle row; Live/Dead stain, bottom row, respectively). Viable cells are plotted as red curves and apoptotic cells as blue curves.

b) Immunoblot analysis of TMD8 cells treated with 1 µM (**13**) for 1, 3, 6 and 24 h time points.

c) Characterization of parental and CRBN resistant TMD8 cells probing for CRBN and BTK after 24h **CBt** treatment. Actin was used as loading control.

d) Viability measured by cell titer-Glo for **DBt-10** (green) vs (**13**) (purple) in WT (solid) vs CRBN resist. (dotted) TMD8 cells. Data represents average ± standard deviation from n=3 replicates.
e) Repeat of the viability measured by cell titer-Glo for **DBt-10** (green) vs (**13**) (purple) in WT (solid) vs CRBN resist. (dotted) TMD8 cells with adjusted dose response range. Data represents average ± standard deviation from n=3 replicates.

Source data are provided as a Source Data file

SUPPLEMENTARY TABLES

Supplementary table 1: Data collection and refinement statistics of the DCAF1-ligand complex X-ray structures

Supplementary table 1. Data collection and refinement statistics.

**Complex**	**DCAF1-(13)**	**DCAF1-(15)**
**PDB accession code**	**8OO5**	**8OOD**
**Beamline**	**SLS PXII**	**SLS PXII**
Data Collection		
Spacegroup	$P6_122$	$P6_122$
Cell dimensions	*a* = *b* = *81.6* Å, *c* = *234.1*	*a* = b = 81.5 Å, *c* = 232.7 Å
	α = *γ* = 90.0˚; *β* = 120˚	*α* = *γ* = 90.0˚; *β* = 120˚
Resolution (Å) [a]	52.38-2.25 (2.32-2.25)	52.20-1.50 (1.52-1.50)
No. unique reflections [a]	22,902 (2,066)	74,179 (3,405)
Completeness[a] (%)	100.0 (100.0)	99.6 (94.0)
I/σI[a]	11.4 (2.2)	11.1 (1.1)
R_{merge}[a]	0.280 (2.821)	0.091 (1.726)
CC (1/2) [a]	0.996 (0.771)	0.997 (0.452)
Redundancy[a]	21.8 (22.9)	8.7 (7.9)
Refinement		
No. atoms in refinement (P/L/W)[b]	2,383/88/77	2,570/136/316
B factor (P/L/W)[b] (Å^2)	43/58/45	21/32/33
R_{fact} (%)	18.1	17.0
R_{free} (%)	22.0	18.6
Rmsd [c] (Å)	0.017	0.021
Rmsd [c] angle (°)	2.08	2.5
Molprobity Ramachandran		
Favoured (%)	95.95	97.30
Outliers (%)	0.34	0

[a] Values in brackets show the statistics for the highest resolution shell.

[b] P/L/W indicate protein, ligand molecules, and water molecules, respectively.

[c] Rmsd indicates root-mean-square deviation

SUPPLEMENTARY METHODS: Chemistry Experimental

Compound (13-N) Synthetic Methods:

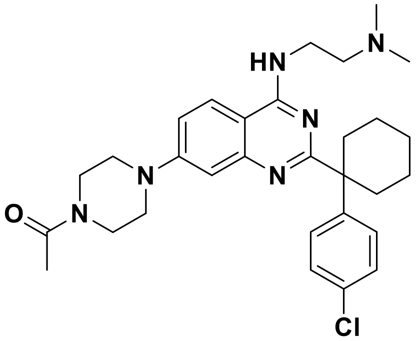

1-(4-(2-(1-(4-chlorophenyl)cyclohexyl)-4-((2-(dimethylamino)ethyl)amino)quinazolin-7-yl)piperazin-1-yl)ethan-1-one

In a pressure-release vial, 1-(4-(4-((2-aminoethyl)amino)-2-(1-(4-chlorophenyl)cyclohexyl)quinazolin-7-yl)piperazin-1-yl)ethan-1-one* (20.6 mg, 1 eq., 40.62 µmol) was dissolved in methanol (0.400 mL) at room temperature. Acetic acid (2.33 µL, 1 eq., 40.62 µmol) and formaldehyde (aqueous solution in water, 6.05 µL, 2 eq., 81.25 µmol) were added and the solution was stirred for four hours at room temperature. Sodium borohydride (3.07 mg, 2 eq., 81.25 µmol) was subsequently added. After 2.5 hours the reaction was purified by reverse-phase flash chromatography (Waters XBridge C18 OBD 30 x 50 mm column, 45-70% ACN/water with 5 mM NH_4OH modifier at 75 mL/min, 1.5mL injection). The fractions were lyophilized to afford the desired product (7.9 mg, 15 µmol, 36% yield) as a white fluffy solid.

^{1}H NMR (400 MHz, $CDCl_3$) δ 7.59 (d, *J* = 9.0 Hz, 1H), 7.47 – 7.41 (m, 2H), 7.20 – 7.15 (m, 2H), 7.09 (d, *J* = 2.5 Hz, 1H), 7.03 (dd, *J* = 9.0, 2.5 Hz, 1H), 3.78 (dd, *J* = 6.6, 4.0 Hz, 2H), 3.63 (m, *J* = 5.3 Hz, 4H), 3.35 (m, *J* = 13.0, 5.5 Hz, 4H), 2.79 (d, *J* = 12.9 Hz, 2H), 2.58 (t, *J* = 6.0 Hz, 2H), 2.31 (s, 6H), 2.15 (s, 3H), 2.05 (d, *J* = 11.5 Hz, 2H), 1.56 (m, *J* = 23.3, 11.6, 7.4 Hz, 5H), 1.39 (m, *J* = 8.7, 4.3 Hz, 1H).

13C NMR (151 MHz, CDCl3) δ 169.97, 169.09, 158.90, 153.30, 152.06, 146.96, 131.01, 128.37, 127.77, 121.90, 115.97, 110.91, 106.58, 57.90, 49.71, 48.46, 48.41, 45.91, 45.31, 41.12, 38.23, 35.75, 26.14, 23.37, 21.36.

LCMS: 535.3 [$M+H^+$].

*synthesis described previously in [2]

Compound DBr-1 Synthetic Methods:

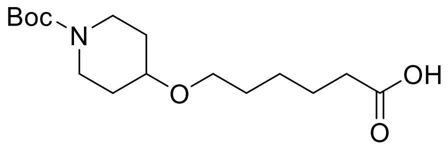

6-((1-(Tert-butoxycarbonyl)piperidin-4-yl)oxy)hexanoic acid

To a solution of tert-butyl 4-((6-methoxy-6-oxohexyl)oxy)piperidine-1-carboxylate* (15.0 g, 47.6 mmol, 1.0 Eq) in a mixture of MeOH (100 mL), THF (100 mL) and water (100 mL) at 20 °C was added lithium hydroxide hydrate (19.0 g, 475 mmol, 10.0 Eq) and the reaction mixture was heated at 80 °C for 2 hours. The mixture was concentrated, water (100 mL) was added and the pH was adjusted to 3 by the addition of a concentrated aqueous solution of citric acid. The mixture was extracted with ethyl acetate (2 x 200 mL), the combined organic phases were washed with brine (200 mL), dried over sodium sulfate and concentrated. The residue was purified by silica gel column chromatography, eluted with ethyl acetate (from 10 % to 15 %) in petrol ethers, yielding the title compound 6-((1-(tert-butoxycarbonyl)piperidin-4-yl)oxy)hexanoic acid as a yellow oil (12.4 g, 39.3 mmol, 86 %).

* synthesis described in patent WO2020/181050, page 145.

^{1}H NMR (400 MHz, DMSO-D$_6$) δ ppm: 12.05 – 11.84 (m, 1H), 3.66 – 3.53 (m, 2H), 3.10 – 2.89 (m, 2H), 2.24 – 2.14 (m, 2H), 1.80 – 1.69 (m, 2H), 1.55 – 1.43 (m, 4H), 1.43 – 1.35 (m, 9H), 1.35 – 1.25 (m, 4H).

MS: obs. m/z [M+H$^+$-Boc]: 216.2.

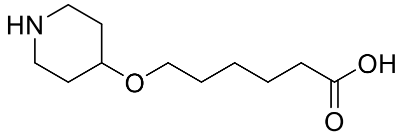

6-(Piperidin-4-yloxy)hexanoic acid

To a solution of 6-((1-(tert-butoxycarbonyl)piperidin-4-yl)oxy)hexanoic acid (1.0 g, 3.17 mmol, 1.0 Eq) in dichloromethane (10 mL) was added a solution of HCl in 1,4-dioxane (10 mL, 4 M, 40 mmol, 12.6 Eq), the reaction mixture was stirred at 30 °C for 1 hour and concentrated under reduced pressure, yielding the title compound 6-(piperidin-4-yloxy)hexanoic acid hydrochloride salt as a yellow solid (780 mg, 2.5 mmol, 78 %), which was used for the next step without further purification.

Mp: 104 – 107 °C.

^{1}H NMR (400 MHz, DMSO-D$_6$) δ ppm: 11.99 (br s, 1H), 8.98 – 8.72 (m, 2H), 3.60 – 3.46 (m, 1H), 3.41 – 3.35 (m, 2H), 3.14 – 3.03 (m, 2H), 2.93 (br s, 2H), 2.20 (br t, J = 7.3 Hz, 2H), 2.04 – 1.81 (m, 2H), 1.65 (br d, J = 8.9 Hz, 2H), 1.55 – 1.43 (m, 4H), 1.39 – 1.23 (m, 2H).

^{13}C NMR (100 MHz, DMSO-D$_6$) δ ppm: 175.0, 71.0, 67.7, 41.0, 34.2, 29.8, 27.9, 25.9, 24.9.

MS: obs. m/z [M+H$^+$]: 216.2.

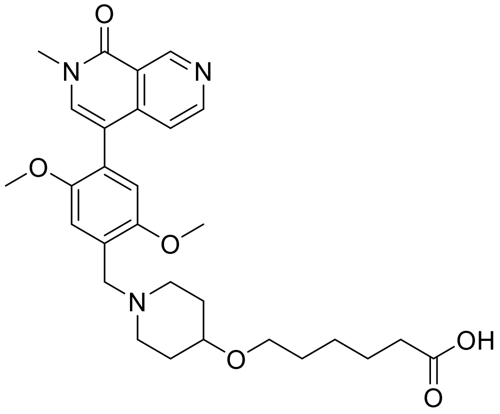

6-((1-(2,5-Dimethoxy-4-(2-methyl-1-oxo-1,2-dihydro-2,7-naphthyridin-4-yl)benzyl)piperidin-4-yl)oxy)hexanoic acid

To a solution of 6-(piperidin-4-yloxy)hexanoic acid (1.0 g, 4.64 mmol, 1.0 Eq) and 2,5-dimethoxy-4-(2-methyl-1-oxo-1,2-dihydro-2,7-naphthyridin-4-yl)benzaldehyde* (1.51 g, 4.64 mmol, 1.0 Eq) in ethanol (20 mL) was added N,N-diisopropyl ethylamine (1.8 g, 13.9 mmol, 3.0 Eq) and a solution of zinc chloride in THF (4.6 mL, 1 M, 4.64 mmol, 1.0 Eq) and the reaction mixture was stirred at 30 °C for 1 hour. Solid sodium cyanoborohydride (877 mg, 13.9 mmol, 3.0 Eq) was added and stirring was continued at 30 °C for 15 hours. Water (20 mL) was added, the mixture was extracted with dichloromethane (3 x 30 mL), the combined organic phases were washed with brine (2 x 10 mL) and dried over sodium sulfate. The residue was purified by preparative HPLC on a Phenomenex Luna C18 column (150 x 40 mm, 15 μm) eluting with acetonitrile (from 10 % to 40 %) in an aqueous solution of NH_4HCO_3 (0.1 %). Fractions containing the title compound were combined and freeze dried, yielding the title compound 6-((1-(2,5-dimethoxy-4-(2-methyl-1-oxo-1,2-dihydro-2,7-naphthyridin-4-yl)benzyl)piperidin-4-yl)oxy)hexanoic acid as a white solid (600 mg, 1.14 mmol, 25 %).

* synthesis described in patent WO2021/55295, page 195.

^{1}H NMR (400 MHz, DMSO-D$_6$) δ ppm: 9.40 (s, 1H), 8.63 (d, J = 5.6 Hz, 1H), 7.73 (s, 1H), 7.13 (s, 1H), 7.03 (d, J = 5.6 Hz, 1H), 6.91 (s, 1H), 3.74 (s, 3H), 3.63 (s, 3H), 3.56 (s, 3H), 3.52 – 3.46 (m, 2H), 3.37 (t, J = 6.4 Hz, 2H), 2.73 (br s, 2H), 2.24 – 2.11 (m, 4H), 1.84 (br d, J = 9.9 Hz, 2H), 1.54 – 1.41 (m, 6H), 1.35 – 1.25 (m, 2H), 1.01 – 0.94 (m, 2H).

^{13}C NMR (100 MHz, DMSO-D$_6$) δ ppm: 175.0, 161.1, 150.8, 150.6, 142.0, 138.6, 128.1, 122.1, 120.0, 118.7, 115.1, 113.6, 113.3, 74.8, 67.3, 55.8, 51.4, 36.8, 34.2, 31.7, 29.9, 25.9, 24.9.

MS: obs. m/z [M+H$^+$]: 524.5.

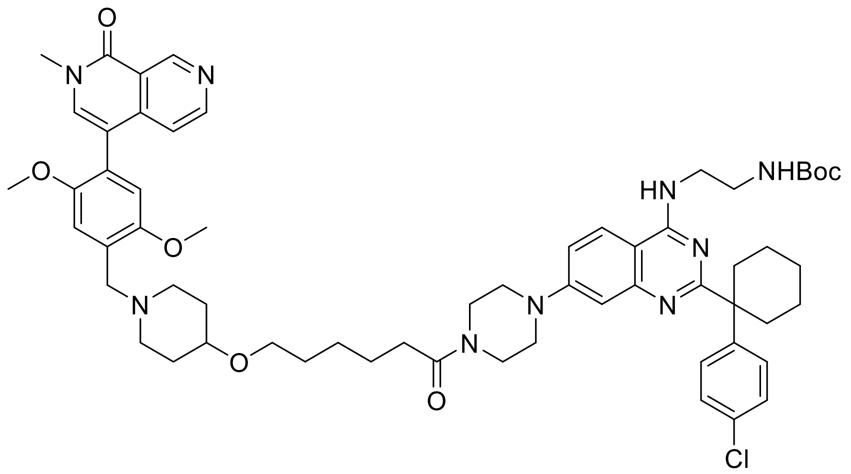

Tert-butyl (2-((2-(1-(4-chlorophenyl)cyclohexyl)-7-(4-(6-((1-(2,5-dimethoxy-4-(2-methyl-1-oxo-1,2-dihydro-2,7-naphthyridin-4-yl)benzyl)piperidin-4-yl)oxy)hexanoyl)piperazin-1-yl)quinazolin-4-yl)amino)ethyl)carbamate

To a solution of 6-((1-(2,5-dimethoxy-4-(2-methyl-1-oxo-1,2-dihydro-2,7-naphthyridin-4-yl)benzyl)piperidin-4-yl)oxy)hexanoic acid (310 mg, 0.59 mmol, 1.0 Eq) and tert-butyl (2-((2-(1-(4-chlorophenyl)cyclohexyl)-7-(piperazin-1-yl)quinazolin-4-yl)amino)ethyl)carbamate (334 mg, 0.59 mmol, 1.0 Eq) in DMF (5 mL) was added EDCI (227 mg, 1.18 mmol, 2.0 Eq), HOBt (160 mg, 1.18 mmol, 2.0 Eq) and N,N-diisopropyl ethylamine (153 mg, 1.18 mmol, 2.0 Eq) and the reaction mixture was stirred at 25 °C for 2 hours. The mixture was diluted with water (20 mL), extracted with ethyl acetate (3 x 20 mL), the combined organic phases were washed with brine (2 x 20 mL) and dried over sodium sulfate. The residue was purified by preparative HPLC on a Phenomenex Luna C18 column (150 x 40 mm, 15 μm) eluting with acetonitrile (from 26 % to 56 %) in an aqueous solution of TFA (0.1 %). Fractions containing the title compound were combined and freeze dried, yielding the title compound tert-butyl (2-((2-(1-(4-chlorophenyl)cyclohexyl)-7-(4-(6-((1-(2,5-dimethoxy-4-(2-methyl-1-oxo-1,2-dihydro-2,7-naphthyridin-4-yl)benzyl)piperidin-4-yl)oxy)hexanoyl)piperazin-1-yl)quinazolin-4-yl)amino)ethyl)carbamate as a white solid (400 mg, 0.37 mmol, 63 %).

^{1}H NMR (400 MHz, DMSO-D$_6$) δ ppm: 12.62 (br s, 1H), 9.76 (br s, 2H), 9.45 (d, J = 4.5 Hz, 1H), 8.69 – 8.65 (m, 1H), 8.15 (br d, J = 9.5 Hz, 1H), 7.83 (s, 1H), 7.48 (br d, J = 8.5 Hz, 2H), 7.42 – 7.39 (m, 2H), 7.34 (d, J = 12.5 Hz, 1H), 7.12 (s, 2H), 7.10 – 7.02 (m, 2H), 4.34 (br s, 2H), 3.83 (d, J = 2.1 Hz, 3H), 3.73 – 3.63 (m, 6H), 3.59 (br d, J = 1.1 Hz, 7H), 3.49 (br s, 3H), 3.46 – 3.38 (m, 5H), 3.20 – 2.97 (m, 3H), 2.80 – 2.69 (m, 2H), 2.43 – 2.28 (m, 3H), 2.18 – 2.03 (m, 3H), 2.03 – 1.77 (m, 3H), 1.63 (br s, 3H), 1.52 (td, J = 6.6, 13.3 Hz, 7H), 1.31 (s, 9H), 1.25 – 1.05 (m, 2H).

^{13}C NMR (100 MHz, DMSO-D$_6$) δ ppm: 160.9, 152.6, 151.2, 149.9, 149.2, 143.5, 142.6, 139.9, 132.1, 129.1, 128.8, 125.8, 120.1, 118.9, 116.7, 115.8, 112.8, 98.5, 78.3, 67.8, 56.8, 56.3, 53.8, 49.4, 37.0, 28.6.

HR MS: obs. m/z [M+H$^+$]: calculated for $C_{60}H_{76}N_9O_7Cl$, 1070.5556; found, 1070.5660; deviation: -2.9 ppm.

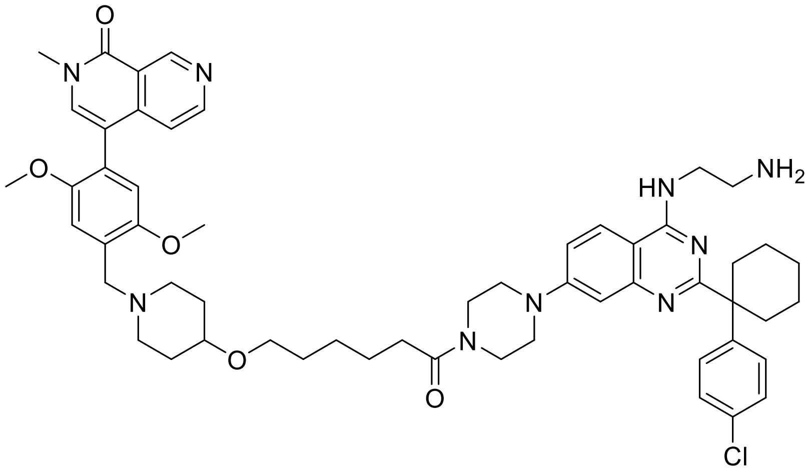

4-(4-((4-((6-(4-(4-((2-Aminoethyl)amino)-2-(1-(4-chlorophenyl)cyclohexyl)quinazolin-7-yl)piperazin-1-yl)-6-oxohexyl)oxy)piperidin-1-yl)methyl)-2,5-dimethoxyphenyl)-2-methyl-2,7-naphthyridin-1(2H)-one

To a solution of tert-butyl (2-((2-(1-(4-chlorophenyl)cyclohexyl)-7-(4-(6-((1-(2,5-dimethoxy-4-(2-methyl-1-oxo-1,2-dihydro-2,7-naphthyridin-4-yl)benzyl)piperidin-4-yl)oxy)hexanoyl)piperazin-1-yl)quinazolin-4-yl)amino)ethyl)carbamate (300 mg, 0.28 mmol, 1.0 Eq) in 1,4-dioxane (1 mL) was added a solution of HCl in 1,4-dioxane (1.0 mL, 4 M, 4.0 mmol, 14 Eq) at 25 °C and the reaction mixture was stirred for 2 hours. A saturated solution of $NaHCO_3$ (10 mL) was added, the mixture was extracted with DCM (3 x 10 mL), the combined organic phases were washed with brine (10 mL) and dried over sodium sulfate, yielding the title compound 4-(4-((4-((6-(4-(4-((2-aminoethyl)amino)-2-(1-(4-chlorophenyl)cyclohexyl)quinazolin-7-yl)piperazin-1-yl)-6-oxohexyl)oxy)piperidin-1-yl)methyl)-2,5-dimethoxyphenyl)-2-methyl-2,7-naphthyridin-1(2H)-one as a white solid (207 mg, 0.21 mmol, 76 %).

^{1}H NMR (400 MHz, DMSO-D$_6$) δ ppm: 9.40 (s, 1H), 8.64 (d, J = 5.6 Hz, 1H), 8.02 – 7.92 (m, 1H), 7.86 – 7.77 (m, 1H), 7.73 (s, 1H), 7.49 – 7.36 (m, 2H), 7.28 – 7.22 (m, 2H), 7.20 (dd, J = 2.3, 9.0 Hz, 1H), 7.13 (s, 1H), 7.03 (d, J = 5.8 Hz, 1H), 6.91 (s, 1H), 6.88 – 6.82 (m, 1H), 3.74 (s, 4H), 3.65 – 3.62 (m, 3H), 3.60 (br s, 4H), 3.56 (s, 3H), 3.50 (br s, 2H), 3.44 – 3.36 (m, 4H), 3.29 (br s, 4H), 2.83 (br s, 2H), 2.78 – 2.65 (m, 4H), 2.36 (br t, J = 7.4 Hz, 2H), 2.15 (br t, J = 9.9 Hz, 2H), 1.99 – 1.76 (m, 5H), 1.59 – 1.41 (m, 12H), 1.39 – 1.23 (m, 4H).

^{13}C NMR (100 MHz, DMSO-D$_6$) δ ppm: 171.2, 161.1, 152.8, 152.1, 151.4, 150.8, 150.6, 148.0, 142.0, 138.6, 130.5, 128.7, 128.2, 128.1, 123.9, 122.1, 120.0, 118.7, 116.1, 115.2, 113.6, 113.3, 109.2, 106.1, 74.9, 67.4, 56.5, 56.2, 55.8, 51.4, 44.7, 41.1, 36.9, 32.7, 25.1.

HR MS: obs. m/z [M+H$^+$]: calculated for $C_{55}H_{68}N_9O_5Cl$, 970.5032; found, 970.5134; deviation: -2.9 ppm.

DBr-1-N

4-(4-((4-((6-(4-(2-(1-(4-chlorophenyl)cyclohexyl)-4-((2-(dimethylamino)ethyl)amino)quinazolin-7-yl)piperazin-1-yl)-6-oxohexyl)oxy)piperidin-1-yl)methyl)-2,5-dimethoxyphenyl)-2-methyl-2,7-naphthyridin-1(2H)-one

In a pressure-release vial, 4-(4-((4-((6-(4-(4-((2-aminoethyl)amino)-2-(1-(4-chlorophenyl)cyclohexyl)quinazolin-7-yl)piperazin-1-yl)-6-oxohexyl)oxy)piperidin-1-yl)methyl)-2,5-dimethoxyphenyl)-2-methyl-2,7-naphthyridin-1(2H)-one (21.5 mg, 1 eq., 22.15 µmol) was dissolved in methanol (0.300 mL) at room temperature. Acetic acid (1.27 µL, 1 eq., 22.15 µmol) and formaldehyde (aqueous solution in water, 4.12 µL, 2.5 eq., 55.37 µmol) were added and the solution was stirred for four hours at room temperature. Sodium borohydride (2.51 mg, 3 eq., 66.45 µmol) was subsequently added. After 2 hours the reaction was purified by reverse-phase flash chromatography (Waters XBridge C18 OBD 30 x 50 mm column, 65-95% ACN/water with 5 mM NH_4OH modifier at 75 mL/min, 1.5mL injection). The fractions were lyophilized to afford the desired product (6.7 mg, 6.7 µmol, 30% yield) as a white fluffy solid.

^{1}H NMR (600 MHz, CDCl3) δ 9.67 (d, J = 0.9 Hz, 1H), 8.65 (d, J = 5.6 Hz, 1H), 7.71 (d, J = 9.1 Hz, 1H), 7.40 – 7.36 (m, 2H), 7.30 (s, 1H), 7.23 (s, 1H), 7.20 – 7.16 (m, 3H), 7.13 (s, 1H), 7.07 – 7.04 (m, 2H), 6.78 (s, 1H), 4.08 (s, 1H), 3.82 (s, 3H), 3.81 – 3.78 (m, 4H), 3.70 (s, 3H), 3.65 (s, 4H), 3.63 (d, J = 4.6 Hz, 1H), 3.55 (dd, J = 10.8, 6.4 Hz, 1H), 3.46 (t, J = 6.6 Hz, 2H), 3.37 (m, J = 18.7, 5.3 Hz, 5H), 3.09 (s, 2H), 2.87 (t, J = 5.4 Hz, 2H), 2.74 – 2.67 (m, 2H), 2.53 (s, 6H), 2.40 (t, J = 7.5 Hz, 2H), 2.11 (d, J = 14.8 Hz, 3H), 1.89 (s, 2H), 1.71 (m, J = 7.6 Hz, 3H), 1.65 (m, J = 6.8 Hz, 3H), 1.58 (q, J = 6.2 Hz, 4H), 1.52 (m, J = 10.3, 5.3 Hz, 1H), 1.48 – 1.40 (m, 4H).

^{13}C NMR (151 MHz, CDCl3) δ 171.49, 169.64, 163.28, 163.05, 162.82, 162.59, 161.65, 158.76, 153.66, 151.92, 151.76, 151.38, 150.53, 141.87, 136.69, 131.04, 128.54, 127.84, 122.77, 120.40, 119.65, 118.04, 117.71, 116.40, 115.77, 115.07, 114.40, 113.96, 113.83, 106.42, 68.13, 57.47, 56.19, 55.88, 54.35, 49.87, 48.39, 48.22, 45.15, 44.37, 41.17, 37.11, 36.94, 35.50, 33.10, 29.82, 26.08, 26.07, 24.99, 23.23, 1.13.

LCMS: 998.5 [M+H$^+$].

Compound DDa-1 Synthetic Methods:

Tert-butyl 4-((1-(6-((5-((2-chloro-6-methylphenyl)carbamoyl)thiazol-2-yl)amino)-2-methylpyrimidin-4-yl)piperidin-4-yl)oxy)piperidine-1-carboxylate

Tert-butyl 4-(piperidin-4-yloxy)piperidine-1-carboxylate* (0.11 g, 0.38 mmol, 3.0 Eq) was added to a suspension of 2-((6-chloro-2-methylpyrimidin-4-yl)amino)-N-(2-chloro-6-methylphenyl)thiazole-5-carboxamide** (50 mg, 0.13 mmol, 1.0 Eq) and N,N-diisopropyl ethylamine (44 µL, 0.25 mmol, 2.0 Eq) in 1,4-dioxane (1.2 mL) and the reaction mixture was heated at 90 °C for 24 hours. The mixture was diluted with water and ethyl acetate, the layers were separated, the aqueous layer was extracted with ethyl acetate and the combined organic layers were dried over sodium sulfate. The residue was purified by silica gel column chromatography, eluted with methanol (from 0 % to 15 %) in dichloromethane, yielding the title compound tert-butyl 4-((1-(6-((5-((2-chloro-6-methylphenyl)carbamoyl)thiazol-2-yl)amino)-2-methylpyrimidin-4-yl)piperidin-4-yl)oxy)piperidine-1-carboxylate as an off-white solid (57.1 mg, 0.089 mmol, 69 %).

* CAS: 845305-83-1 is commercially available.

** CAS: 302964-08-5 is commercially available .

^{1}H NMR (400 MHz, DMSO) δ 11.42 (s, 1H), 9.87 (s, 1H), 8.22 (s, 1H), 7.40 (dd, *J* = 7.5, 2.0 Hz, 1H), 7.34 – 7.22 (m, 2H), 6.08 (s, 1H), 3.90 (s, 2H), 3.72 (s, 1H), 3.69 – 3.58 (m, 4H), 3.28 – 3.19 (m, 3H), 3.02 (s, 2H), 2.40 (s, 3H), 2.33 (s, 1H), 2.24 (s, 3H), 1.84 (s, 2H), 1.77 (d, *J* = 12.9 Hz, 3H), 1.40 (s, 9H).

MS: obs. m/z [M+H$^+$]: 642.5.

N-(2-chloro-6-methylphenyl)-2-((2-methyl-6-(4-(piperidin-4-yloxy)piperidin-1-yl)pyrimidin-4-yl)amino)thiazole-5-carboxamide

tert-butyl 4-((1-(6-((5-((2-chloro-6-methylphenyl)carbamoyl)thiazol-2-yl)amino)-2-methylpyrimidin-4-yl)piperidin-4-yl)oxy)piperidine-1-carboxylate (111.2 mg, 173.1 µmol, 1.0 Eq) was suspended in dichloromethane (1.5 mL) at room temperature and a solution of HCl in 1,4-dioxane (303 µL, 4 M, 1.212 mmol, 7.0 Eq) was added. The reaction mixture was stirred at room temperature for 4.5 hours. The mixture was concentrated under reduced pressure, yielding the title compound N-(2-chloro-6-methylphenyl)-2-((2-methyl-6-(4-(piperidin-4-yloxy)piperidin-1-yl)pyrimidin-4-yl)amino)thiazole-5-carboxamide as a light orange oil (94 mg, 173 µmol, 100 %), which was used for the next step without further purification.

^{1}H NMR (400 MHz, DMSO) δ 10.10 (s, 1H), 8.96 (s, 2H), 8.35 (d, *J* = 7.5 Hz, 1H), 7.40 (dd, *J* = 7.4, 2.0 Hz, 1H), 7.32 – 7.21 (m, 2H), 3.91 (s, 2H), 3.80 – 3.72 (m, 2H), 3.73 – 3.65 (m, 1H), 3.40 (dd, *J* = 10.9, 6.6 Hz, 2H), 3.14 (s, 2H), 2.94 (s, 2H), 2.54 (s, 3H), 2.24 (s, 3H), 1.99 – 1.86 (m, 4H), 1.73 – 1.63 (m, 2H), 1.51 (d, *J* = 8.9 Hz, 2H).

MS: obs. m/z [M+H$^+$]: 542.3.

Ethyl 8-(4-((1-(6-((5-((2-chloro-6-methylphenyl)carbamoyl)thiazol-2-yl)amino)-2-methylpyrimidin-4-yl)piperidin-4-yl)oxy)piperidin-1-yl)octanoate

Ethyl 8-bromooctanoate (139.3 mg, 554.5 µmol, 3.0 Eq) was added to a solution of N-(2-chloro-6-methylphenyl)-2-((2-methyl-6-(4-(piperidin-4-yloxy)piperidin-1-yl)pyrimidin-4-yl)amino)thiazole-5-carboxamide (100.2 mg, 184.8 µmol, 1.0 Eq) and N,N-diisopropylethylamine (161 µL, 924.2 µmol, 5.0 Eq) in DMF (1 mL) and the reaction mixture was heated at 80 °C for 24 hours. The mixture was purified by silica gel column chromatography, eluted with methanol (from 0 % to 20 %) in dichloromethane, yielding the title compound ethyl 8-(4-((1-(6-((5-((2-chloro-6-methylphenyl)carbamoyl)thiazol-2-yl)amino)-2-methylpyrimidin-4-yl)piperidin-4-yl)oxy)piperidin-1-yl)octanoate as a dark orange solid (100 mg, 140 mmol, 80 %).

^{1}H NMR (400 MHz, DMSO-D$_6$) δ ppm: 11.21 (s, 2H), 7.29 (s, 1H), 7.20 – 7.12 (m, 1H), 4.10 (q, J = 7.1 Hz, 1H), 3.65 (pd, J = 6.7, 4.0 Hz, 6H), 3.31 (d, J = 11.5 Hz, 1H), 3.08 (dt, J = 7.4, 3.7 Hz, 5H), 2.88 (q, J = 5.6 Hz, 1H), 2.51 (s, 3H), 2.35 (s, 2H), 2.27 (t, J = 7.5 Hz, 1H), 1.87 (s, 4H), 1.59 (d, J = 6.4 Hz, 2H), 1.56 (s, 2H), 1.54 (s, 3H), 1.51 (s, 4H), 1.48 (d, J = 6.5 Hz, 1H), 1.44 (s, 4H), 1.32 (d, J = 4.8 Hz, 4H), 1.24 (t, J = 7.1 Hz, 2H).

MS: obs. m/z [M+H$^+$]: 712.7.

8-(4-((1-(6-((5-((2-Chloro-6-methylphenyl)carbamoyl)thiazol-2-yl)amino)-2-methylpyrimidin-4-yl)piperidin-4-yl)oxy)piperidin-1-yl)octanoic acid

Ethyl 8-(4-((1-(6-((5-((2-chloro-6-methylphenyl)carbamoyl)thiazol-2-yl)amino)-2-methylpyrimidin-4-yl)piperidin-4-yl)oxy)piperidin-1-yl)octanoate (100.0 mg, 140.4 µmol, 1.0 Eq) was dissolved in a mixture of THF (800.0 µL) and water (533.3 µL), lithium hydroxide (6.7 mg, 280.8 µmol, 2.0 Eq) was added in one portion and the reaction mixture was stirred at room temperature for 3 hours. The mixture was concentrated under reduced pressure, yielding the title compound 8-(4-((1-(6-((5-((2-Chloro-6-methylphenyl)carbamoyl)thiazol-2-yl)amino)-2-methylpyrimidin-4-yl)piperidin-4-yl)oxy)piperidin-1-yl)octanoic acid as a beige solid (89 mg, 130 µmol, 93 %), which used for the next step without further purification.

MS: obs. m/z [M+H$^+$]: 684.3.

Tert-butyl (2-((7-(4-(8-(4-((1-(6-((5-((2-chloro-6-methylphenyl)carbamoyl)thiazol-2-yl)amino)-2-methylpyrimidin-4-yl)piperidin-4-yl)oxy)piperidin-1-yl)octanoyl)piperazin-1-yl)-2-(1-(4-chlorophenyl)cyclohexyl)quinazolin-4-yl)amino)ethyl)carbamate

To a mixture of 8-(4-((1-(6-((5-((2-chloro-6-methylphenyl)carbamoyl)thiazol-2-yl)amino)-2-methylpyrimidin-4-yl)piperidin-4-yl)oxy)piperidin-1-yl)octanoic acid (44.38 mg, 64.85 µmol, 1.0 Eq), HATU (49.32 mg, 129.7 µmol, 2.0 Eq) and N,N-diisopropylethylamine (45.2 µL, 259.4 µmol, 4.0 Eq) in DMF (500.0 µL) was added tert-butyl (2-((2-(1-(4-chlorophenyl)cyclohexyl)-7-(piperazin-1-yl)quinazolin-4-yl)amino)ethyl)carbamate* (36.65 mg, 64.85 µmol, 1.0 Eq) and the reaction mixture was stirred at 25 °C for 5 hours. The mixture was directly purified by preparative HPLC on a Waters XBridge C18 OBD column (50 x 30 mm, 5 µm) eluting with acetonitrile (from 65 % to 95 %) in an aqueous solution of NH_4OH (5.0 mM). Fractions containing the title compound were combined and freeze dried, yielding the title compound tert-butyl (2-((7-(4-(8-(4-((1-(6-((5-((2-chloro-6-methylphenyl)carbamoyl)thiazol-2-yl)amino)-2-methylpyrimidin-4-yl)piperidin-4-yl)oxy)piperidin-1-yl)octanoyl)piperazin-1-yl)-2-(1-(4-chlorophenyl)cyclohexyl)quinazolin-4-yl)amino)ethyl)carbamate as a white solid (16.70 mg, 13 µmol, 20 %).

* synthesis described in [2]

^{1}H NMR (400 MHz, DMSO) δ 11.41 (s, 1H), 9.86 (s, 1H), 8.21 (s, 1H), 8.02 – 7.82 (m, 2H), 7.47 (d, *J* = 8.4 Hz, 2H), 7.40 (dd, *J* = 7.6, 2.0 Hz, 1H), 7.33 – 7.17 (m, 5H), 6.91 (t, *J* = 5.7 Hz, 1H), 6.85 (d, *J* = 2.4 Hz, 1H), 6.07 (s, 1H), 3.90 (d, *J* = 12.9 Hz, 2H), 3.65 (s, 1H), 3.59 (d, *J* = 5.5 Hz, 4H), 3.46 (d, *J* = 6.2 Hz, 2H), 3.41 (d, *J* = 9.2 Hz, 2H), 3.29 (s, 4H), 3.26 – 3.12 (m, 5H), 2.80 (d, *J* = 12.8 Hz, 2H), 2.66 (d, *J* = 10.2 Hz, 2H), 2.40 (s, 3H), 2.34 (t, *J* = 7.4 Hz, 3H), 2.24 (s, 3H), 2.20 (t, *J* = 7.3 Hz, 2H), 2.03 – 1.88 (m, 4H), 1.77 (d, *J* = 15.6 Hz, 4H), 1.50 (dd, *J* = 15.2, 8.5 Hz, 8H), 1.37 (s, 9H), 1.27 (s, 6H), 1.23 (s, 2H), 1.17 (s, 1H).

MS: obs. m/z [$M+H^+$]: 1231.2.

2-((6-(4-((1-(8-(4-(4-((2-Aminoethyl)amino)-2-(1-(4-chlorophenyl)cyclohexyl)quinazolin-7-yl)piperazin-1-yl)-8-oxooctyl)piperidin-4-yl)oxy)piperidin-1-yl)-2-methylpyrimidin-4-yl)amino)-N-(2-chloro-6-methylphenyl)thiazole-5-carboxamide

To a solution of tert-butyl (2-((7-(4-(8-(4-((1-(6-((5-((2-chloro-6-methylphenyl)carbamoyl)thiazol-2-yl)amino)-2-methylpyrimidin-4-yl)piperidin-4-yl)oxy)piperidin-1-yl)octanoyl)piperazin-1-yl)-2-(1-(4-chlorophenyl)cyclohexyl)quinazolin-4-yl)amino)ethyl)carbamate (14.80 mg, 12.02 µmol, 1.0 Eq) in dichloromethane (100 µL) was added a solution of HCl in 1,4-dioxane (15.02 µL, 4 M, 60.09 µmol, 5.0 Eq) and the reaction mixture was stirred at room temperature for 4 hours. The mixture was concentrated under reduced pressure, yielding the title compound 2-((6-(4-((1-(8-(4-(4-((2-aminoethyl)amino)-2-(1-(4-chlorophenyl)cyclohexyl)quinazolin-7-yl)piperazin-1-yl)-8-oxooctyl)piperidin-4-yl)oxy)piperidin-1-yl)-2-methylpyrimidin-4-yl)amino)-N-(2-chloro-6-methylphenyl)thiazole-5-carboxamide as a white solid (14.3 mg, 11 µmol, 92 %), which was used for the next step without further purification.

^{1}H NMR (400 MHz, DMSO) δ 13.39 (d, *J* = 5.2 Hz, 1H), 10.75 (s, 1H), 10.26 (s, 1H), 10.13 (s, 1H), 8.47 (d, *J* = 9.4 Hz, 1H), 8.37 (s, 3H), 7.67 (d, *J* = 4.0 Hz, 1H), 7.57 (dd, *J* = 8.7, 1.6 Hz, 2H), 7.41 – 7.36 (m, 4H), 7.31 – 7.22 (m, 2H), 3.94 (dd, *J* = 37.3, 11.0 Hz, 4H), 3.76 – 3.60 (m, 5H), 3.50 (ddd, *J* = 4.9, 4.1, 1.4 Hz, 3H), 3.46 (td, *J* = 4.5, 1.4 Hz, 5H), 3.26 (d, *J* = 11.7 Hz, 1H), 3.16 (d, *J* = 6.1 Hz, 2H), 2.96 (q, *J* = 12.9 Hz, 5H), 2.54 (s, 3H), 2.35 (t, *J* = 7.4 Hz, 2H), 2.24 (s, 3H), 2.12 (d, *J* = 6.0 Hz, 2H), 2.03 (s, 2H), 1.89 (s, 3H), 1.74 – 1.59 (m, 4H), 1.46 (d, *J* = 40.0 Hz, 7H), 1.32 (d, *J* = 17.9 Hz, 8H), 0.92 – 0.82 (m, 1H).

^{13}C NMR (150 MHz, DMSO-D_6) δ ppm: 170.97, 165.56, 162.50, 159.10, 156.34, 154.24, 154.00, 142.80, 141.20, 138.76, 137.29, 133.34, 132.41, 131.77, 131.72, 131.62, 129.06, 128.88, 128.66, 128.53, 128.29, 128.23, 127.93, 127.04, 125.99, 125.01, 116.05, 102.11, 98.65, 72.17, 70.53, 70.17, 69.61, 60.18, 55.57, 55.08, 49.94, 48.96, 47.04, 46.21, 43.96, 43.64, 37.87, 34.13, 32.18, 30.71, 29.79, 28.88, 28.53, 28.36, 27.08, 26.08, 24.93, 24.55, 23.25, 23.01, 22.66, 18.34, 13.91, 10.82.

MS: obs. m/z [M+H$^+$]: 1130.8.

Compound DBt-5 Synthetic Methods:

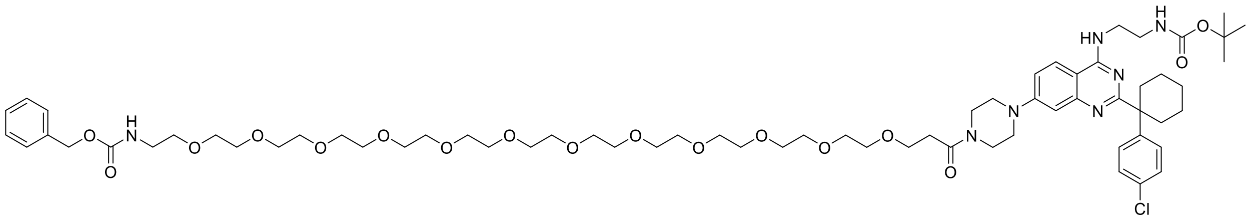

Tert-butyl (2-((2-(1-(4-chlorophenyl)cyclohexyl)-7-(4-(3-oxo-1-phenyl-2,7,10,13,16,19,22,25,28,31,34,37,40-tridecaoxa-4-azatritetracontan-43-oyl)piperazin-1-yl)quinazolin-4-yl)amino)ethyl)carbamate

To a solution of 3-oxo-1-phenyl-2,7,10,13,16,19,22,25,28,31,34,37,40-tridecaoxa-4-azatritetracontan-43-oic acid* (80 mg, 0.105 mmol, 1.0 Eq) in DMA (1 ml) was added N,N-diisopropyl ethylamine (0.055 ml, 0.315 mmol, 3.0 Eq) and HATU (52.5 mg, 0.137 mmol, 1.3 Eq) and the reaction mixture was stirred at room temperature for 20 minutes. Tert-butyl (2-((2-(1-(4-chlorophenyl)cyclohexyl)-7-(piperazin-1-yl)quinazolin-4-yl)amino)ethyl)carbamate (60 mg, 0.105 mmol, 1.0 Eq) was added and stirring was continued for 1 hour. The mixture was partitioned between water (15 mL) and ethyl acetate (15 mL), the aqueous phase was extracted with ethyl acetate (2 x 15 mL), the combined organic phases were washed with water (10 mL) and brine (10 mL) and dried over Na_2SO_4. The residue was purified by silica gel column chromatography, eluted with a mixture (9:1) of ethyl acetate/methanol (from 10 % to 90 %) in ethyl acetate, yielding the title compound tert-butyl (2-((2-(1-(4-chlorophenyl)cyclohexyl)-7-(4-(3-oxo-1-phenyl-2,7,10,13,16,19,22,25,28,31,34,37,40-tridecaoxa-4-azatritetracontan-43-oyl)piperazin-1-yl)quinazolin-4-yl)amino)ethyl)carbamate as a pale yellow oil (130 mg, 0.100 mmol, 95 %).

*CAS: 1334177-88-6 is commercially available.

MS: obs. m/z [M+H$^+$]: 1298.8.

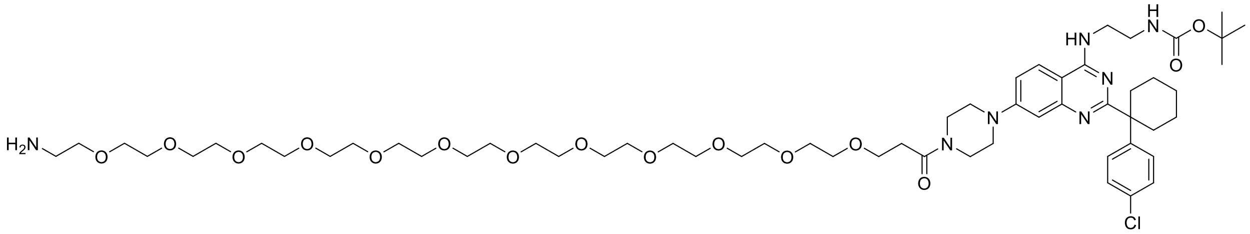

tert-butyl (2-((7-(4-(1-amino-3,6,9,12,15,18,21,24,27,30,33,36-dodecaoxanonatriacontan-39-oyl)piperazin-1-yl)-2-(1-(4-chlorophenyl)cyclohexyl)quinazolin-4-yl)amino)ethyl)carbamate

To a solution of tert-butyl (2-((2-(1-(4-chlorophenyl)cyclohexyl)-7-(4-(3-oxo-1-phenyl-2,7,10,13,16,19,22,25,28,31,34,37,40-tridecaoxa-4-azatritetracontan-43-oyl)piperazin-1-yl)quinazolin-4-

yl)amino)ethyl)carbamate (110 mg, 0.080 mmol, 1.0 Eq) in ethanol (5 mL) under an argon atmosphere was added chlorobenzene (16 µL, 0.161 mmol, 2.0 Eq), a solution of HCl in ethanol (0.071 mL, 0.088 mmol, 1.1 Eq) and palladium on carbon (17.12 mg, 0.016 mmol, 0.2 Eq). The argon atmosphere was replaced by hydrogen and the reaction mixture was stirred at room temperature for 1.5 hours. The mixture was filtered through a pad of Celite, the solids were washed with ethanol (4 x 10 mL) and the combined filtrates were concentrated under reduced pressure. The residue was purified by preparative HPLC on a Waters SUNFIRE C18 OBD column (30 x 100 mm, 5 µm) eluting with acetonitrile (from 5 % to 70 %) in an aqueous solution of TFA (0.1 %). Fractions containing the title compound were combined and the pH was adjusted to 10 using a saturated, aqueous solution of $NaHCO_3$. After the evaporation of the majority of the acetonitrile the mixture was partitioned between water (15 mL) and ethyl acetate (15 mL). The aqueous layer was extracted with ethyl acetate (2 x 15mL), the combined organic phases were washed with brine (10 mL) and dried over Na_2SO_4, yielding the target compound tert-butyl (2-((7-(4-(1-amino-3,6,9,12,15,18,21,24,27,30,33,36-dodecaoxanonatriacontan-39-oyl)piperazin-1-yl)-2-(1-(4-chlorophenyl)cyclohexyl)quinazolin-4-yl)amino)ethyl)carbamate as a colorless resin (55 mg, 0.047 mmol, 59 %).

MS: obs. m/z [M+H$^+$]: 1164.6.

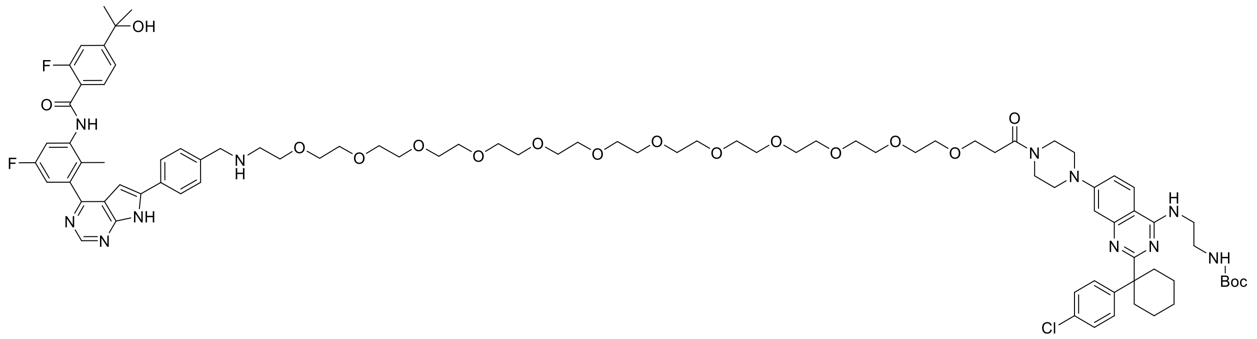

Tert-butyl (2-((2-(1-(4-chlorophenyl)cyclohexyl)-7-(4-(1-(4-(4-(5-fluoro-3-(2-fluoro-4-(2-hydroxypropan-2-yl)benzamido)-2-methylphenyl)-7H-pyrrolo[2,3-d]pyrimidin-6-yl)phenyl)-5,8,11,14,17,20,23,26,29,32,35,38-dodecaoxa-2-azahentetracontan-41-oyl)piperazin-1-yl)quinazolin-4-yl)amino)ethyl)carbamate

To a suspension of 2-fluoro-N-(5-fluoro-3-(6-(4-formylphenyl)-7H-pyrrolo[2,3-d]pyrimidin-4-yl)-2-methylphenyl)-4-(2-hydroxypropan-2-yl)benzamide* (11.63 mg, 0.022 mmol, 1.0 Eq) in a mixture (1:1) of methanol (1 mL) and THF (1 mL) was added tert-butyl (2-((7-(4-(1-amino-3,6,9,12,15,18,21,24,27,30,33,36-dodecaoxanonatriacontan-39-oyl)piperazin-1-yl)-2-(1-(4-chlorophenyl)cyclohexyl)quinazolin-4-yl)amino)ethyl)carbamate (26 mg, 0.022 mmol, 1.0 Eq), Na_2SO_4 (34 mg, 0.239 mmol, 11 Eq) and acetic acid (1.3 µl, 0.022 mmol, 1.0 Eq) and the reaction mixture was stirred at room temperature for 20 minutes. Sodium cyanoborohydride (7.3 mg, 0.055 mmol, 2.5 Eq) was added and stirring was continued for 1 hour. Additional sodium cyanoborohydride (7.3 mg, 0.055 mmol, 2.5 Eq) and THF (0.5 mL) were added and stirring was continued for 20 hours. MeOH (1 mL) was added and after stirring for additional 30 minutes the solvents were removed. The residue was purified by preparative HPLC on a Waters SUNFIRE C18 OBD column (30 x 100 mm, 5 µm.) eluting with acetonitrile (from 5 % to

70 %) in an aqueous solution of TFA (0.1 %), yielding the title compound tert-butyl (2-((2-(1-(4-chlorophenyl)cyclohexyl)-7-(4-(1-(4-(4-(5-fluoro-3-(2-fluoro-4-(2-hydroxypropan-2-yl)benzamido)-2-methylphenyl)-7H-pyrrolo[2,3-d]pyrimidin-6-yl)phenyl)-5,8,11,14,17,20,23,26,29,32,35,38-dodecaoxa-2-azahentetracontan-41-oyl)piperazin-1-yl)quinazolin-4-yl)amino)ethyl)carbamate as a white solid (12 mg, 0.007 mmol, 33 %).

* synthesis described in patent WO2021/55295, page 195.

MS: obs. m/z [M+2H$^+$/2]: 838.2.

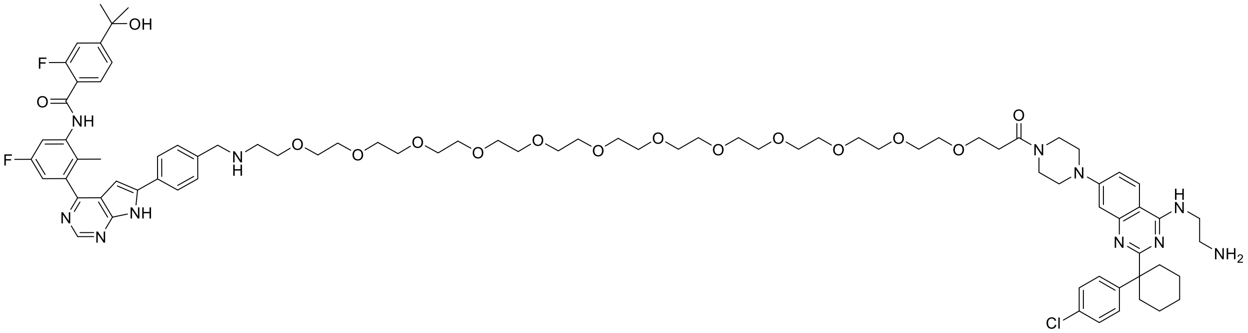

DBt-5

N-(3-(6-(4-(41-(4-(4-((2-aminoethyl)amino)-2-(1-(4-chlorophenyl)cyclohexyl)quinazolin-7-yl)piperazin-1-yl)-41-oxo-5,8,11,14,17,20,23,26,29,32,35,38-dodecaoxa-2-azahentetracontyl)phenyl)-7H-pyrrolo[2,3-d]pyrimidin-4-yl)-5-fluoro-2-methylphenyl)-2-fluoro-4-(2-hydroxypropan-2-yl)benzamide

To a solution of tert-butyl (2-((2-(1-(4-chlorophenyl)cyclohexyl)-7-(4-(1-(4-(4-(5-fluoro-3-(2-fluoro-4-(2-hydroxypropan-2-yl)benzamido)-2-methylphenyl)-7H-pyrrolo[2,3-d]pyrimidin-6-yl)phenyl)-5,8,11,14,17,20,23,26,29,32,35,38-dodecaoxa-2-azahentetracontan-41-oyl)piperazin-1-yl)quinazolin-4-yl)amino)ethyl)carbamate (11 mg, 5.11 µmol, 1.0 Eq) in dichloromethane (50 µL) at 0 °C was added TFA (75 µl, 0.968 mmol, 190 Eq) and the reaction mixture was stirred at room temperature for 1.5 hours. Water (10 mL) was added, the aqueous phase was extracted with ethyl acetate (3 x 10 mL), the combined organic phases were washed with brine (2 x 5 mL) and dried over sodium sulfate. The residue was purified by preparative HPLC on a Waters SUNFIRE C18 OBD column (30 x 100 mm, 5 µm) eluting with acetonitrile (from 5 % to 70 %) in an aqueous solution of TFA (0.1 %). Fractions containing the final compound were combined and freeze-dried, yielding the title compound N-(3-(6-(4-(41-(4-(4-((2-aminoethyl)amino)-2-(1-(4-chlorophenyl)cyclohexyl)quinazolin-7-yl)piperazin-1-yl)-41-oxo-5,8,11,14,17,20,23,26,29,32,35,38-dodecaoxa-2-azahentetracontyl)phenyl)-7H-pyrrolo[2,3-d]pyrimidin-4-yl)-5-fluoro-2-methylphenyl)-2-fluoro-4-(2-hydroxypropan-2-yl)benzamide as a yellow solid (6 mg, 3.81 µmol, 75 %).

^{1}H NMR (600 MHz, DMSO-D$_6$) δ ppm: 1.43 – 1.32 (m, 1H), 1.45 (s, 6H), 1.45-1.48 (m, 2H), 1.57 – 1.50 (m, 1H), 1.67 – 1.58 (m, 2H), 2.16 – 2.04 (m, 2H), 2.18 (s, 3H), 2.63 (t, J = 6.7 Hz, 2H), 2.81 – 2.71 (m, 2H), 3.20 – 3.07 (m, 4H), 3.55 – 3.41 (m, 44H), 3.60 – 3.55 (m, 4H), 3.62 – 3.60 (m, 2H), 3.67 – 3.62 (m, 4H), 3.70 (t, J = 5.2 Hz, 2H), 4.00 – 3.85 (m, 2H), 4.22 (t, J = 5.6 Hz, 2H), 6.96 (d, J = 2.0 Hz, 1H), 7.13 (s, 1H), 7.24 (dd, J = 8.8, 2.8 Hz, 1H), 7.46 – 7.37 (m, 5H), 7.48 (d, J = 8.8 Hz, 2H), 7.60 (d, J = 8.4 Hz, 2H), 7.67 (d, J = 10.1 Hz,

1H), 7.73 (t, J = 7.8 Hz, 1H), 8.10 – 7.99 (m, 5H), 8.13 (d, J = 9.1 Hz, 1H), 8.88 (s, 1H), 9.10 – 8.92 (m, 2H), 9.82 (s br, 1H), 9.96 (d, J = 2.5 Hz, 1H), 12.78 (s br, 1H), 12.87 (s, 1H).

^{13}C NMR (150 MHz, DMSO-D_6) δ ppm: 169.15, 162.86, 159.42 (d, J = 250 Hz), 159.28, 159.10 (d, J = 248 Hz), 158.24, 158.03, 156.83, 156.78, 154.36, 153.31, 150.97, 142.84, 139.42 (d, J = 8.6 Hz), 139.17, 138.43 (d, J = 10.5 Hz), 132.24, 131.14, 130.68, 129.89 (d, J = 2.9 Hz), 128.55, 128.37, 126.48 (d, J = 3.1 Hz), 125.96, 125.39, 121.33 (d, J = 14.6 Hz), 120.79 (d, J = 2.9 Hz), 117.94, 116.29, 113.41 (d, J = 22.1 Hz), 112.54 (d, J = 24.5 Hz), 112.35 (d, J = 23.3 Hz), 97.92, 97.12, 70.60 (d, J = 1.4 Hz), 69.73, 69.67, 69.56, 66.70, 65.62, 49.74, 48.93, 46.12, 45.83, 44.00, 37.92, 34.08, 32.80, 31.56, 24.86, 22.57, 14.57.

HR MS: obs. m/z [M+H$^+$]: calculated for $C_{83}H_{111}N_{11}O_{15}ClF_2$, 1574.79112; found, 1574.79100; deviation: 0.1 ppm.

Compound DBt-10 Synthetic Methods:

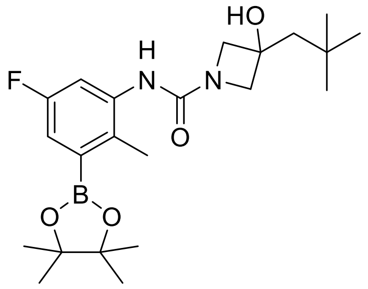

N-(5-fluoro-2-methyl-3-(4,4,5,5-tetramethyl-1,3,2-dioxaborolan-2-yl)phenyl)-3-hydroxy-3-neopentylazetidine-1-carboxamide

To a solution of 5-fluoro-2-methyl-3-(4,4,5,5-tetramethyl-1,3,2-dioxaborolan-2-yl)aniline* (3.6 g, 14.34 mmol, 1.0 Eq) and N,N-diisopropyl ethylamine (10.02 mL, 57.3 mmol, 4.0 Eq) in dichloromethane (40 mL) at 0 °C was added a solution of phosgene in toluene (9.05 mL, 20 %, 17.20 mmol, 1.2 Eq) over 10 minutes and the reaction mixture was stirred at 0 °C for 10 minutes. This mixture was canulated into a stirred solution of 3-neopentylazetidin-3-ol** (2.26 g, 15.77 mmol, 1.1 Eq) in dichloromethane (40.0 mL) at 0 °C and the resulting reaction mixture was stirred at 0 °C for 1 hour. The solvents were removed under reduced pressure, the residue was triturated in a mixture (1:5) of dichloromethane/TBME, filtered and the solids were dried under vacuum. The filtrate was concentrated, crystallized in TBME and all the solids were combined, yielding the title compound N-(5-fluoro-2-methyl-3-(4,4,5,5-tetramethyl-1,3,2-dioxaborolan-2-yl)phenyl)-3-hydroxy-3-neopentylazetidine-1-carboxamide as a white solid (5 g, 11.90 mmol, 83 %).

* CAS: 1418128-33-2 is commercially available.

** synthesis described in patent WO2021/053495, pages 352-353.

1H NMR: (400 MHz, DMSO-d6) δ ppm: 0.98 (s, 9H), 1.30 (s, 12H), 1.65 (s, 2H), 2.31 (s, 3H), 3.79 (d, J = 8.5 Hz, 2H), 3.90 (d, J = 8.5 Hz, 2H), 5.47 (s, 1H), 7.07 (dd, J = 8.7, 2.9 Hz, 1H), 7.32 (dd, J = 10.6, 2.9 Hz, 1H), 7.74 (s, 1H).

MS: obs. m/z [M+H$^+$]: 421.

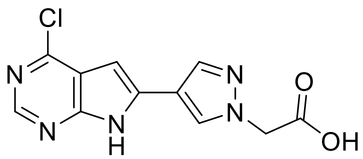

2-(4-(4-Chloro-7H-pyrrolo[2,3-d]pyrimidin-6-yl)-1H-pyrazol-1-yl)acetic acid

To a solution of 4-chloro-6-iodo-7H-pyrrolo[2,3-d]pyrimidine* (1.50 g, 5.37 mmol, 1.0 Eq) and ethyl 2-(4-(4,4,5,5-tetramethyl-1,3,2-dioxaborolan-2-yl)-1H-pyrazol-1-yl)acetate** (1.80 g, 6.43 mmol, 1.2 Eq) in a mixture (1:1) of 1,4-dioxane (25 ml) and water (25 mL) was added cesium carbonate (4.36 g, 13.42 mmol, 2.5 Eq) and $PdCl_2(PPh_2)$ferrocene DCM complex (0.438 g, 0.537 mmol, 0.1 Eq) and reaction mixture was stirred at 100 °C for 1 hour. Ethyl acetate (80 mL) was added, the aqueous phase was separated, the pH was adjusted to 2-3 by addition of an aqueous solution of HCl (0.1 M) and the aqueous phase was extracted with ethyl acetate (3 x 20 mL). The combined organic phases were washed with water (30 mL) and brine (30 mL) and dried over sodium sulfate. The aqueous phase was extracted with n-butanol (3 x 30 mL) and all organic phases were combined, yielding the title compound 2-(4-(4-chloro-7H-pyrrolo[2,3-d]pyrimidin-6-yl)-1H-pyrazol-1-yl)acetic acid as a slightly brown solid (1.49 g, 5.37 mmol, 100 %).

* CAS: 876343-10-1 is commercially available.

** CAS: 864754-16-5 is commercially available.

MS: obs. m/z $[M+H^+]$: 278.1.

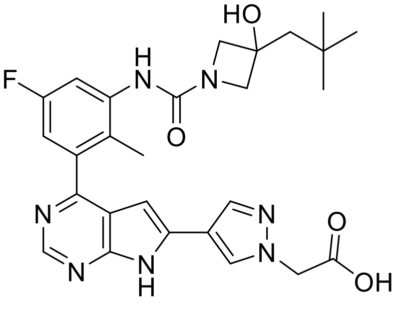

2-(4-(4-(5-Fluoro-3-(3-hydroxy-3-neopentylazetidine-1-carboxamido)-2-methylphenyl)-7H-pyrrolo[2,3-d]pyrimidin-6-yl)-1H-pyrazol-1-yl)acetic acid

To a solution of 2-(4-(4-chloro-7H-pyrrolo[2,3-d]pyrimidin-6-yl)-1H-pyrazol-1-yl)acetic acid (0.792 g, 2.86 mmol, 1.2 Eq) in a mixture of acetonitrile (25 mL) and water (15 mL) was added N-(5-fluoro-2-methyl-3-(4,4,5,5-tetramethyl-1,3,2-dioxaborolan-2-yl)phenyl)-3-hydroxy-3-neopentylazetidine-1-carboxamide (1.0 g, 2.38 mmol, 1.0 Eq), potassium carbonate (8.31 ml, 8.31 mmol, 3.6 Eq) and $Pd(dppf)Cl_2$ (0.253 g, 0.346 mmol, 0.15 Eq) and the reaction mixture was stirred at 100 °C for 30 minutes. Ethyl acetate (40 mL) and water (40 mL) were added and the aqueous phase was separated. The pH of the aqueous phase was adjusted to 2-3 by addition of an aqueous solution of HCl (0.1 M), extracted with ethyl acetate (3 x 25 mL), the combined organic phases were washed with brine (50 mL) and dried over sodium sulfate, yielding the title compound 2-(4-(4-(5-fluoro-3-(3-hydroxy-3-neopentylazetidine-1-carboxamido)-2-methylphenyl)-7H-pyrrolo[2,3-d]pyrimidin-6-yl)-1H-pyrazol-1-yl)acetic acid as a beige solid (1.112 g, 2.08 mmol, 87 %).

MS: obs. m/z [M+H$^+$]: 536.3.

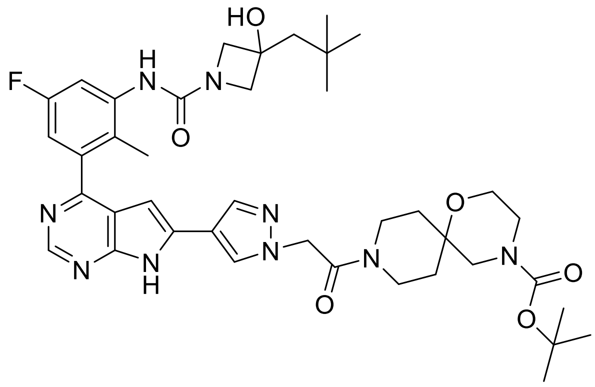

Tert-butyl 9-(2-(4-(4-(5-fluoro-3-(3-hydroxy-3-neopentylazetidine-1-carboxamido)-2-methylphenyl)-7H-pyrrolo[2,3-d]pyrimidin-6-yl)-1H-pyrazol-1-yl)acetyl)-1-oxa-4,9-diazaspiro[5.5]undecane-4-carboxylate

To a solution of 2-(4-(4-(5-fluoro-3-(3-hydroxy-3-neopentylazetidine-1-carboxamido)-2-methylphenyl)-7H-pyrrolo[2,3-d]pyrimidin-6-yl)-1H-pyrazol-1-yl)acetic acid (300 mg, 0.56 mmol, 1.0 Eq) in DMA (3 mL) was added HATU (237 mg, 0.605 mmol, 1.2 Eq) and N,N-diisopropyl ethylamine (0.308 ml, 1.764 mmol, 3.5 Eq) and the reaction mixture was stirred at room temperature for 15 minutes. A solution of tert-butyl 1-oxa-4,9-diazaspiro[5.5]undecane-4-carboxylate* (166 mg, 0.648 mmol 1.2 Eq) in DMA (2 mL) was added and stirring was continued for 30 minutes. Additional HATU (119 mg, 0.302 mmol, 0.6 Eq) and N,N-diisopropyl ethylamine (0.154 ml, 0.882 mmol, 1.75 Eq) were added and the reaction mixture was stirred for additional 30 minutes. Ethyl acetate (50 mL) and water (50 mL) were added, the aqueous phase was extracted with ethyl acetate (2 x 25 mL) and the combined organic phases were washed with brine (20 mL) and dried over sodium sulfate. The residue was purified by silica gel column chromatography, eluted with a mixture (9:1) of dichloromethane/methanol (from 10 % to 90 %) in dichloromethane, yielding the title compound tert-butyl 9-(2-(4-(4-(5-fluoro-3-(3-hydroxy-3-neopentylazetidine-1-carboxamido)-2-methylphenyl)-7H-pyrrolo[2,3-d]pyrimidin-6-yl)-1H-pyrazol-1-yl)acetyl)-1-oxa-4,9-diazaspiro[5.5]undecane-4-carboxylate as a beige solid (200 mg, 0.258 mmol, 46 %).

* CAS: 1023595-11-0 is commercially available.

MS: obs. m/z [M+H$^+$]: 774.5.

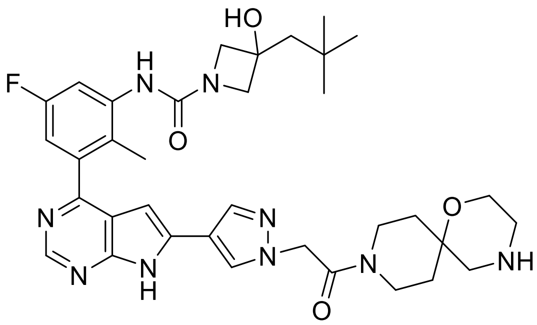

N-(5-fluoro-2-methyl-3-(6-(1-(2-oxo-2-(1-oxa-4,9-diazaspiro[5.5]undecan-9-yl)ethyl)-1H-pyrazol-4-yl)-7H-pyrrolo[2,3-d]pyrimidin-4-yl)phenyl)-3-hydroxy-3-neopentylazetidine-1-carboxamide

To a solution of tert-butyl 9-(2-(4-(4-(5-fluoro-3-(3-hydroxy-3-neopentylazetidine-1-carboxamido)-2-methylphenyl)-7H-pyrrolo[2,3-d]pyrimidin-6-yl)-1H-pyrazol-1-yl)acetyl)-1-oxa-4,9-diazaspiro[5.5]undecane-4-carboxylate (200 mg, 0.258 mmol, 1.0 Eq) in DCM (1 mL) was added TFA (0.594 ml, 7.75 mmol, 30 Eq) and the reaction mixture was stirred at room temperature for 60 minutes. The mixture was evaporated to dryness, yielding the title compound N-(5-fluoro-2-methyl-3-(6-(1-(2-oxo-2-(1-oxa-4,9-diazaspiro[5.5]undecan-9-yl)ethyl)-1H-pyrazol-4-yl)-7H-pyrrolo[2,3-d]pyrimidin-4-yl)phenyl)-3-hydroxy-3-neopentylazetidine-1-carboxamide as a beige solid (174 mg, 0.258 mmol, 99 %), which was used for the next step without further purification.

MS: obs. m/z [M+H$^+$]: 674.5.

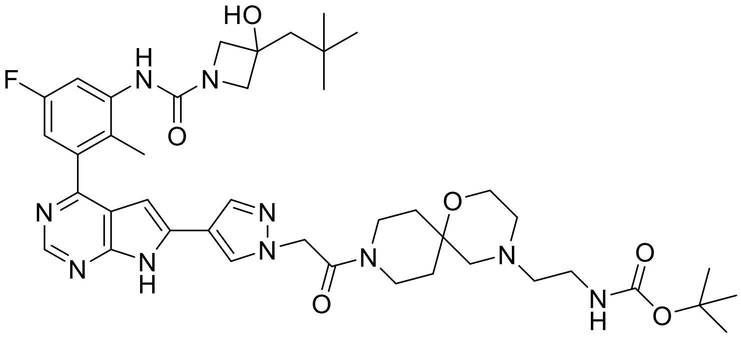

Tert-butyl (2-(9-(2-(4-(4-(5-fluoro-3-(3-hydroxy-3-neopentylazetidine-1-carboxamido)-2-methylphenyl)-7H-pyrrolo[2,3-d]pyrimidin-6-yl)-1H-pyrazol-1-yl)acetyl)-1-oxa-4,9-diazaspiro[5.5]undecan-4-yl)ethyl)carbamate

To a solution of N-(5-fluoro-2-methyl-3-(6-(1-(2-oxo-2-(1-oxa-4,9-diazaspiro[5.5]undecan-9-yl)ethyl)-1H-pyrazol-4-yl)-7H-pyrrolo[2,3-d]pyrimidin-4-yl)phenyl)-3-hydroxy-3-neopentylazetidine-1-carboxamide (174 mg, 0.258 mmol, 1.0 Eq) in a mixture (10:1) of methanol (1.8 ml) and acetic acid (0.2 mL) was added N-Boc-2-aminoacetaldehyde (51.9 mg, 0.326 mmol, 1.3 Eq) and the reaction mixture was stirred at room temperature for 1 hour. Sodium cyanoborohydride (24.32 mg, 0.387 mmol, 1.5 Eq) was added and stirring was continued for 16 hours. Ethyl acetate (50 mL) and water (50 mL) were added, the aqueous phase was extracted with ethyl acetate (2 x 25 mL) and the combined organic phases were washed with

brine (20 mL) and dried over sodium sulfate, yielding the title compound tert-butyl (2-(9-(2-(4-(4-(5-fluoro-3-(3-hydroxy-3-neopentylazetidine-1-carboxamido)-2-methylphenyl)-7H-pyrrolo[2,3-d]pyrimidin-6-yl)-1H-pyrazol-1-yl)acetyl)-1-oxa-4,9-diazaspiro[5.5]undecan-4-yl)ethyl)carbamate as a beige solid (114 mg, 0.139 mmol, 54 %), which was used for the next step without further purification.

MS: obs. m/z [M+H$^+$]: 817.5.

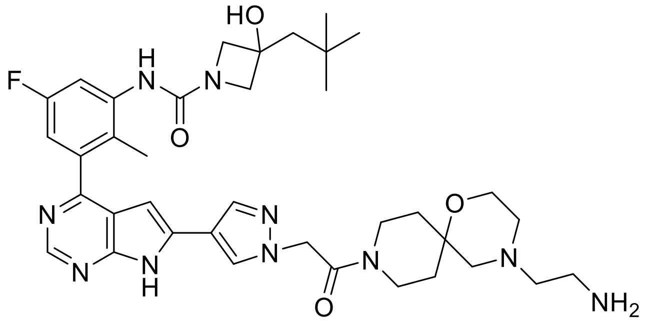

N-(3-(6-(1-(2-(4-(2-aminoethyl)-1-oxa-4,9-diazaspiro[5.5]undecan-9-yl)-2-oxoethyl)-1H-pyrazol-4-yl)-7H-pyrrolo[2,3-d]pyrimidin-4-yl)-5-fluoro-2-methylphenyl)-3-hydroxy-3-neopentylazetidine-1-carboxamide

To a solution of tert-butyl (2-(9-(2-(4-(4-(5-fluoro-3-(3-hydroxy-3-neopentylazetidine-1-carboxamido)-2-methylphenyl)-7H-pyrrolo[2,3-d]pyrimidin-6-yl)-1H-pyrazol-1-yl)acetyl)-1-oxa-4,9-diazaspiro[5.5]undecan-4-yl)ethyl)carbamate (246 mg, 0.301 mmol, 1.0 Eq) in DCM (1 mL) was added TFA (0.692 ml, 9.03 mmol, 30 Eq) and the reaction mixture was stirred at room temperature for 30 minutes. Butanol (50 mL) was added and the mixture was washed with water (2 x 10 mL). The organic phase was washed with brine (20 mL) and dried over sodium sulfate, yielding the title compound N-(3-(6-(1-(2-(4-(2-aminoethyl)-1-oxa-4,9-diazaspiro[5.5]undecan-9-yl)-2-oxoethyl)-1H-pyrazol-4-yl)-7H-pyrrolo[2,3-d]pyrimidin-4-yl)-5-fluoro-2-methylphenyl)-3-hydroxy-3-neopentylazetidine-1-carboxamide as a beige solid (204 mg, 0.285 mmol, 95 %), which was used for the next step without further purification.

^{1}H NMR (400 MHz, DMSO-D$_6$) δ ppm: 0.98 (s, 9H), 1.20 – 1.34 (m, 2H), 1.35 – 1.45 (m, 2H), 1.50 – 1.59 (m, 1H), 1.85 – 1.98 (m, 2H), 2.07 (s, 3H), 2.18 – 2.37 (m, 2H), 2.39 – 2.45 (m, 2H), 2.88 – 2.97 (m, 2H), 2.97 – 3.05 (m, 1H), 3.58 – 3.71 (m, 3H), 3.83 (d, J = 8.6 Hz, 2H), 3.87 – 3.98 (m, 3H), 5.15 – 5.26 (m, 2H), 5.51 (s, 1H), 6.49 (d, J = 1.5 Hz, 1H), 7.04 (dd, J = 8.9, 2.8 Hz, 1H), 7.43 (dd, J = 10.8, 2.8 Hz, 1H), 7.57 (s br, 2H), 7.92 (s, 1H), 8.04 (s, 1H), 8.20 (s, 1H), 8.76 (s, 1H), 12.58 (s, 1H).

MS: obs. m/z [M+H$^+$]: 717.6.

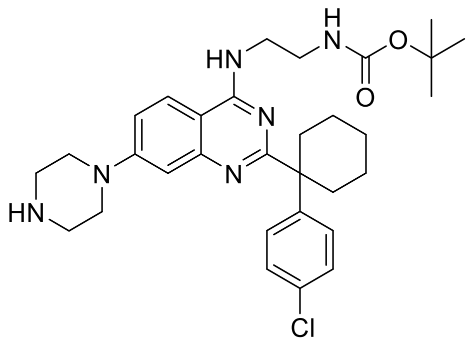

Tert-butyl (2-((2-(1-(4-chlorophenyl)cyclohexyl)-7-(piperazin-1-yl)quinazolin-4-yl)amino)ethyl)carbamate

To a solution of tert-butyl (2-((7-(4-acetylpiperazin-1-yl)-2-(1-(4-chlorophenyl)cyclohexyl)quinazolin-4-yl)amino)ethyl)carbamate (4.074 g, 6.71 mmol, 1.0 Eq) in ethanol (10 mL) was added an aqueous solution of potassium hydroxide (27.8 ml, 2 M, 55.7 mmol, 10 Eq) and the reaction mixture was stirred at 80 °C for 48 hours. Ethyl acetate (80 mL) was added, the organic phase was washed with water (2 x 25 mL) and brine (1 x 25 mL) and dried over sodium sulfate. The residue was purified by silica gel column chromatography, eluted with a mixture (9:1) of dichloromethane/methanol (from 10 % to 90 %) in dichloromethane, yielding the title compound tert-butyl (2-((2-(1-(4-chlorophenyl)cyclohexyl)-7-(piperazin-1-yl)quinazolin-4-yl)amino)ethyl)carbamate as a slightly brown powder (1.78 g, 3.15 mmol, 47 %).

MS: obs. m/z [M+H$^+$]: 565.3.

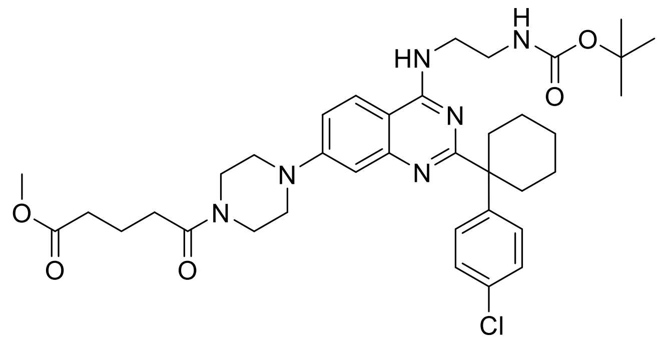

Methyl 5-(4-(4-((2-((tert-butoxycarbonyl)amino)ethyl)amino)-2-(1-(4-chlorophenyl)cyclohexyl)quinazolin-7-yl)piperazin-1-yl)-5-oxopentanoate

To a solution of tert-butyl (2-((2-(1-(4-chlorophenyl)cyclohexyl)-7-(piperazin-1-yl)quinazolin-4-yl)amino)ethyl)carbamate (200 mg, 0.354 mmol, 1.0 Eq) and triethylamine (0.075 ml, 0.531 mmol, 1.5 Eq) in DCM (2 mL) was added methyl 5-chloro-5-oxopentanoate (0.054 ml, 0.389 mmol, 1.1 Eq) and the reaction mixture was stirred at room temperature for 1 hour. Ethyl acetate (50 mL) was added, the organic phase was washed with water (2 x 25 mL) and brine (25 mL) and dried over sodium sulfate, yielding the title compound methyl 5-(4-(4-((2-((tert-butoxycarbonyl)amino)ethyl)amino)-2-(1-(4-chlorophenyl)cyclohexyl)quinazolin-7-yl)piperazin-1-yl)-5-oxopentanoate as a beige solid (240 mg, 0.346 mmol, 98 %), which was used for the next step without further purification.

MS: obs. m/z [M+H⁺]: 693.0.

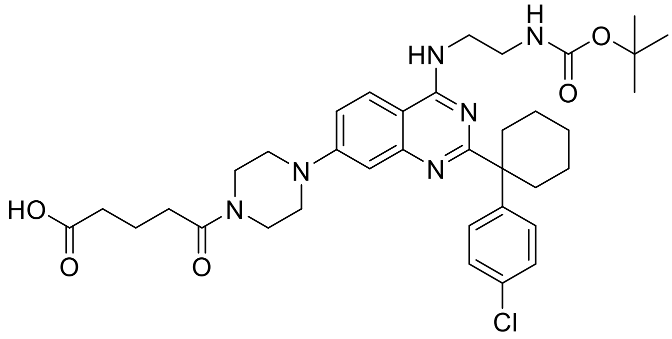

5-(4-(4-((2-((Tert-butoxycarbonyl)amino)ethyl)amino)-2-(1-(4-chlorophenyl)cyclohexyl)quinazolin-7-yl)piperazin-1-yl)-5-oxopentanoic acid

To a solution of methyl 5-(4-(4-((2-((tert-butoxycarbonyl)amino)ethyl)amino)-2-(1-(4-chlorophenyl)cyclohexyl)quinazolin-7-yl)piperazin-1-yl)-5-oxopentanoate (240 mg, 0.346 mmol, 1.0 Eq) in THF (10 ml) was added an aqueous solution of sodium hydroxide (0.247 ml, 2 M, 0.493 mmol, 1.5 Eq) and the reaction mixture was stirred at room temperature for 6 hours. Ethyl acetate (50 mL) was added and the organic phase was washed with water (3 x 20 mL). The pH of the aqueous phase was adjusted to pH 2-3 by the addition of an aqueous solution of HCl (0.1 M) and then extracted with n-butanol (4 x 15 mL). The combined organic phases were washed with brine and dried over sodium sulfate, yielding the title compound 5-(4-(4-((2-((tert-butoxycarbonyl)amino)ethyl)amino)-2-(1-(4-chlorophenyl)cyclohexyl)quinazolin-7-yl)piperazin-1-yl)-5-oxopentanoic acid as a beige solid (108 mg, 0.159 mmol, 46 %), which was used for the next step without further purification.

MS: obs. m/z [M+H⁺]: 679.4.

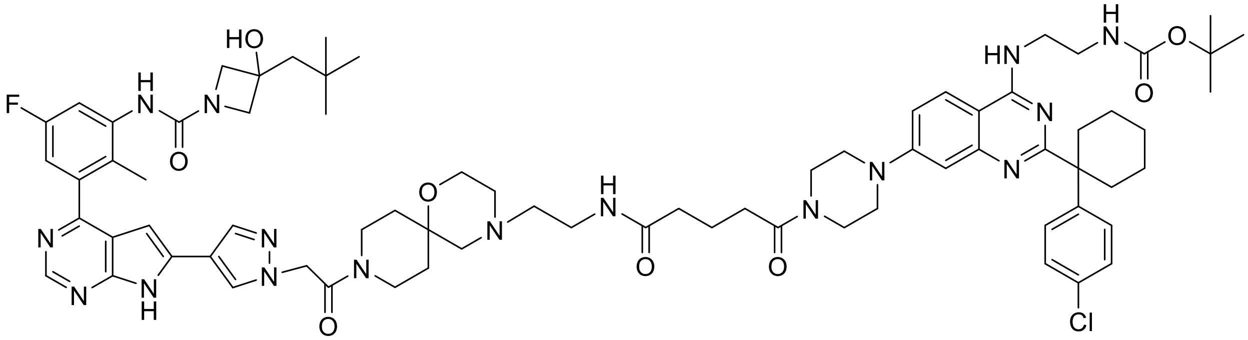

Tert-butyl (2-((2-(1-(4-chlorophenyl)cyclohexyl)-7-(4-(5-((2-(9-(2-(4-(4-(5-fluoro-3-(3-hydroxy-3-neopentylazetidine-1-carboxamido)-2-methylphenyl)-7H-pyrrolo[2,3-d]pyrimidin-6-yl)-1H-pyrazol-1-yl)acetyl)-1-oxa-4,9-diazaspiro[5.5]undecan-4-yl)ethyl)amino)-5-oxopentanoyl)piperazin-1-yl)quinazolin-4-yl)amino)ethyl)carbamate

To a solution of N-(3-(6-(1-(2-(4-(2-aminoethyl)-1-oxa-4,9-diazaspiro[5.5]undecan-9-yl)-2-oxoethyl)-1H-pyrazol-4-yl)-7H-pyrrolo[2,3-d]pyrimidin-4-yl)-5-fluoro-2-methylphenyl)-3-hydroxy-3-neopentylazetidine-1-carboxamide (80 mg, 0.112 mmol, 1.0 Eq) in DMA (2 mL) was added HATU (66.5 mg, 0.170 mmol, 1.5 Eq) and N,N-diisopropyl ethylamine (0.079 ml, 0.452 mmol. 4.0 Eq) and the reaction mixture was stirred at room temperature for 15 minutes A solution of 5-(4-(4-((2-((tert-butoxycarbonyl)amino)ethyl)amino)-2-(1-(4-chlorophenyl)cyclohexyl)quinazolin-7-yl)piperazin-1-yl)-5-oxopentanoic acid (81 mg, 0.119 mmol, 1.0 Eq) in DMA (1 mL) was added and stirring was continued for 1 hour. Additional HATU (33 mg, 0.085 mmol, 0.75 Eq) and N,N-diisopropyl ethylamine (0.040 ml, 0.225 mmol. 2.0 Eq) were added and stirring was continued for 16 hours. Ethyl acetate (50 mL) was added, the organic phase was washed with water (2 x 10 mL) and brine (10 mL) and dried over sodium sulfate. The residue was purified by preparative HPLC on a Waters SUNFIRE C18 OBD column (30 x 100 mm, 5 µm) eluting with acetonitrile (from 5 % to 85 %) in an aqueous solution of TFA (0.1 %). Fractions containing the title compound were combined, acetonitrile was partially removed under reduced pressure, a saturated, aqueous solution of $NaHCO_3$ was added to adjust the pH to 10 and the aqueous phase was extracted with ethyl acetate. The combined organic phases were dried over sodium sulfate, yielding the title compound tert-butyl (2-((2-(1-(4-chlorophenyl)cyclohexyl)-7-(4-(5-((2-(9-(2-(4-(4-(5-fluoro-3-(3-hydroxy-3-neopentylazetidine-1-carboxamido)-2-methylphenyl)-7H-pyrrolo[2,3-d]pyrimidin-6-yl)-1H-pyrazol-1-yl)acetyl)-1-oxa-4,9-diazaspiro[5.5]undecan-4-yl)ethyl)amino)-5-oxopentanoyl)piperazin-1-yl)quinazolin-4-yl)amino)ethyl)carbamate as a slightly yellow solid (92 mg, 0.067 mmol, 60 %).

MS: obs. m/z [M+H$^+$]: 1377.6.

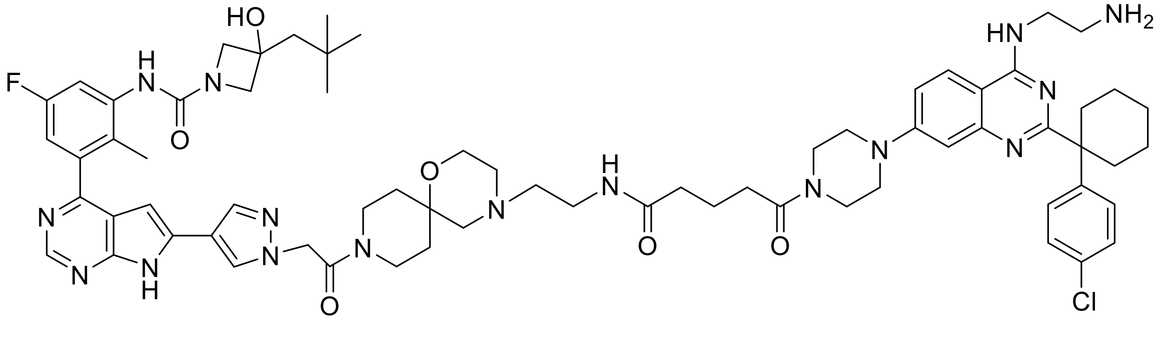

DBt-10

N-(3-(6-(1-(2-(4-(2-(5-(4-(4-((2-aminoethyl)amino)-2-(1-(4-chlorophenyl)cyclohexyl)quinazolin-7-yl)piperazin-1-yl)-5-oxopentanamido)ethyl)-1-oxa-4,9-diazaspiro[5.5]undecan-9-yl)-2-oxoethyl)-1H-pyrazol-4-yl)-7H-pyrrolo[2,3-d]pyrimidin-4-yl)-5-fluoro-2-methylphenyl)-3-hydroxy-3-neopentylazetidine-1-carboxamide

To a solution of tert-butyl (2-((2-(1-(4-chlorophenyl)cyclohexyl)-7-(4-(5-((2-(9-(2-(4-(4-(5-fluoro-3-(3-hydroxy-3-neopentylazetidine-1-carboxamido)-2-methylphenyl)-7H-pyrrolo[2,3-d]pyrimidin-6-yl)-1H-pyrazol-1-yl)acetyl)-1-oxa-4,9-diazaspiro[5.5]undecan-4-yl)ethyl)amino)-5-oxopentanoyl)piperazin-1-yl)quinazolin-4-yl)amino)ethyl)carbamate (92 mg, 0.067 mmol, 1.0 Eq) in DCM (1 mL) was added TFA (0.256 ml, 3.34 mmol, 50 Eq) and the reaction mixture was stirred at room temperature for 1 hour. The mixture was evaporated to dryness and the residue was purified by preparative HPLC on a Waters SUNFIRE C18 OBD column (30 x 100 mm, 5 µm) eluting with acetonitrile (from 5 % to 70 %) in an aqueous solution of TFA (0.1 %). Fractions containing the title compound were combined, acetonitrile was partially removed under reduced pressure, a saturated, aqueous solution of $NaHCO_3$ was added to adjust the pH to 10 and the aqueous phase was extracted with ethyl acetate. The combined organic phases were dried over sodium sulfate, yielding the title compound N-(3-(6-(1-(2-(4-(2-(5-(4-(4-((2-aminoethyl)amino)-2-(1-(4-chlorophenyl)cyclohexyl)quinazolin-7-yl)piperazin-1-yl)-5-oxopentanamido)ethyl)-1-oxa-4,9-diazaspiro[5.5]undecan-9-yl)-2-oxoethyl)-1H-pyrazol-4-yl)-7H-pyrrolo[2,3-d]pyrimidin-4-yl)-5-fluoro-2-methylphenyl)-3-hydroxy-3-neopentylazetidine-1-carboxamide as a slightly yellow solid (58 mg, 0.045mmol, 68 %).

^{1}H NMR (600 MHz, DMSO-D_6) δ ppm: 0.98 (s, 9H), 1.34 - 1.57 (m, 4H), 1.59 – 1.65 (m, 2H), 1.66 (s, 2H), 1.76 (p, J = 7.5 Hz, 2H), 2.07 (s, 3H), 2.09 – 2.15 (m, 2H), 2.18 (t, J = 7.5 Hz, 2H), 2.38 (t, J = 7.3 Hz, 2H), 2.70 – 2.80 (m, 2H), 2.83 – 3.22 (m, 10H), 3.32 – 3.67 (m, 12H), 3.68 – 3.77 (m, 2H), 3.79 – 4.05 (m, 10H), 5.19 (d, J = 16.1 Hz, 1H), 5.26 (d, J = 16.1 Hz, 1H), 6.53 (s, 1H), 7.05 (dd, J = 8.8, 2.9 Hz, 1H), 7.14 (s br, 1H), 7.37 – 7.42 (m, 2H), 7.42 – 7.50 (m, 4H), 7.92 (s, 1H), 8.01 – 8.10 (m, 4H), 8.13 (d, J = 9.3 Hz, 1H), 8.20 (s br, 1H), 8.22 (s, 1H), 8.81 (s, 1H), 9.81 (s, 1H), 12.72 (s, 1H), 12.80 (s, 1H).

^{13}C NMR (150 MHz, DMSO-D_6) δ ppm: 173.09, 170.63, 165.69, 164.98, 159.35 (d, J = 240.6 Hz), 156.76, 154.65, 154.36, 152.79, 149.34, 142.85, 140.91, 139.86 (d, J = 11.0 Hz), 138.58, 137.12, 134.30, 131.83, 129.85, 128.56, 128.38, 125.57, 125.39, 118.15, 116.28, 113.67, 111.74 (d, J = 24 Hz), 111.55 (d, J = 22 Hz), 101.98, 97.96, 94.21, 69.90, 69.44, 64.27, 57.04, 56.47, 52.97, 51.27, 50.18, 48.92, 46.08, 45.84, 43.84, 39.38, 39.24, 37.92, 36.73, 34.51, 34.06, 33.24, 31.65, 31.28, 30.54, 24.85, 22.56, 20.60, 14.56.

HR MS: obs. m/z [M+H$^+$]: calculated for $C_{68}H_{87}N_{16}O_6ClF$, 1277.66616; found, 1277.66550; deviation: 0.5 ppm.

NMR spectra

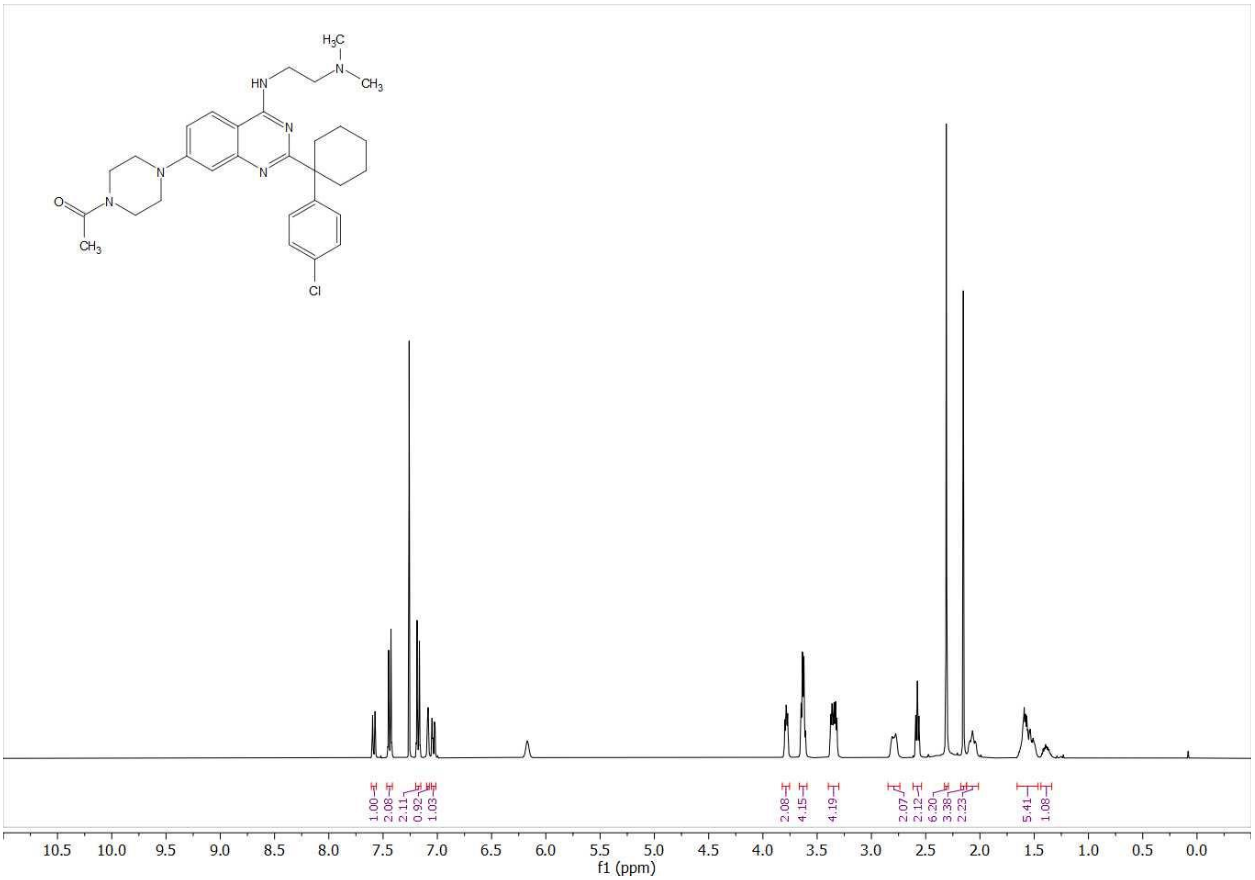

Supplementary spectrum 1: ^{1}H-NMR spectrum of **13-N**

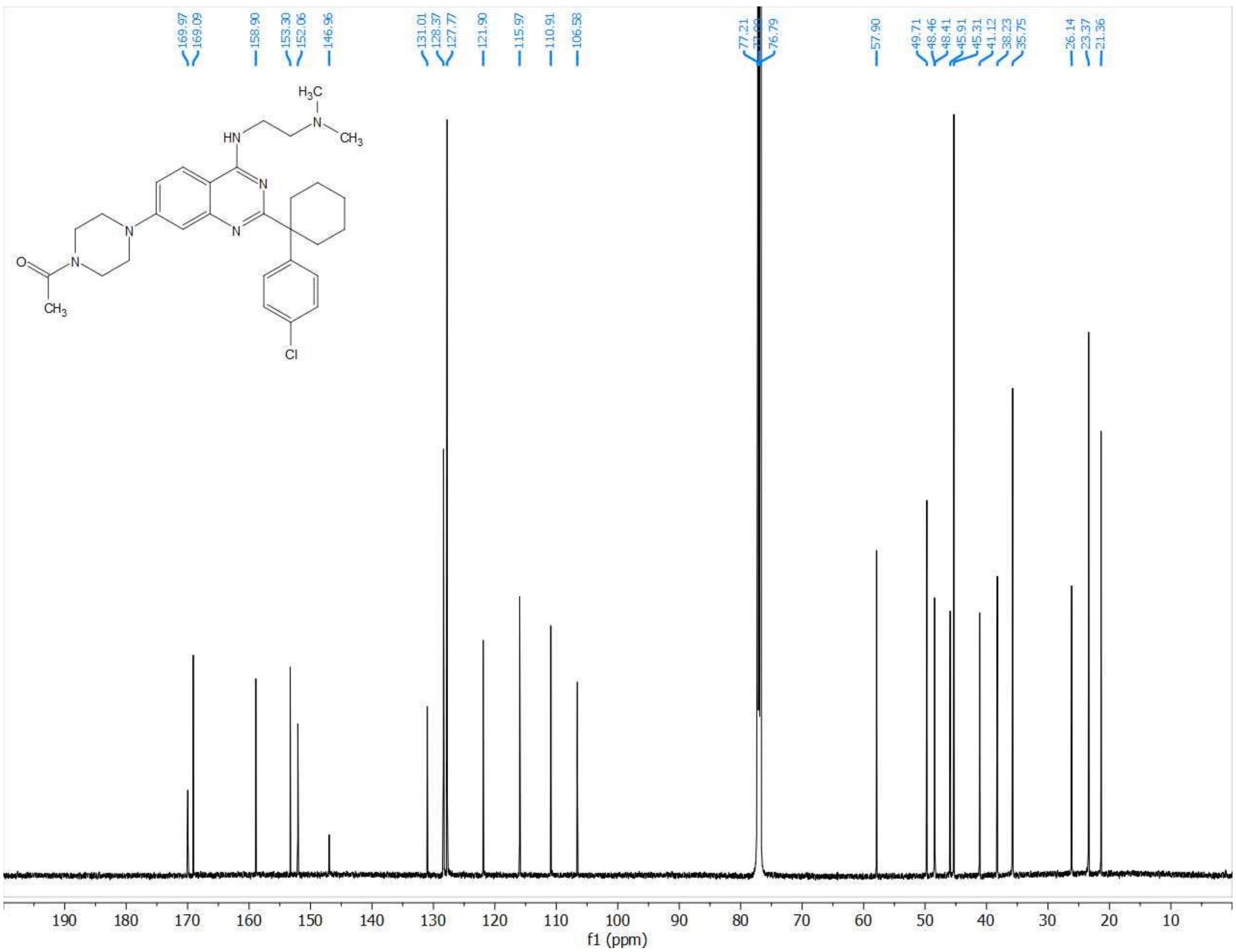

Supplementary spectrum 2: ^{13}C-NMR spectrum of **13-N** .

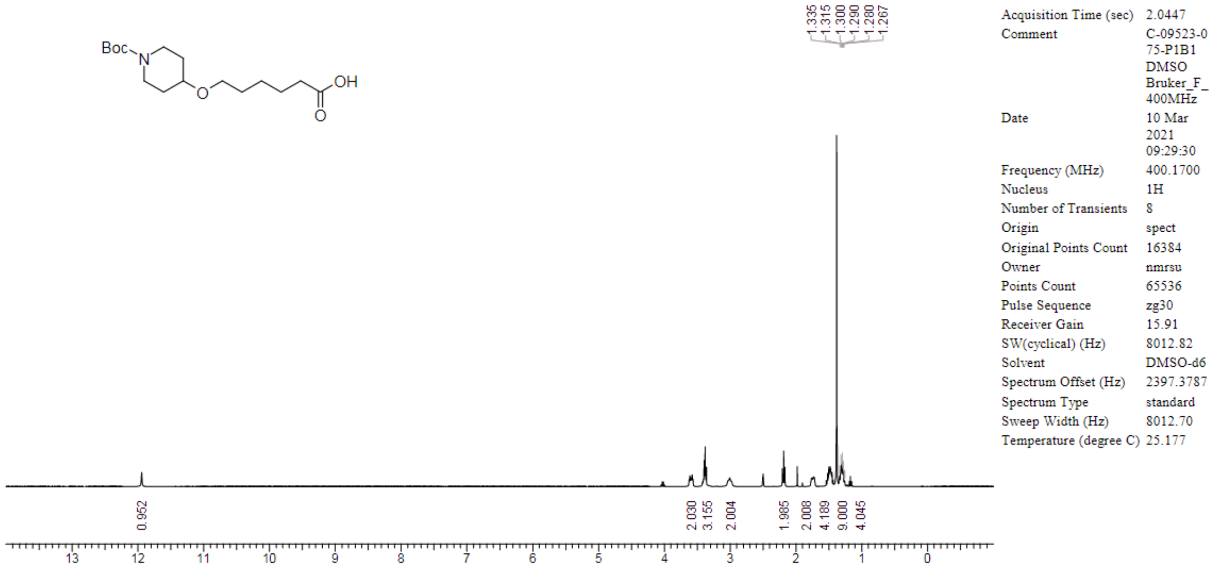

Supplementary spectrum 3: ^{1}H-NMR spectrum of **6-((1-(Tert-butoxycarbonyl)piperidin-4-yl)oxy)hexanoic acid**

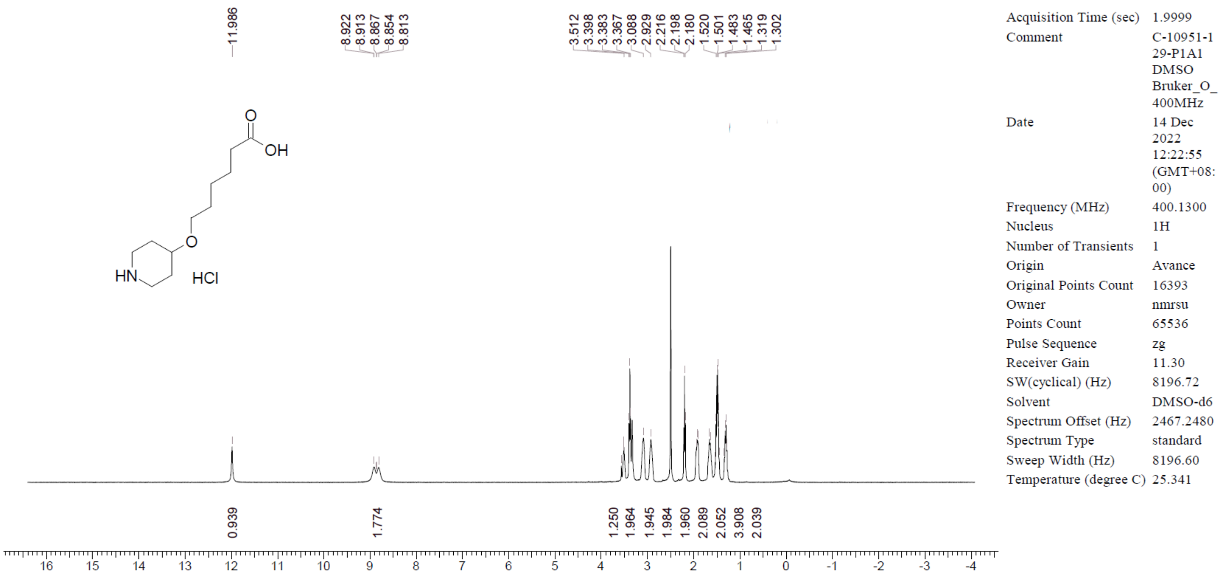

Supplementary spectrum 4: ^{1}H-NMR spectrum of **6-(Piperidin-4-yloxy)hexanoic acid**

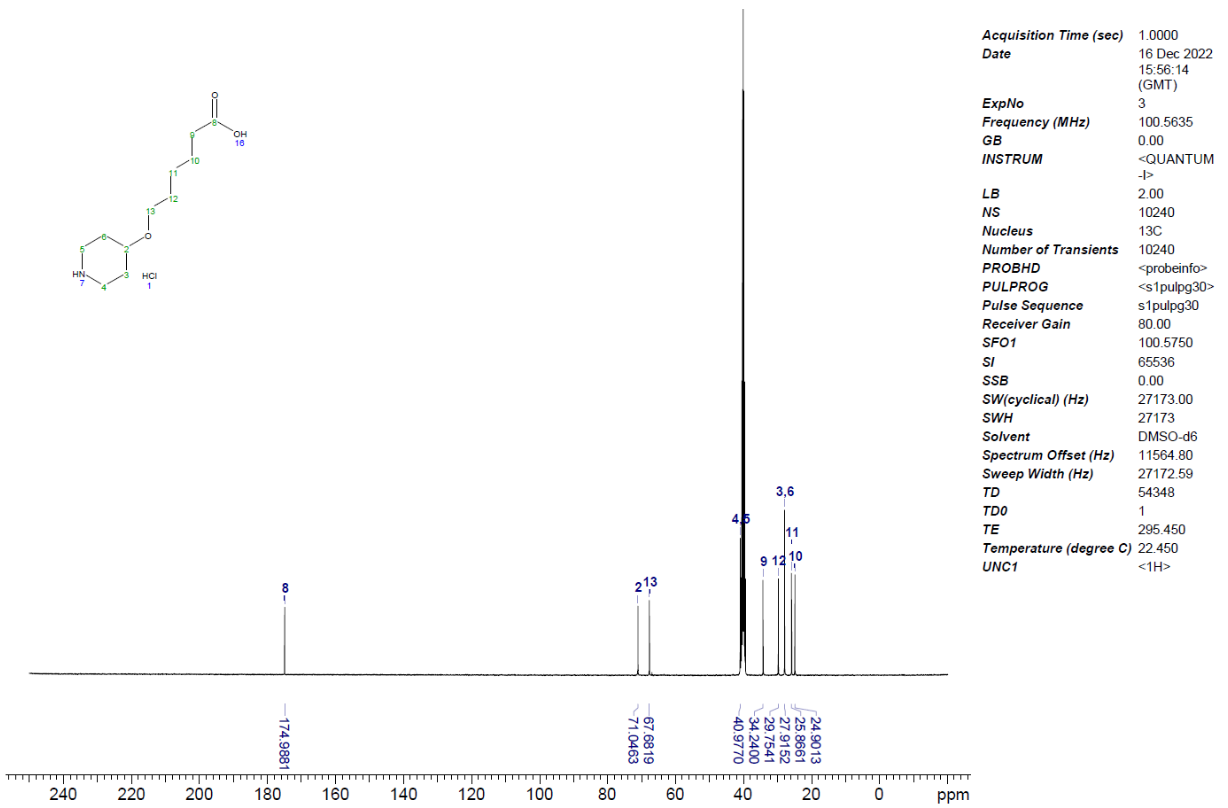

Supplementary spectrum 5: ^{13}C-NMR spectrum of **6-(Piperidin-4-yloxy)hexanoic acid**

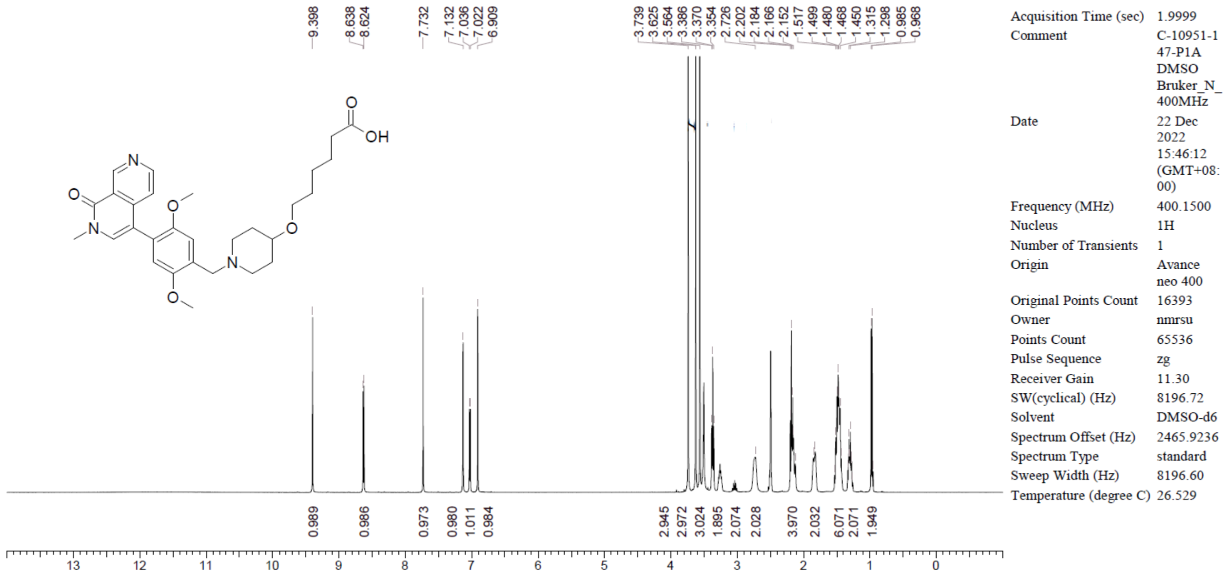

Supplementary spectrum 6: ^{1}H-NMR spectrum of **6-((1-(2,5-Dimethoxy-4-(2-methyl-1-oxo-1,2-dihydro-2,7-naphthyridin-4-yl)benzyl)piperidin-4-yl)oxy)hexanoic acid**

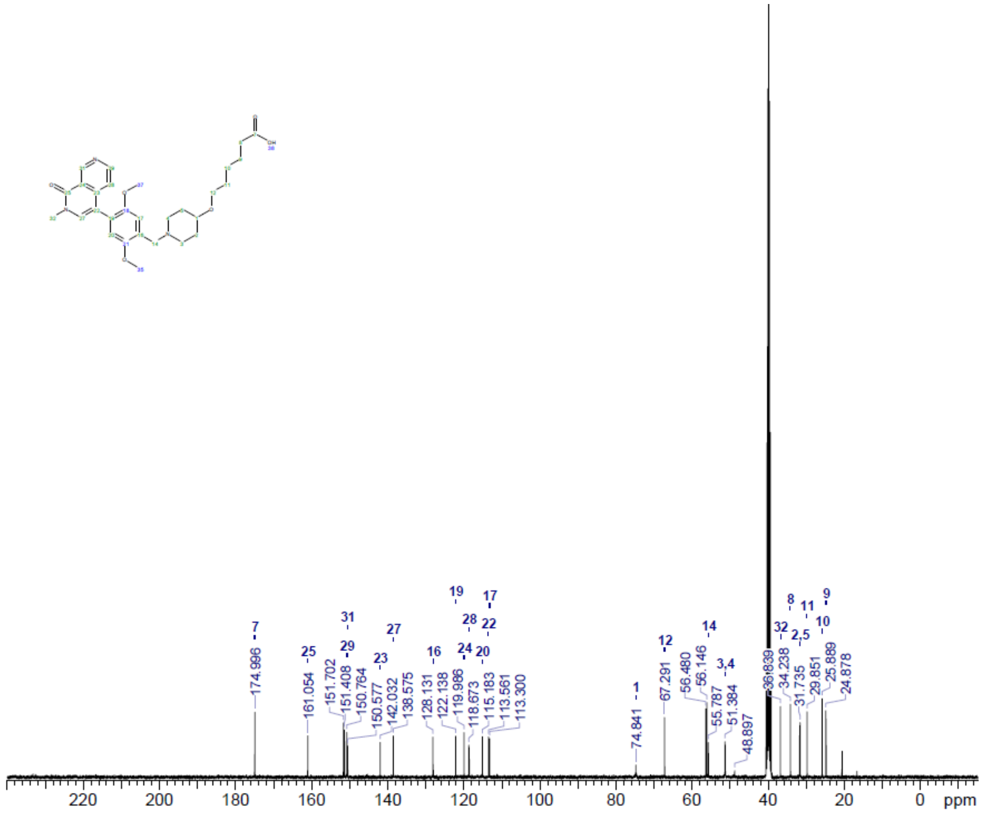

Acquisition Time (sec)	1.2190
Date	24 Dec 2022 08:03:53 (GMT+08:00)
ExpNo	4
Frequency (MHz)	100.6178
GB	0.00
INSTRUM	<Avance neo 400>
LB	1.00
NS	1024
Nucleus	13C
Number of Transients	1024
PROBHD	<Z163739_0597 (PI HR-BBO400 S1-BBF/H/D -5.0-Z SP)>
PULPROG	<zgpg30>
Pulse Sequence	zgpg30
Receiver Gain	101.00
SFO1	100.6294
SI	32768
SSB	0.00
SW(cyclical) (Hz)	26881.72
SWH	26881.72043 01075
Solvent	DMSO-d6
Spectrum Offset (Hz)	11571.04
Sweep Width (Hz)	26880.90
TD	65536
TD0	1
TE	296.880
Temperature (degree C)	23.880
UNC1	<13C>

Supplementary spectrum 7: ^{13}C-NMR spectrum of **6-((1-(2,5-Dimethoxy-4-(2-methyl-1-oxo-1,2-dihydro-2,7-naphthyridin-4-yl)benzyl)piperidin-4-yl)oxy)hexanoic acid**

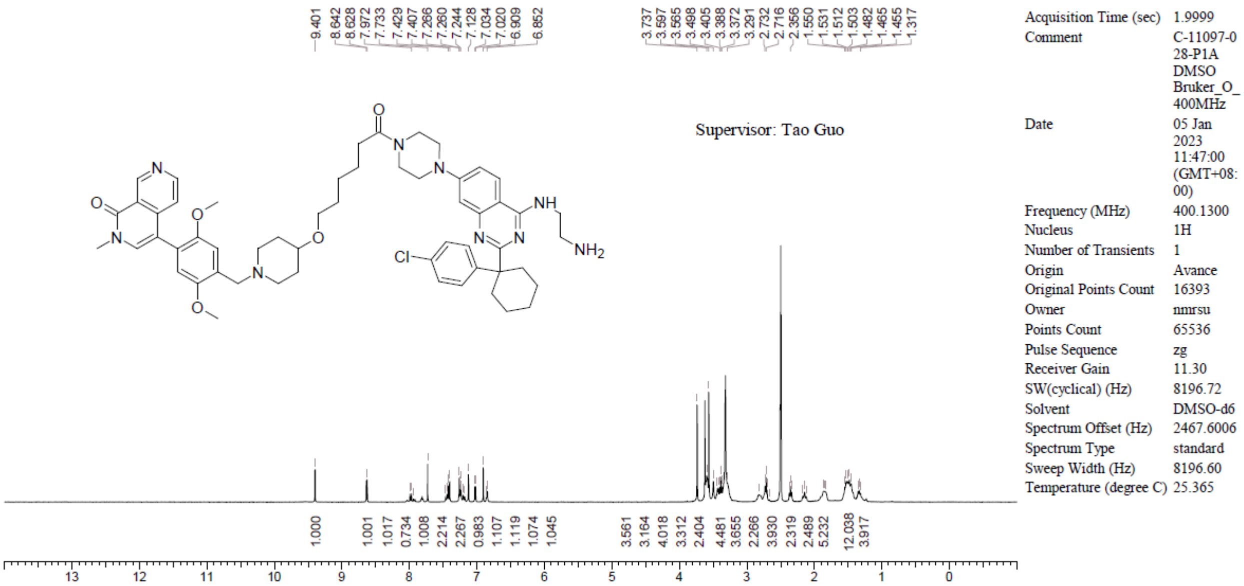

Supplementary spectrum 8: 1H-NMR spectrum of **DBr-1**

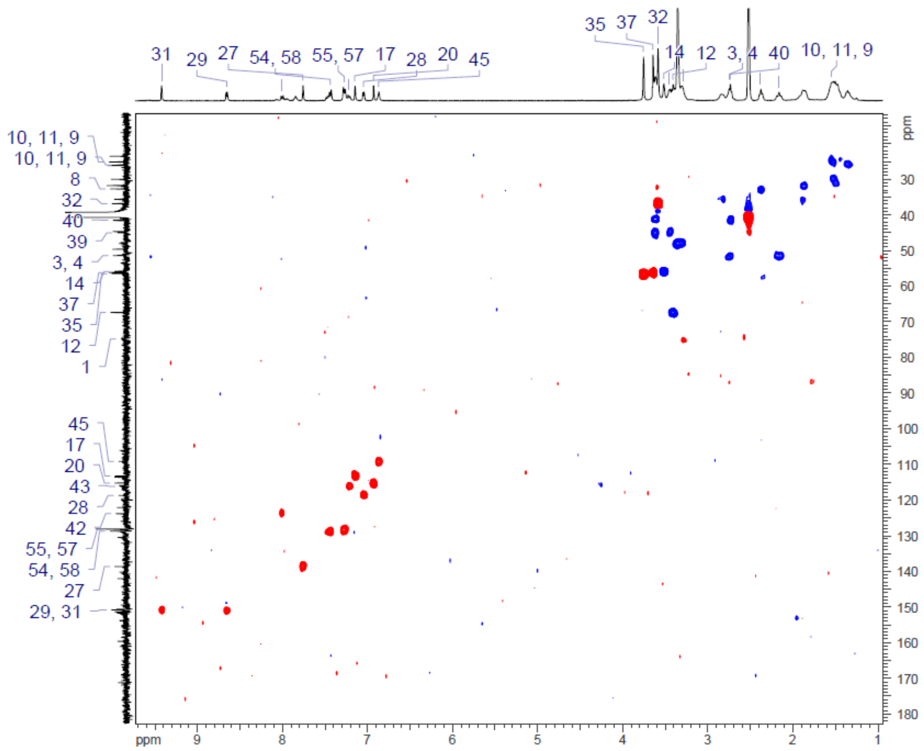

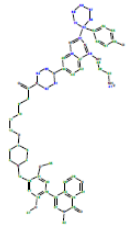

Acquisition Time (sec)	(0.1828, 0.0064)
Comment	C-11097-028-P1B DMSO
Date	06 Jan 2023 02:55:18
Frequency (MHz)	(399.9340, 100.5635)
Nucleus	(1H, 13C)
Number of Transients	4
Original Points Count	(1024, 128)
Points Count	(2048, 512)
Pulse Sequence	hsqc_edetgp
Solvent	DMSO-d6
Spectrum Type	HSQC-DEPT
Sweep Width (Hz)	(5598.27, 20074.71)
Temperature (degree C)	21.350
Title	C-11097-028-P1B DMSO

Supplementary spectrum 9: HSQC-NMR spectrum of **DBr-1**

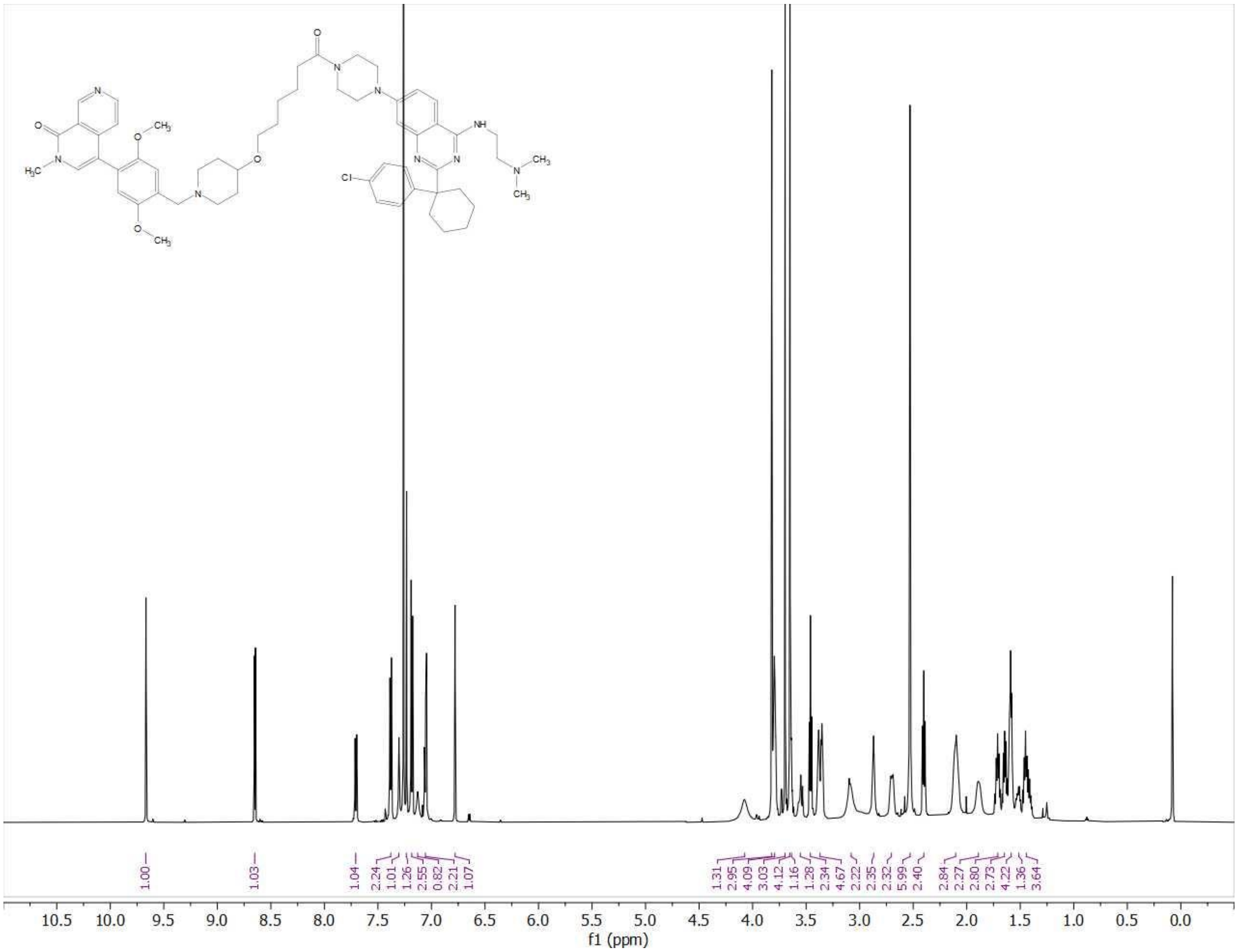

Supplementary spectrum 10: ^{1}H-NMR spectrum of **DBr-1-N**

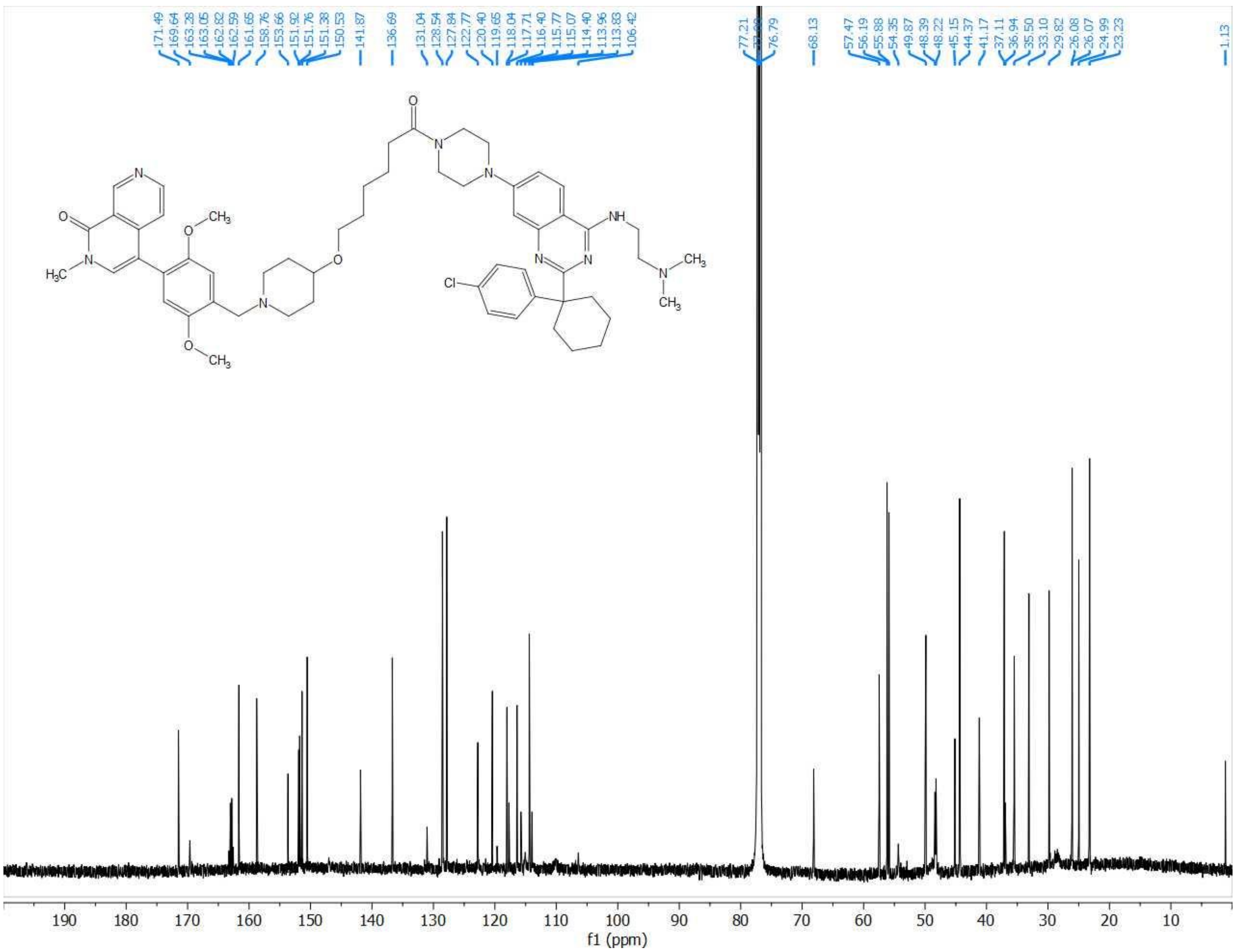

Supplementary spectrum 11: ^{13}C-NMR spectrum of **DBr-1-N**

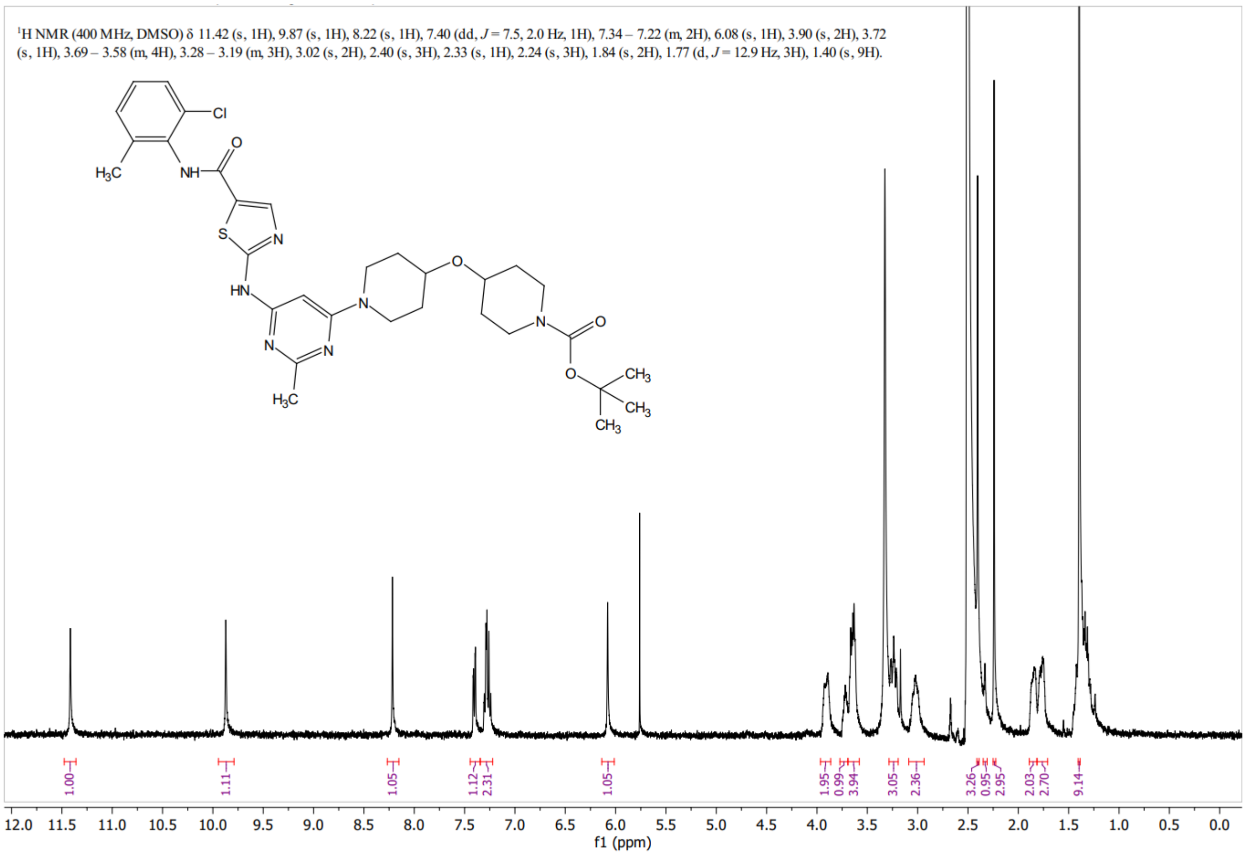

Supplementary spectrum 12: ^{1}H-NMR spectrum of **Tert-butyl 4-((1-(6-((5-((2-chloro-6-methylphenyl)carbamoyl)thiazol-2-yl)amino)-2-methylpyrimidin-4-yl)piperidin-4-yl)oxy)piperidine-1-carboxylate**

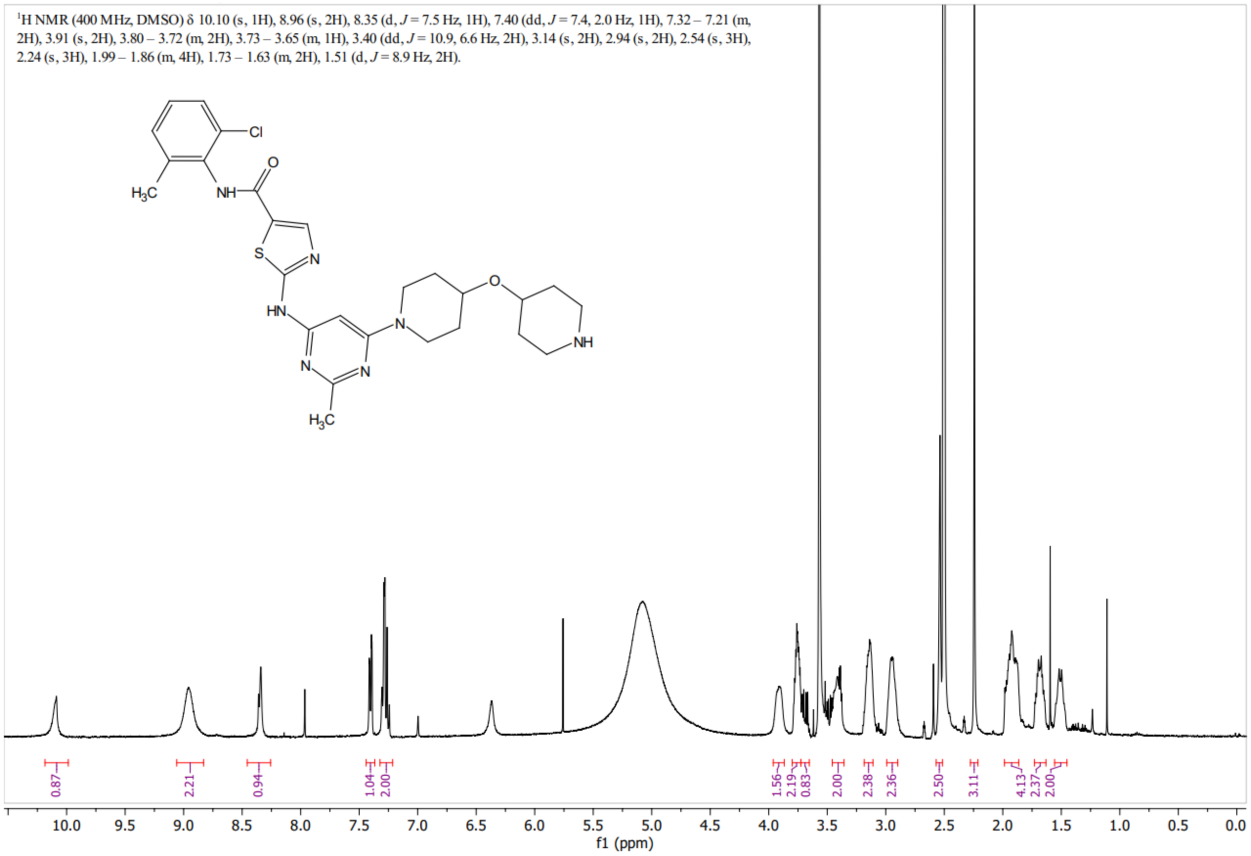

Supplementary spectrum 13: ¹H-NMR spectrum of **N-(2-chloro-6-methylphenyl)-2-((2-methyl-6-(4-(piperidin-4-yloxy)piperidin-1-yl)pyrimidin-4-yl)amino)thiazole-5-carboxamide**

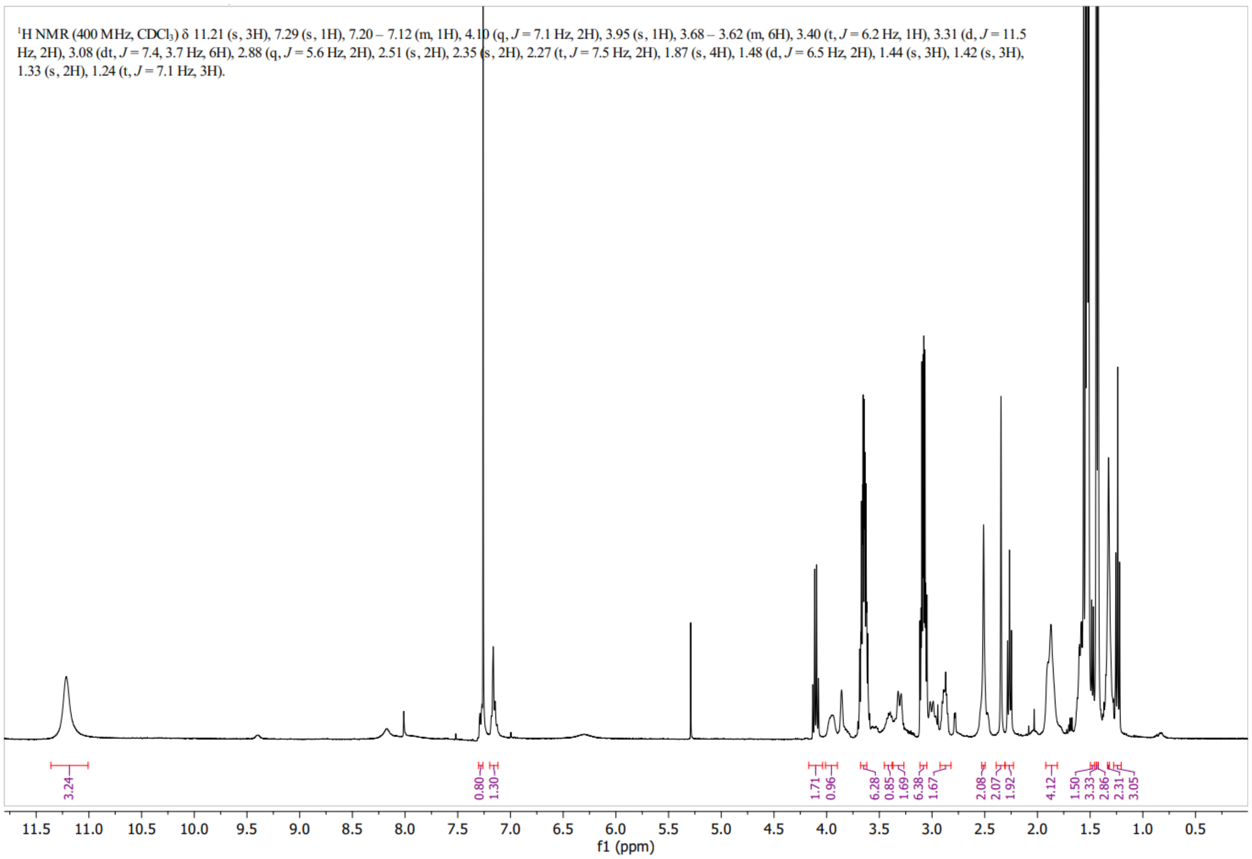

Supplementary spectrum 14: ^{1}H-NMR spectrum of **Ethyl 8-(4-((1-(6-((5-((2-chloro-6-methylphenyl)carbamoyl)thiazol-2-yl)amino)-2-methylpyrimidin-4-yl)piperidin-4-yl)oxy)piperidin-1-yl)octanoate**

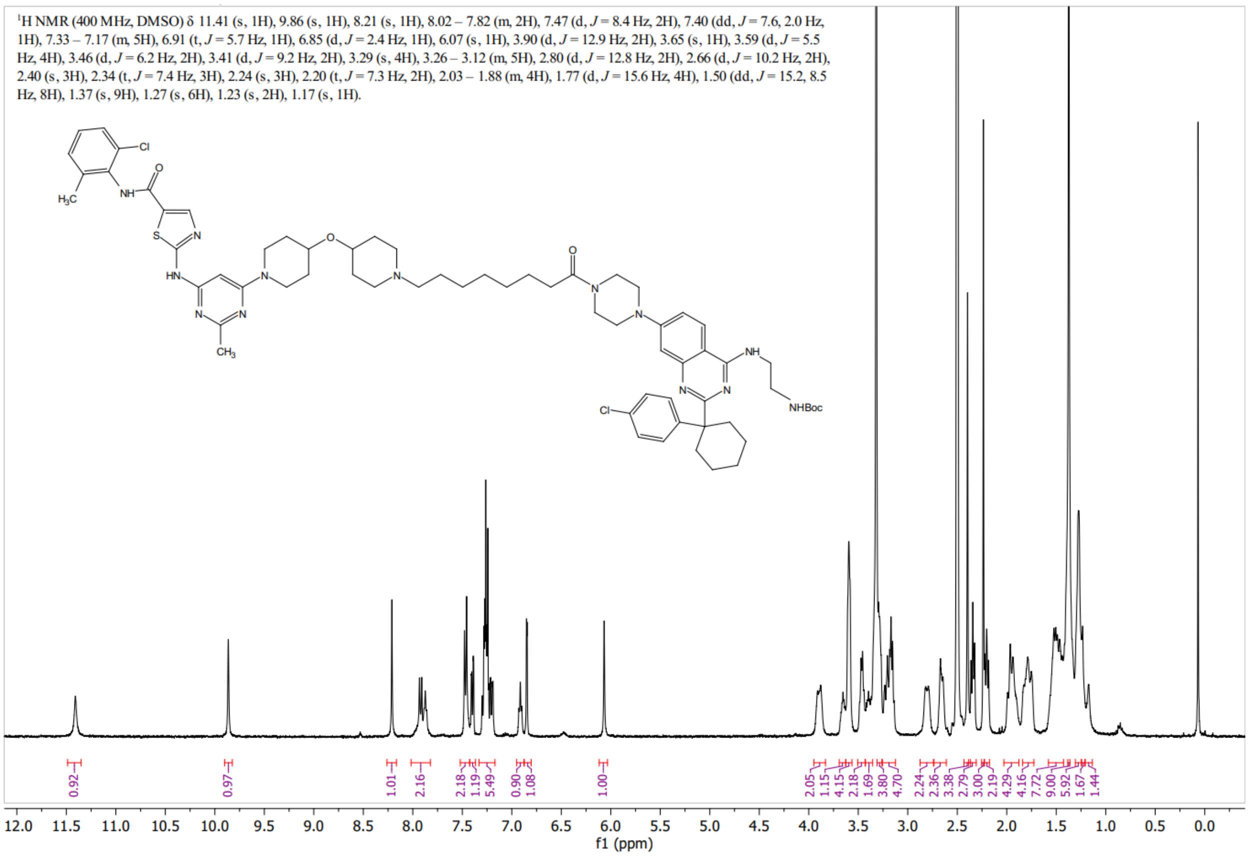

Supplementary spectrum 15: ^{1}H-NMR spectrum of **Tert-butyl (2-((7-(4-(8-(4-((1-(6-((5-((2-chloro-6-methylphenyl)carbamoyl)thiazol-2-yl)amino)-2-methylpyrimidin-4-yl)piperidin-4-yl)oxy)piperidin-1-yl)octanoyl)piperazin-1-yl)-2-(1-(4-chlorophenyl)cyclohexyl)quinazolin-4-yl)amino)ethyl)carbamate**

^{1}H NMR (400 MHz, DMSO) δ 13.39 (d, J = 5.2 Hz, 1H), 10.75 (s, 1H), 10.26 (s, 1H), 10.13 (s, 1H), 8.47 (d, J = 9.4 Hz, 1H), 8.37 (s, 3H), 7.67 (d, J = 4.0 Hz, 1H), 7.57 (dd, J = 8.7, 1.6 Hz, 2H), 7.41 – 7.36 (m, 4H), 7.31 – 7.22 (m, 2H), 3.94 (dd, J = 37.3, 11.0 Hz, 4H), 3.76 – 3.60 (m, 5H), 3.50 (ddd, J = 4.9, 4.1, 1.4 Hz, 3H), 3.48 – 3.36 (m, 6H), 3.26 (d, J = 11.7 Hz, 1H), 3.16 (d, J = 6.1 Hz, 2H), 2.96 (q, J = 12.9 Hz, 5H), 2.54 (s, 3H), 2.35 (t, J = 7.4 Hz, 2H), 2.24 (s, 3H), 2.12 (d, J = 6.0 Hz, 2H), 2.03 (s, 2H), 1.89 (s, 3H), 1.74 – 1.58 (m, 4H), 1.46 (d, J = 40.0 Hz, 8H), 1.32 (d, J = 17.9 Hz, 8H), 0.92 – 0.82 (m, 1H).

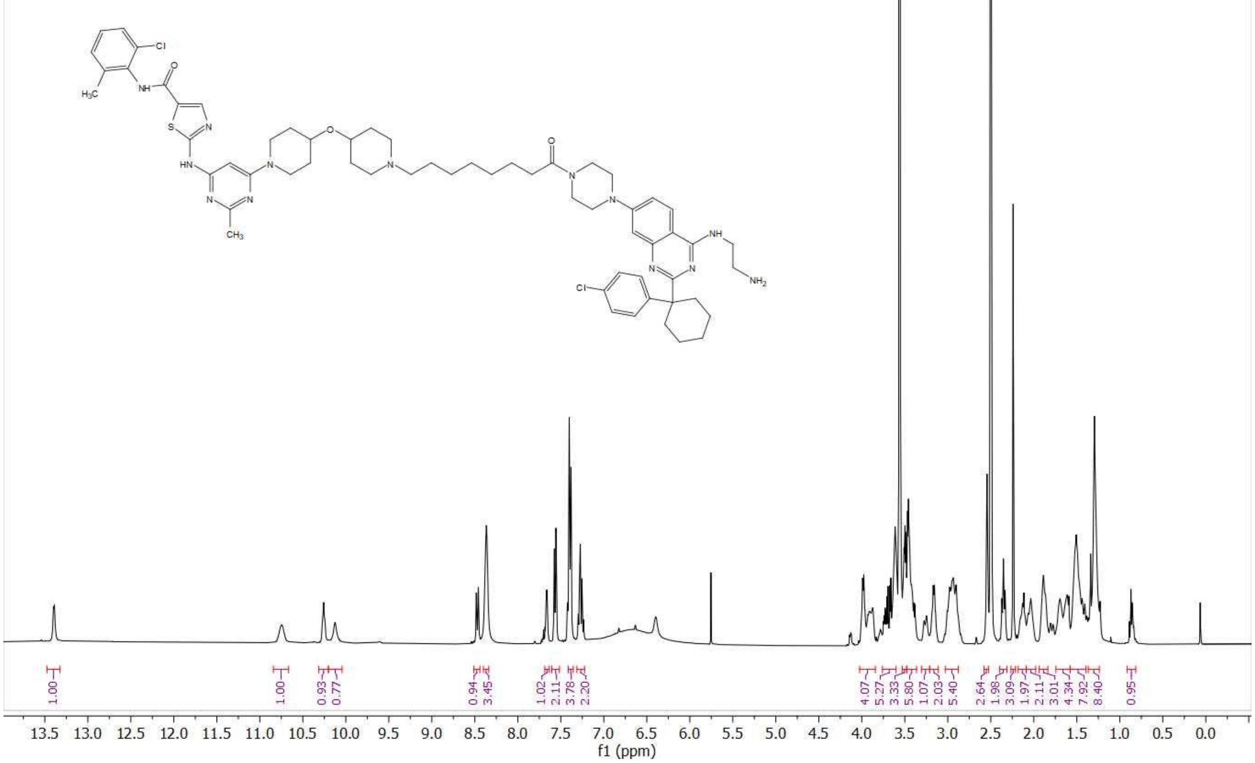

Supplementary spectrum 16: ^{1}H-NMR spectrum of **DDa-1**

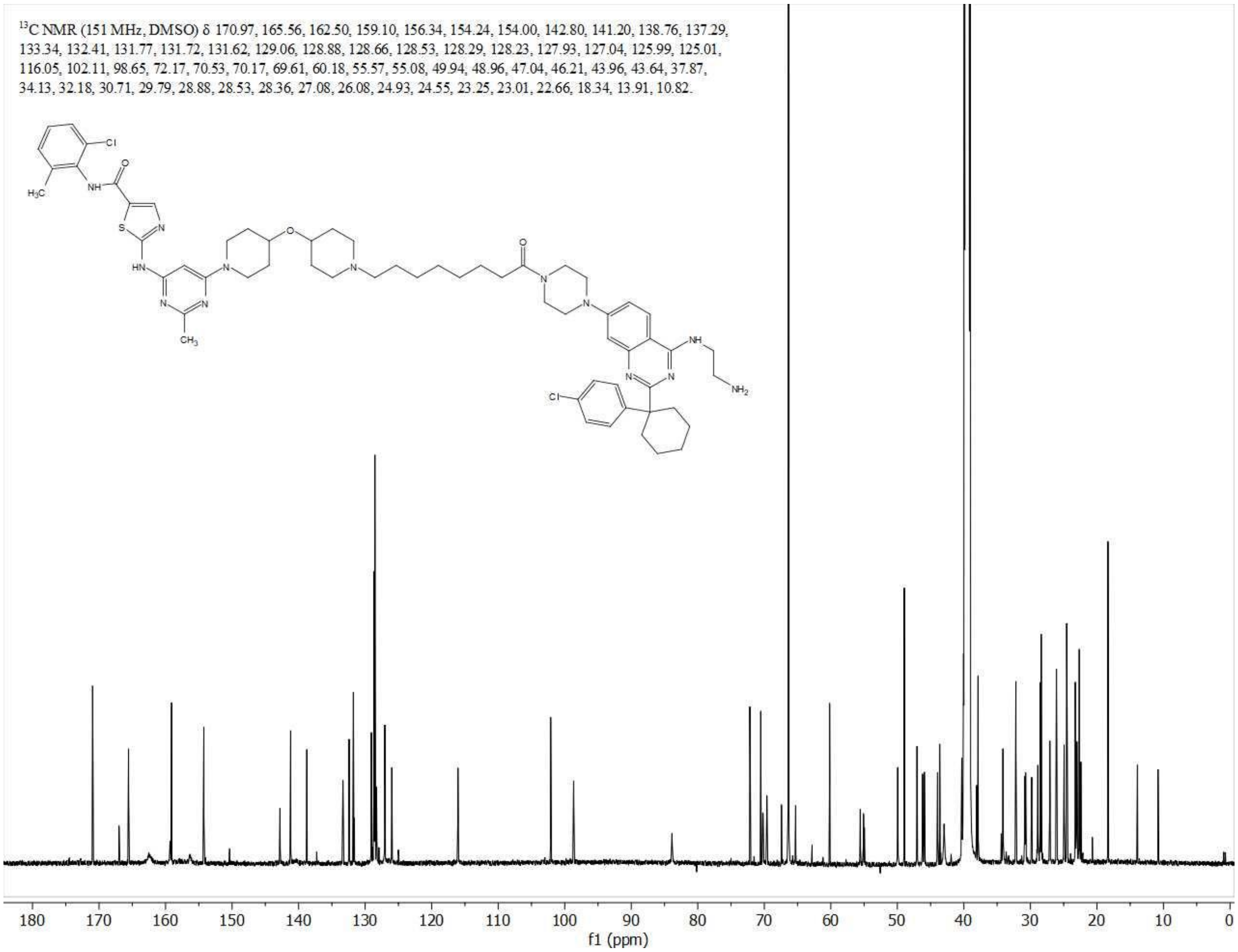

Supplementary spectrum 17: ^{13}C-NMR spectrum of **DDa-1**

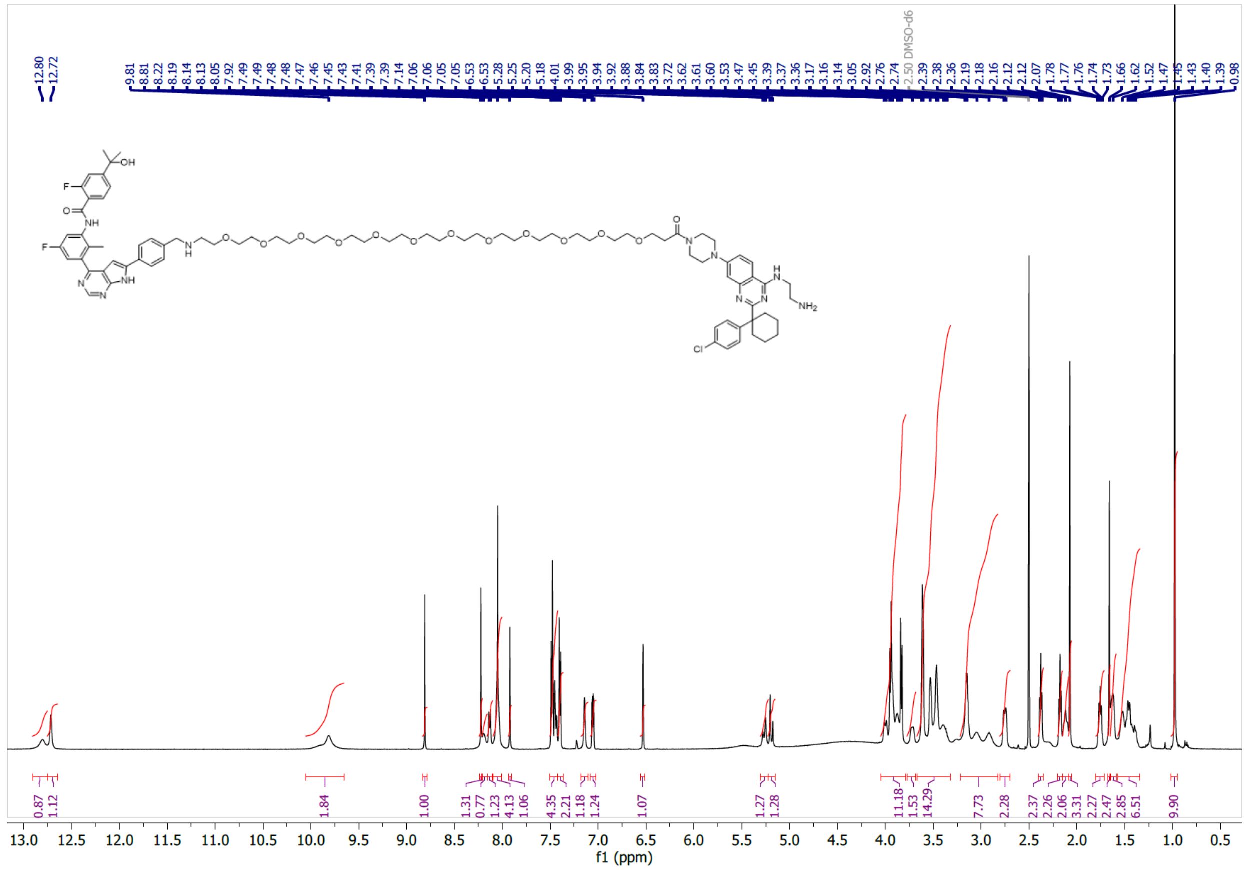

Supplementary spectrum 18: ^{1}H-NMR spectrum of **DBt-5**

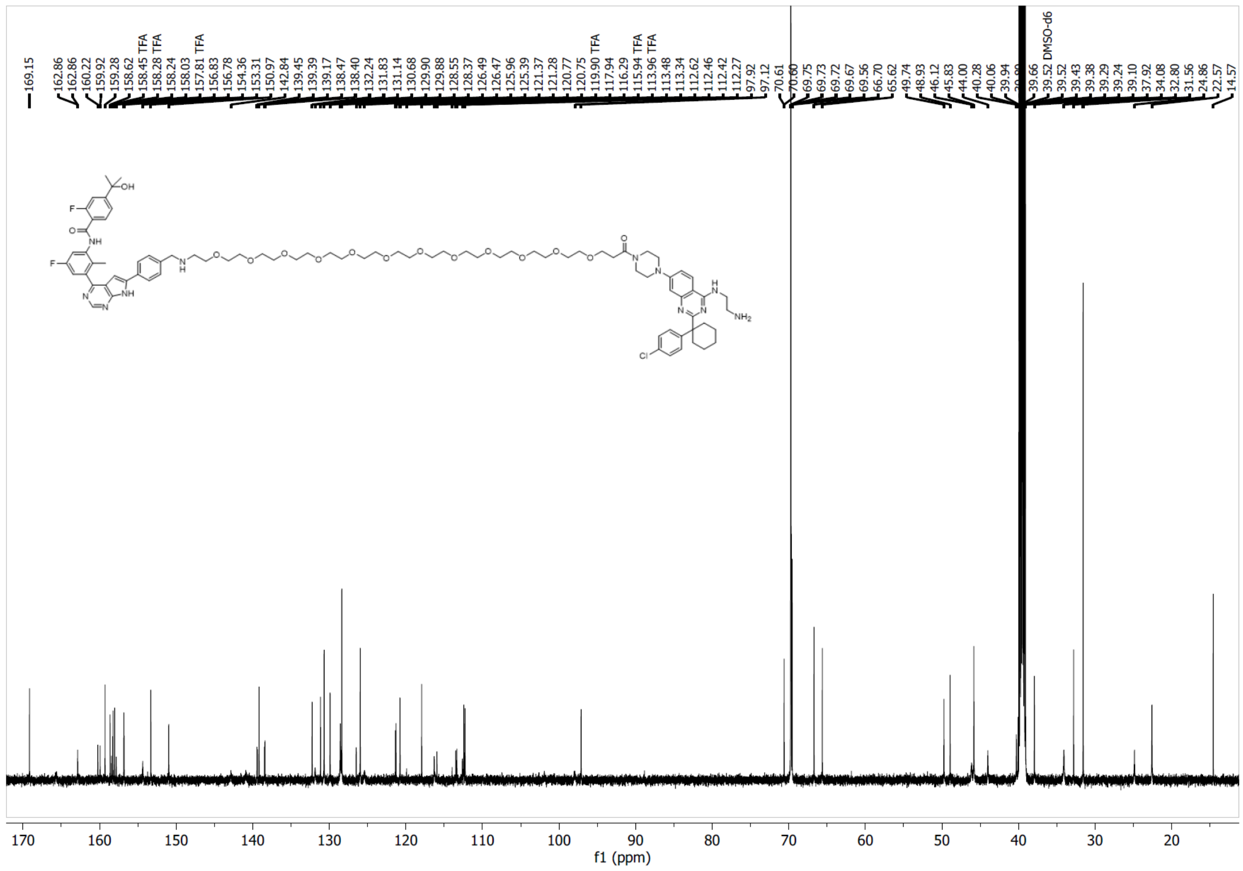

Supplementary spectrum 19: ^{13}C-NMR spectrum of **DBt-5**

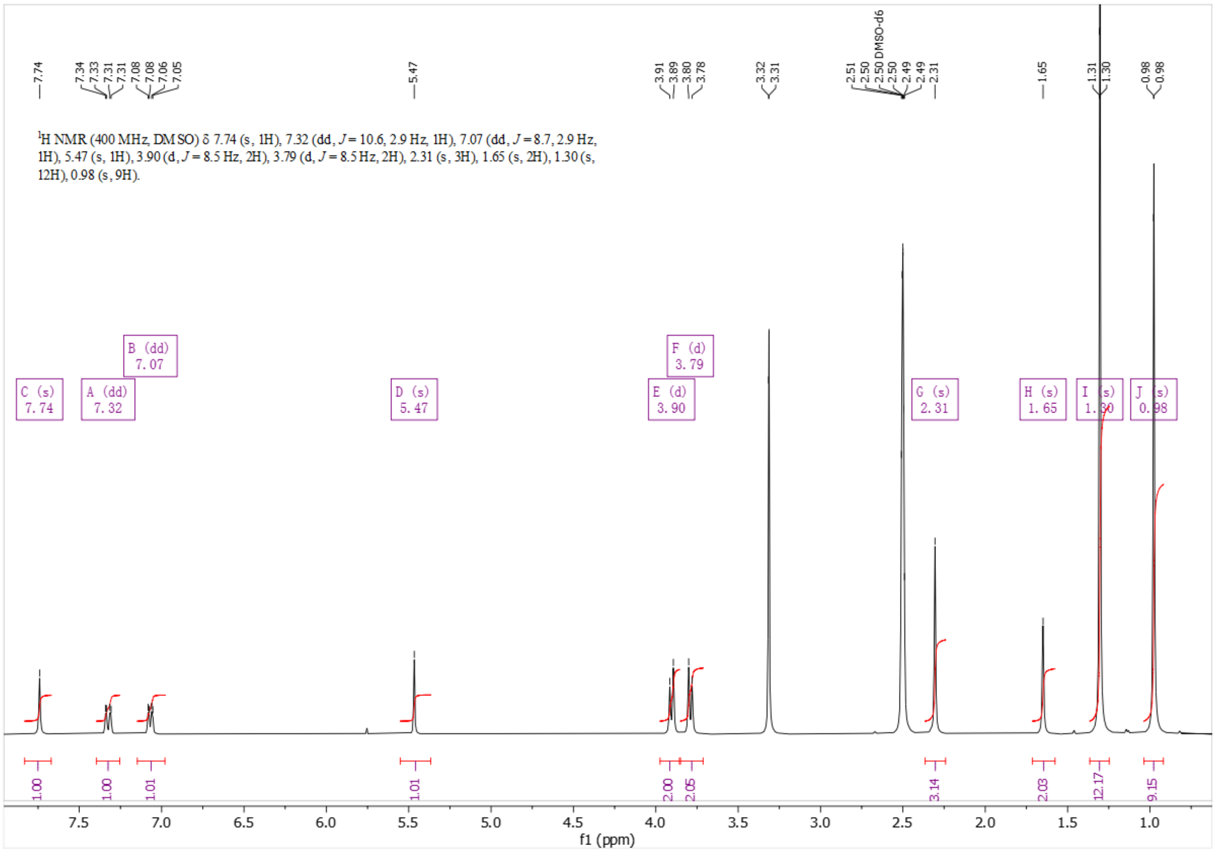

Supplementary spectrum 20: ^{13}C-NMR spectrum of **N-(5-fluoro-2-methyl-3-(4,4,5,5-tetramethyl-1,3,2-dioxaborolan-2-yl)phenyl)-3-hydroxy-3-neopentylazetidine-1-carboxamide**

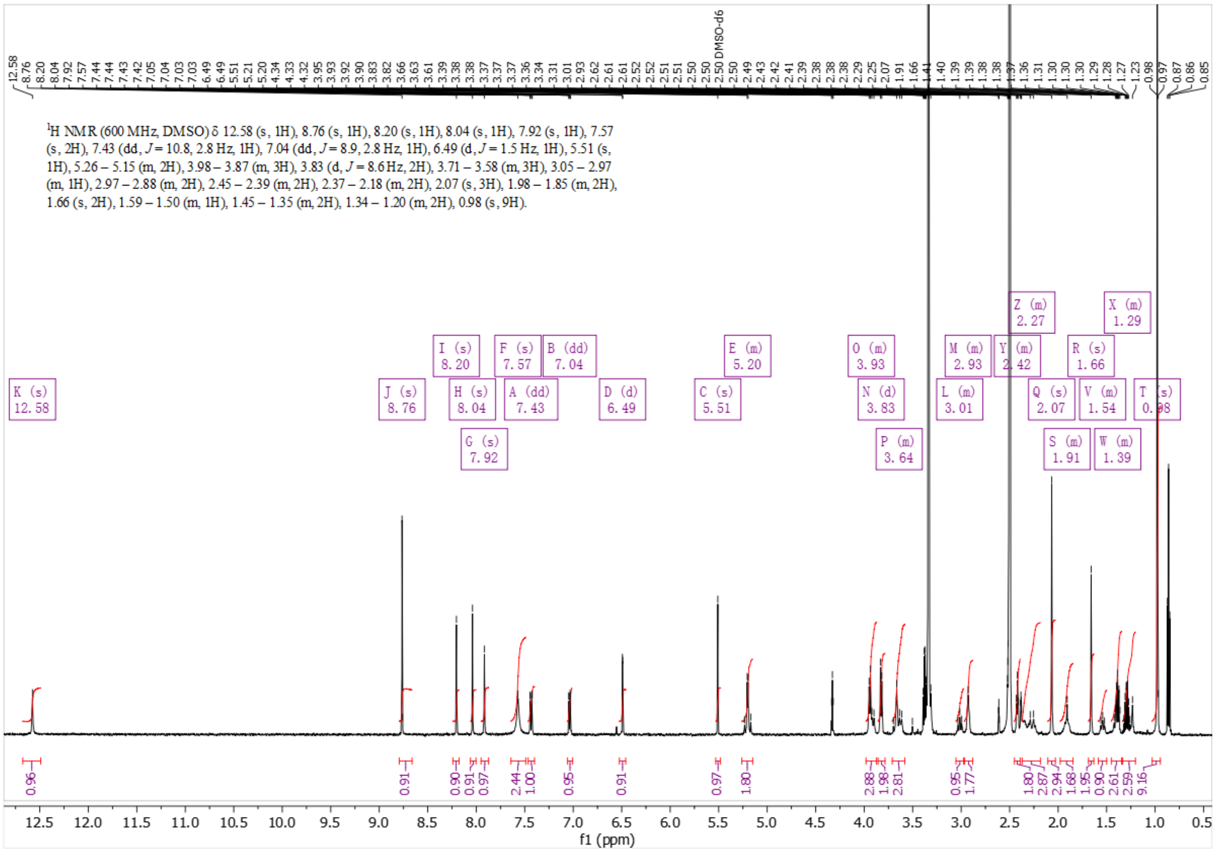

Supplementary spectrum 21: ^{1}H-NMR spectrum of **N-(3-(6-(1-(2-(4-(2-aminoethyl)-1-oxa-4,9-diazaspiro[5.5]undecan-9-yl)-2-oxoethyl)-1H-pyrazol-4-yl)-7H-pyrrolo[2,3-d]pyrimidin-4-yl)-5-fluoro-2-methylphenyl)-3-hydroxy-3-neopentylazetidine-1-carboxamide**

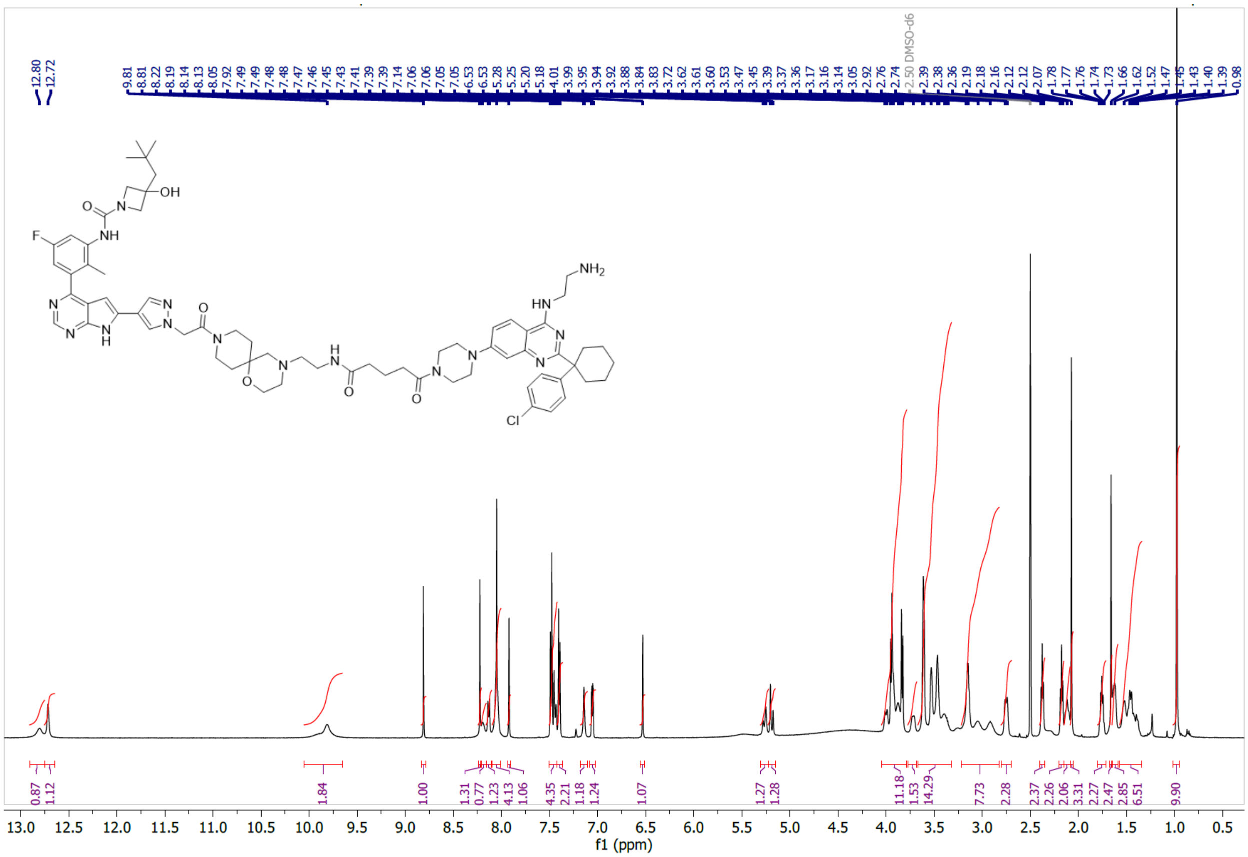

Supplementary spectrum 22: ^{1}H-NMR spectrum of **DBt-10**

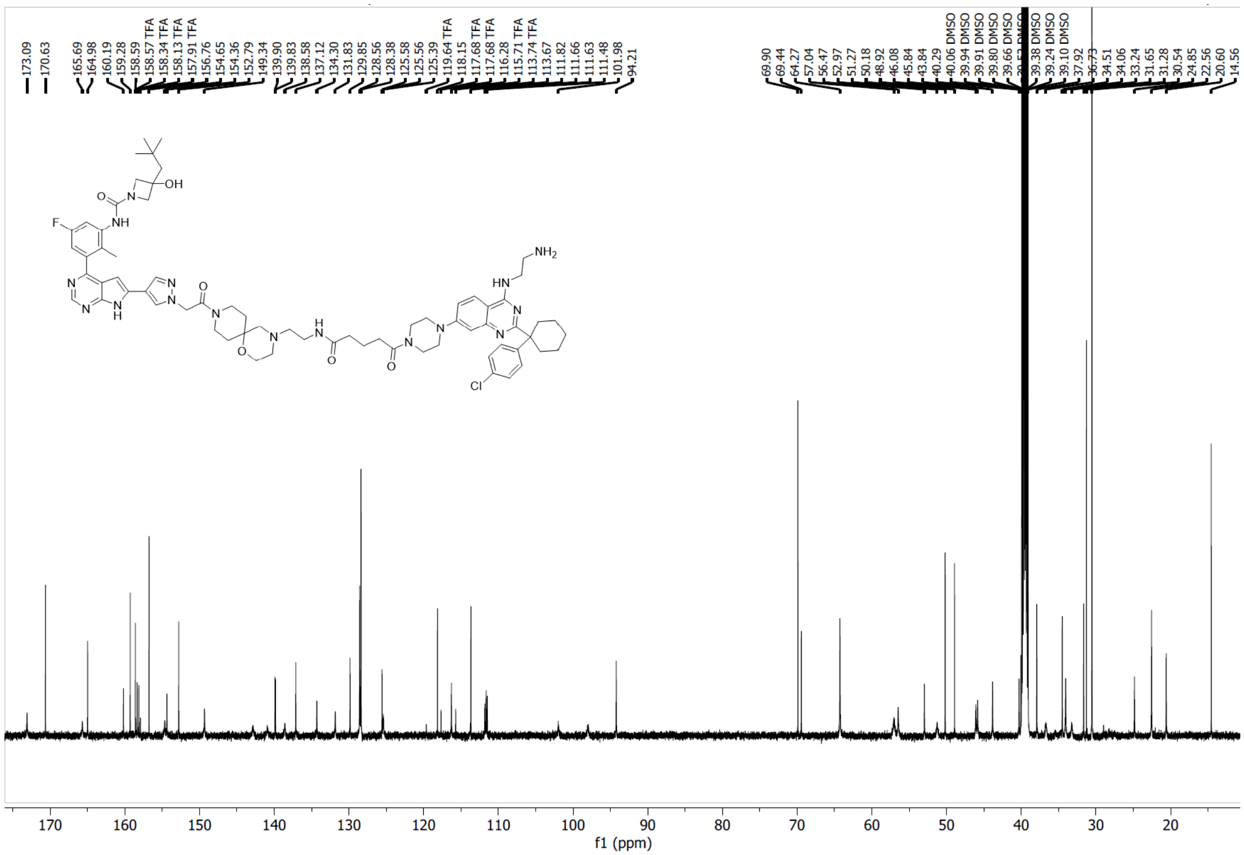

Supplementary spectrum 23: ^{13}C-NMR spectrum of **DBt-10**

References:

1. Li, A.S.M., et al., *Discovery of Nanomolar DCAF1 Small Molecule Ligands.* J Med Chem, 2023.
2. Vulpetti, A., et al., *Discovery of New Binders for DCAF1, an Emerging Ligase Target in the Targeted Protein Degradation Field.* ACS Medicinal Chemistry Letters, 2023. **14**(7): p. 949-954.
