# Supplementary material for: DCAF1-based PROTACs with activity against clinically validated targets overcoming intrinsic- and acquired-degrader resistance
Source: Nat Commun. 2024 Jan 4;15:275. doi: 10.1038/s41467-023-44237-4 (PMC10766610; doi:10.1038/s41467-023-44237-4)
Supplement: Supplementary file 13 — Reporting Summary [file 41467_2023_44237_MOESM13_ESM.pdf]

nature portfolio

Corresponding author(s): Martin Schröder, Claudio Thoma

Last updated by author(s): Nov 28, 2023

Reporting Summary

Nature Portfolio wishes to improve the reproducibility of the work that we publish. This form provides structure for consistency and transparency in reporting. For further information on Nature Portfolio policies, see our Editorial Policies and the Editorial Policy Checklist.

Statistics

For all statistical analyses, confirm that the following items are present in the figure legend, table legend, main text, or Methods section.

n/a	Confirmed	
	x	The exact sample size (*n*) for each experimental group/condition, given as a discrete number and unit of measurement
x		A statement on whether measurements were taken from distinct samples or whether the same sample was measured repeatedly
	x	The statistical test(s) used AND whether they are one- or two-sided<br>*Only common tests should be described solely by name; describe more complex techniques in the Methods section.*
x		A description of all covariates tested
x		A description of any assumptions or corrections, such as tests of normality and adjustment for multiple comparisons
	x	A full description of the statistical parameters including central tendency (e.g. means) or other basic estimates (e.g. regression coefficient) AND variation (e.g. standard deviation) or associated estimates of uncertainty (e.g. confidence intervals)
	x	For null hypothesis testing, the test statistic (e.g. *F*, *t*, *r*) with confidence intervals, effect sizes, degrees of freedom and *P* value noted<br>*Give P values as exact values whenever suitable.*
x		For Bayesian analysis, information on the choice of priors and Markov chain Monte Carlo settings
x		For hierarchical and complex designs, identification of the appropriate level for tests and full reporting of outcomes
x		Estimates of effect sizes (e.g. Cohen's *d*, Pearson's *r*), indicating how they were calculated

Our web collection on statistics for biologists contains articles on many of the points above.

Software and code

Policy information about availability of computer code

Data collection	Chromeleon v.6.8<br>CytExpert (Beckmann) v.2.4

Data analysis	Adobe Illustrator Adobe Creative Cloud 27.8.1<br>Fiji ImageJ 1.53t<br>Proteome Discoverer 2.4 Thermo Scientific<br>Spotfire Tibco v. 12<br>Python Anaconda v 3.6.10<br>Numpy Anaconda v 1.17.2<br>PyMOL v 2.5.2<br>AIMLESS v. 0.7.9<br>CCP4I2 v 7.9<br>Phaser 2.8.3<br>Coot 0.9.8.7<br>Refmac5 5.8.0411<br>MolProbity 4.5.1<br>Graphpad PRISM v. 9.5.1<br>Compass for Simple Western v. 5.0.1

For manuscripts utilizing custom algorithms or software that are central to the research but not yet described in published literature, software must be made available to editors and reviewers. We strongly encourage code deposition in a community repository (e.g. GitHub). See the Nature Portfolio guidelines for submitting code & software for further information.

Data

Policy information about availability of data

All manuscripts must include a data availability statement. This statement should provide the following information, where applicable:

- Accession codes, unique identifiers, or web links for publicly available datasets
- A description of any restrictions on data availability
- For clinical datasets or third party data, please ensure that the statement adheres to our policy

Source data are provided with this paper. The full list of E3 ligase with WDR motifs is attached as Supplementary Table 1. The raw data for the proteomics study is attached as Supplementary Table 2,6 and 8. The raw data for the arrayed CRISPR rescue screen is attached as Supplementary Table 4. All compound smiles can be found in Supplementary Table 9. The coordinates and structure factors of the DCAF1-13 and DCAF1-15 complexes have been uploaded to the PDB (PDB IDs: 8OO5 [RCSB PDB - 8OO5: Crystal structure of human DCAF1 WD40 repeats (Q1250L) in complex with compound 13] and 8OOD [RCSB PDB - 8OOD: Crystal structure of human DCAF1 WD40 repeats (Q1250L) in complex with compound 15]). All proteomic data has been uploaded to PRIDE under the identifier PXD046286 (DOI 10.6019/PXD046286) [https://www.doi.org/10.6019/PXD046286] and PXD047347 [https://www.doi.org/10.6019/PXD047347]

Research involving human participants, their data, or biological material

Policy information about studies with human participants or human data. See also policy information about sex, gender (identity/presentation), and sexual orientation and race, ethnicity and racism.

Reporting on sex and gender	n/a
Reporting on race, ethnicity, or other socially relevant groupings	n/a
Population characteristics	n/a
Recruitment	n/a
Ethics oversight	n/a

Note that full information on the approval of the study protocol must also be provided in the manuscript.

Field-specific reporting

Please select the one below that is the best fit for your research. If you are not sure, read the appropriate sections before making your selection.

☒ Life sciences ☐ Behavioural & social sciences ☐ Ecological, evolutionary & environmental sciences

For a reference copy of the document with all sections, see nature.com/documents/nr-reporting-summary-flat.pdf

Life sciences study design

All studies must disclose on these points even when the disclosure is negative.

Sample size	Unless otherwise stated at least three replicates for each experiment were performed. More details can be found in the respective Figure sections, the Method section and the Statistics and Reproducibility section

Data exclusions	See Method section for systematic data exclusion (e.g., for the FACS analysis if cell viability was below 25%). Other excluded data are shown and highlighted in Source Data file
Replication	All our attempts to reproduce the data shown here were successful
Randomization	No large dataset where systematic bias could have impacted the data analysis were used in this study. Thus no randomisation was necessary.
Blinding	No analytic bias could be identified in the datasets used in this study and thus blinding was not necessary.

Reporting for specific materials, systems and methods

We require information from authors about some types of materials, experimental systems and methods used in many studies. Here, indicate whether each material, system or method listed is relevant to your study. If you are not sure if a list item applies to your research, read the appropriate section before selecting a response.

Materials & experimental systems

n/a	Involved in the study
☐	☒ Antibodies
☐	☒ Eukaryotic cell lines
☒	☐ Palaeontology and archaeology
☒	☐ Animals and other organisms
☒	☐ Clinical data
☒	☐ Dual use research of concern
☒	☐ Plants

Methods

n/a	Involved in the study
☒	☐ ChIP-seq
☐	☒ Flow cytometry
☒	☐ MRI-based neuroimaging

Antibodies

Antibodies used

IRDye 680RD Goat anti-Mouse secondary antibody LI-COR Cat # 926-68070 1:10000
IRDye 800CW Goat anti-Rabbit secondary antibody LI-COR Cat # 926-32211 1:10000
HRP Goat anti-rabbit secondary antibody Abcam Cat # 7090 1:10000
HRP Sheep anti-mouse secondary antibody Cytiva Cat # NA931V 1:10000
Rabbit polyclonal anti-BRD9 Bethyl Laboratories Cat # A303-781A 1:200
Rabbit anti-BRD7 monoclonal antibody Cell Signaling Technology Cat # 15125S 1:1000
Mouse monoclonal anti-actin Cell Signaling Technology Cat # 3700 1:1000
Rabbit polyclonal anti-actin Cell Signaling Technology Cat # 4970 1:1000
Mouse monoclonal anti-CAS9 Cell Signaling Technology Cat # 14697 1:1000
Rabbit monoclonal anti-LYN Cell Signaling Technology Cat # 2796 1:1000
Rabbit monoclonal anti-LIMK2 Cell Signaling Technology Cat# 3845 1:1000
Rabbit polyclonal anti-c-ABL Cell Signaling Technology Cat # 2862 1:1000
Rabbit monoclonal anti-CSK Cell Signaling Technology Cat # 4980 1:1000
Mouse monoclonal anti-α-tubulin Cell Signaling Technology Cat # 3873 1:1000
Rabbit polyclonal anti-BTK Cell Signaling Technology Cat # 8547 1:1000
Rabbit polyclonal anti-CYLD Cell Signaling Technology Cat # 4495 1:1000
Rabbit monoclonal anti-α-tubulin Cell Signaling Technology Cat # 2125 1:1000
Mouse monoclonal anti-a-tubulin Abcam Cat# AB7291 1:200
Rabbit polyclonal anti-DCAF1/VprBP Novus Cat # NBP1-05953 1:1000
Anti-Mouse Detection Module Bio-Techne #DM-002 1:1
Anti-Rabbit Detection Module Bio-Techne #DM-001 1:1
Rabbit monoclonal CRBN (D8H3S) Cell Signaling Technology Cat #71810 1:1000

Validation

All commercially available, for validation data please see manufacture's website. IRDye 680RD Goat anti-Mouse secondary antibody, LI-COR Cat # 926-68070, validated for western blotting on manufacturer's website (https://www.licor.com/bio/reagents/irdye-680rd-goat-anti-mouse-igg-secondary-antibody). IRDye 800CW Goat anti-Rabbit secondary antibody. LI-COR Cat # 926-32211 , validated for western blotting on manufacturer's website (IRDye 800CW Goat anti-Rabbit IgG Secondary Antibody (licor.com)). HRP Goat anti-rabbit secondary antibody Abcam Cat # 7090, validated for western blotting on manufacturer's website (HRP Goat Anti-Rabbit (IgG) secondary antibody preadsorbed (ab7090) | Abcam). HRP Sheep anti-mouse secondary antibody, Cytiva Cat # NA931V, validated for western blotting on manufacturer's website (Amersham ECL HRP-Conjugated Antibodies | Cytiva (cytivalifesciences.com)). Rabbit anti-BRD7 monoclonal antibody, Cell Signaling Technology Cat # 15125S, validated for western blotting on manufacturer's website (BRD7 (D9K2T) Rabbit mAb | Cell Signaling Technology). Mouse monoclonal anti-actin, Cell Signaling Technology Cat # 3700, validated for western blotting on manufacturer's website (β-Actin (8H10D10) Mouse mAb | Cell Signaling Technology). Rabbit polyclonal anti-actin, Cell Signaling Technology Cat # 4970, validated for western blotting on manufacturer's website (β-Actin (13E5) Rabbit mAb | Cell Signaling Technology). Mouse monoclonal anti-CAS9, Cell Signaling Technology Cat # 14697, validated for western blotting on manufacturer's website (Cas9 (S. pyogenes) (7A9-3A3) Mouse mAb | Cell Signaling Technology). Rabbit monoclonal anti-LYN, Cell Signaling Technology Cat # 2796, validated for western blotting on manufacturer's website (Lyn (C13F9) Rabbit mAb | Cell Signaling Technology). Rabbit monoclonal anti-LIMK2, Cell Signaling Technology Cat# 3845, validated for western blotting on manufacturer's

website (LIMK2 (8C11) Rabbit mAb | Cell Signaling Technology). Rabbit polyclonal anti-c-ABL, Cell Signaling Technology Cat # 2862, validated for western blotting on manufacturer's website (c-Abl Antibody | Cell Signaling Technology). Rabbit monoclonal anti-CSK, Cell Signaling Technology Cat # 4980, validated for western blotting on manufacturer's website (Csk (C74C1) Rabbit mAb | Cell Signaling Technology). Mouse monoclonal anti-α-tubulin, Cell Signaling Technology Cat # 3873, validated for western blotting on manufacturer's website (alpha Tubulin Antibody (DM1A) | Cell Signaling Technology). Rabbit polyclonal anti-BTK, Cell Signaling Technology Cat # 8547, validated for western blotting on manufacturer's website (Btk (D3H5) Rabbit mAb | Cell Signaling Technology). Rabbit polyclonal anti-CYLD, Cell Signaling Technology Cat # 4495, validated for western blotting on manufacturer's website (CYLD Antibody | Cell Signaling Technology). Rabbit monoclonal anti-α-tubulin, Cell Signaling Technology Cat # 2125, validated for western blotting on manufacturer's website (α-Tubulin (11H10) Rabbit mAb | Cell Signaling Technology). Mouse monoclonal anti-a-tubulin, Abcam Cat# AB7291, validated for western blotting on manufacturer's website (Anti-alpha Tubulin antibody [DM1A] - Loading Control (ab7291) | Abcam). Rabbit polyclonal anti-DCAF1/VprBP, Novus Cat # NBP1-05953, validated for western blotting on manufacturer's website (VprBP Antibody (NBP1-05953): Novus Biologicals). Anti-Mouse Detection Module, Bio-Techne #DM-002, validated for capillary western blotting on manufacturer's website (Anti-Mouse Detection Module (DM-002) | Bio-Techne). Anti-Rabbit Detection Module, Bio-Techne #DM-001, validated for capillary western blotting on manufacturer's website (Anti-Rabbit Detection Module (DM-001) | Bio-Techne). Rabbit monoclonal CRBN (D8H3S), Cell Signaling Technology Cat #71810, validated for western blotting on manufacturer's website (CRBN (D8H3S) Rabbit mAb | Cell Signaling Technology).

Eukaryotic cell lines

Policy information about cell lines and Sex and Gender in Research

Cell line source(s)	HEK293T (ATCC CRL-3216™)<br>HEK293 BRD9-HiBIT pool (Source: PROMEGA CS3023119)<br>HEK293 BRD9-HiBIT FF/CAS9 (Source: this study)<br>HEK293FT (Source :Invitrogen R70007)<br>TMD8 (Source: Prof. Tohda, published in PMID: 16780947)<br>TMD8 BTK-GFP/mCh (Source: this study)<br>HEK293/BRD7-HiBiT pool (Source PROMEGA CS3023120)<br>786-O (Source: ATCC CRL-1932)
Authentication	None of the cell lines were authenticated
Mycoplasma contamination	Cells were not tested for mycoplasma contamination
Commonly misidentified lines<br>(See ICLAC register)	None of the used cell lines in this study is among the list of commonly misidentified lines

Flow Cytometry

Plots

Confirm that:

- [x] The axis labels state the marker and fluorochrome used (e.g. CD4-FITC).
- [x] The axis scales are clearly visible. Include numbers along axes only for bottom left plot of group (a 'group' is an analysis of identical markers).
- [x] All plots are contour plots with outliers or pseudocolor plots.
- [ ] A numerical value for number of cells or percentage (with statistics) is provided.

Methodology

Sample preparation	For flow cytometry measurements, 25'000 exponentially growing TMD8 BTK-GFP/mCh cells were plated in 100uL of each well of a 96-well plate and grown for 24 hours. Cells were then dosed with corresponding compounds and doses from 10mM DMSO stock for 24 hours
Instrument	Beckmann Coulter Cytoflex
Software	CytExpert (Beckmann)
Cell population abundance	Sample Protein Stability Medians were calculated if samples had at least X% viability (whatever the value we ended up picking was) out of ~25'000 cells (the spreadsheet should contain the exact number here) as measured by shift outside of the SSC/FSC gate (see result section for experimental validation)
Gating strategy	Cells were gated based on FSC/SSC of DMSO-treated population to contain majority of events. Cells were then gated for single-cells based on SSC-A vs SSC-H. No staining was performed for the experiment, the barrier for GFP/mCherry-positive & negative was set based on parental non-transduced TMD8 fluorescence levels.

- [x] Tick this box to confirm that a figure exemplifying the gating strategy is provided in the Supplementary Information.
