# Supplementary material for: DCAF1-based PROTACs with activity against clinically validated targets overcoming intrinsic- and acquired-degrader resistance
Source: Nat Commun. 2024 Jan 4;15:275. doi: 10.1038/s41467-023-44237-4 (PMC10766610; doi:10.1038/s41467-023-44237-4)
Supplement: Supplementary file 14 — Description of Additional Supplementary Information [file 41467_2023_44237_MOESM14_ESM.pdf]

Title: Supplementary Data 1:
Description: Genes included in the DepMap analysis

Title: Supplementary Data 2:
Description: Raw data of chemical proteomics experiment in HEK293T cells with compound (**16**)

Title: Supplementary Data 3:
Description: Details of the sgRNA library used in BRD9 degradation rescue experiment

Title: Supplementary Data 4:
Description: Raw data of the BRD9 degradation rescue experiment

Title: Supplementary Data 5:
Description: Genes classified as hits in the BRD9 degradation rescue experiment

Title: Supplementary Data 6:
Description: Raw data of the proteomics experiment comparing **DDa-1** and compound (**13**)

Title: Supplementary Data 7:
Description: Characterization data of the DCAF1 based BTK degrader and control compounds

Title: Supplementary Data 8:
Description: Raw data of the proteomics experiment comparing **DBt-10** and compound (**19**)

Title: Supplementary Data 9:
Description: Compound IDs and SMILES codes

Title: Supplementary Data 10:
Description: Key resource table
